# Supplementary material for: Controlled switching thiocarbonylthio end-groups enables interconvertible radical and cationic single-unit monomer insertions and RAFT polymerizations
Source: Nat Commun. 2024 Jun 13;15:5071. doi: 10.1038/s41467-024-49463-y (PMC11176327; doi:10.1038/s41467-024-49463-y)
Supplement: Supplementary file 1 — Supplementary Information [file 41467_2024_49463_MOESM1_ESM.pdf]

Supplementary Information

Controlled Switching Thiocarbonylthio End-Groups Enables Interconvertible Radical and Cationic Single-Unit Monomer Insertions and RAFT Polymerizations

Wei He[1,‡], Wei Tao[2,‡], Ze Wei[1], Guoming Tong[1], Xiaojuan Liu[1], Jiajia Tan[2], Sheng Yang[1], Jinming Hu[2], Guhuan Liu[1,*], and Ronghua Yang[1,*]

[‡]These authors contributed equally.

[1]Key Laboratory of Chemical Biology & Traditional Chinese Medicine Research, Ministry of Education, Institute of Interdisciplinary Studies, College of Chemistry and Chemical Engineering, Hunan Normal University, Changsha, Hunan 410081, China

[2]Department of Polymer Science and Engineering, University of Science and Technology of China, Hefei, Anhui 230026, China

*To whom correspondence should be addressed. E-mail: ghliu@hunnu.edu.cn (G.L.), Yangrh@pku.edu.cn (R.Y.)

Materials

Isobutyl vinyl ether (iBVE, Sigma), *trans*-anethole (An, Sigma), vinyl (2-chloroethyl) ether (CEVE, TCI), 4-methoxystyrene (MOS, Sinopharm), methyl acrylate (MA), acetonitrile (MeCN, Adamas-beta), and dichloromethane (DCM, Adamas-beta) were dried over CaH_2, distilled under vacuum, and then stored in glove box. Ferrocenium hexafluorophosphate ($FcPF_6$, TCI), Zinc(II) meso-tetraphenylporphine (ZnTPP, Adamas-beta), *N*-phenylmaleimide (PMI, Adamas-beta), *N*-ethylmaleimide (EMI, Adamas-beta), *N*-cyclohexylmaleimide (CyMI, Adamas-beta), 3-maleimidopropionic acid (TMI, Adamas-beta), and tetraethylthiuram disulfide (TETD, Sigma) were used as received. 4-Dimethylaminopyridine (DMAP), 1,1'-carbonyldiimidazole (CDI), and all the other reagents were purchased from Adamas-beta and used as received without further purification unless otherwise noted. Butyl (1-phenylethyl) carbonotrithioate (**S-TTC**)[1], ethyl 2-(((butylthio)carbonothioyl)thio)propanoate (**EA-TTC**)[2], butyl (1-cyanoethyl) carbonotrithioate (**ACN-TTC**)[2], ethyl 2-(((butylthio)carbonothioyl)thio)-2-methylpropanoate (**EMA-TTC**)[2], ethyl (1-isobutoxyethyl) carbonotrithioate (**iBVE-TTC**)[3], methyl 2-((diethylcarbamothioyl)thio)propanoate (**EA-DTC**)[4], 1-isobutoxyethyl diethylcarbamodithioate (**iBVE-DTC**)[5], 1-(4-methoxyphenyl)ethyl diethylcarbamodithioate (**MOS-DTC**)[6], 1-(4-phenyl)ethyl diethylcarbamodithioate (**S-DTC**)[7], bis(butylsulfanyl-thiocarbonyl) disulfide (**BBTD**)[8], bis(dodecylsulfanyl-thiocarbonyl) disulfide (**BDTD**)[8], 2,4,6-tris(4-methoxyphenyl)pyrylium tetrafluoroborate (**TMPP**)[9], and 2-(vinyloxy)ethyl benzoate (**BzEVE**)[10] were synthesized according to literature procedures.

Characterization.

All nuclear magnetic resonance (NMR) spectra were recorded on a Bruker AV400 NMR (400 MHz) spectrometer operated in the Fourier transform mode.

Electrospray ionization mass spectrometry (ESI-MS) experiment was performed on Thermo Scientific LTQ Orbitrap Mass Spectrometer equipped with an electrospray S17 interface.

Molecular weights and molecular weight distributions were determined by GPC using an SSI pump

equipped 2x Shodex GPC KD column (separation range of molecular weight from 200,000 to 500 Da) and Wyatt Optilab refractive index detector (λ = 658 nm, 35 °C). THF was used as eluent at a flow rate of 1.0 mL/min. A series of low polydispersity polystyrene standards were employed for calibration. Raw data were processed with the Astra V software (Wyatt Technology).

Matrix-assisted laser desorption/ionization-time of flight (MALDI-TOF) mass spectra were acquired on an Autoflex Speed MALDI-TOF mass spectrometer (Bruker Daltonics, Germany) equipped with a Smart beam-II laser (355 nm, 1 kHz, Bruker Daltonics).

HPLC analysis was performed with a Shimadzu HPLC system, equipped with a LC-20AP binary pump, an SPD-20A UV-vis detector, and a Symmetry C18 column.

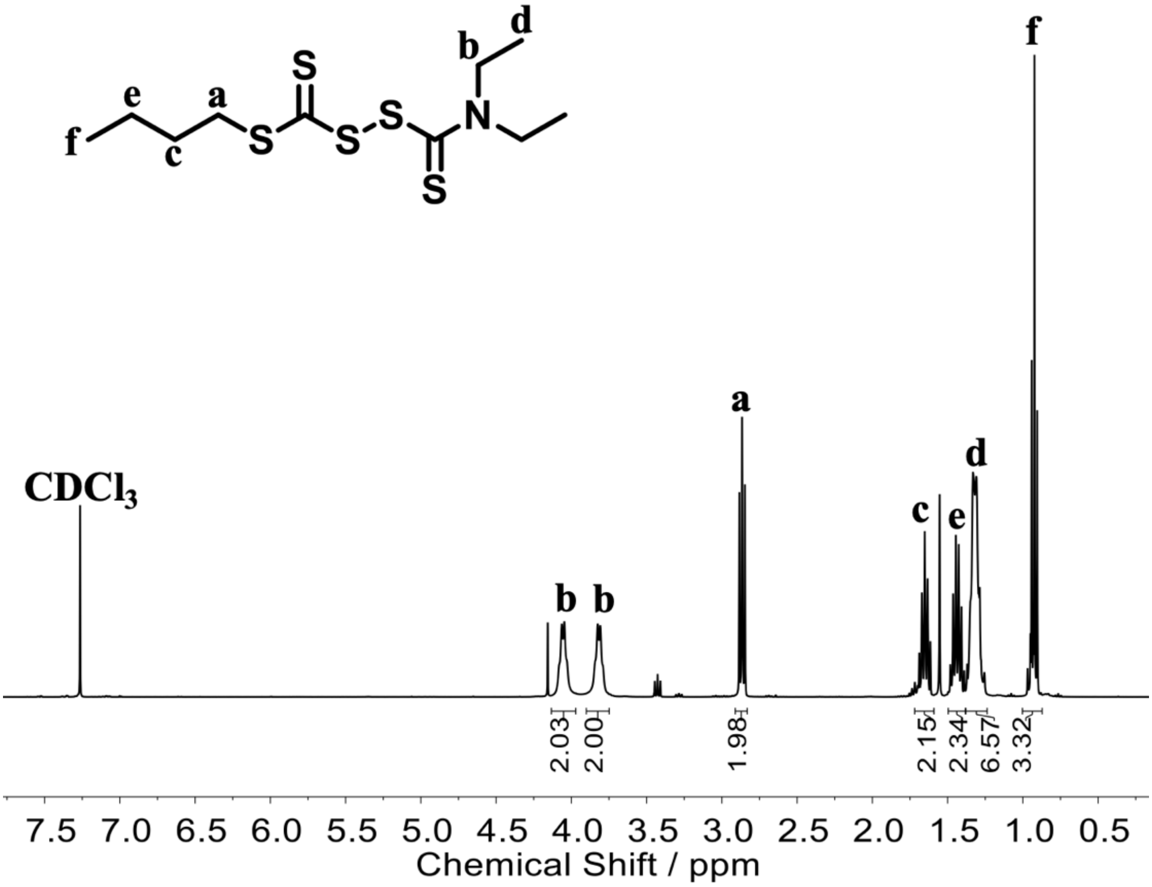

Supplementary Figure 1. ^{1}H NMR spectrum for **TTC-DTC** in $CDCl_3$.

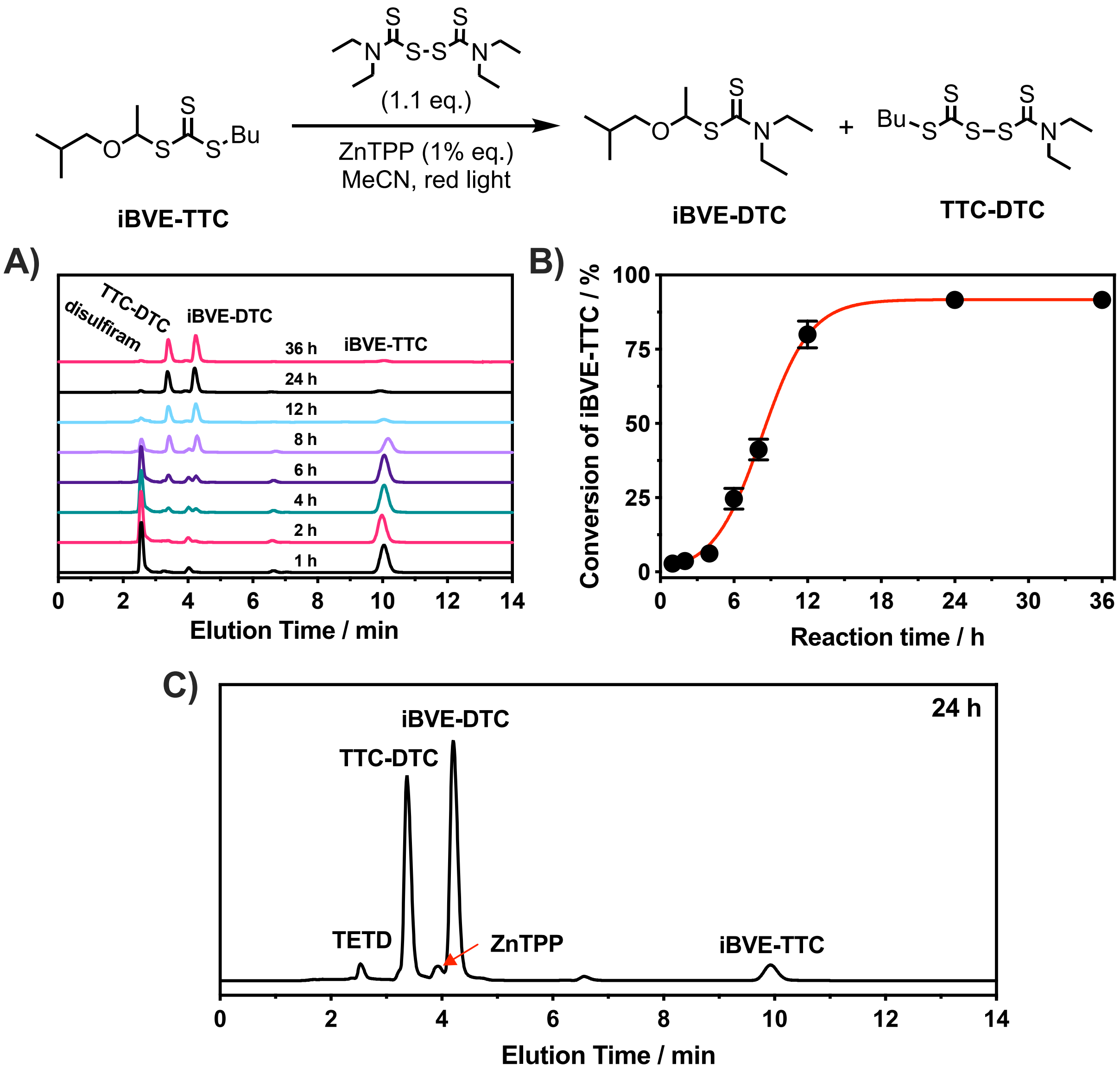

Supplementary Figure 2. Time-evolution of (a and c) HPLC traces (MeCN/H_2O 8/2 v/v; 300 nm absorbance) and (b) conversion recored for TTC-to-DTC transformation. Reaction was performed on 10 mmol scale using trithiocarbonate (iBVE-TTC, 1 eq.), TETD (1.1 eq.), and ZnTPP (1 mol %) in acetonitrile (MeCN) under red light irradiation (630 nm). The mean and error (± s.d.) were obtained from three independent experiments (n = 3).

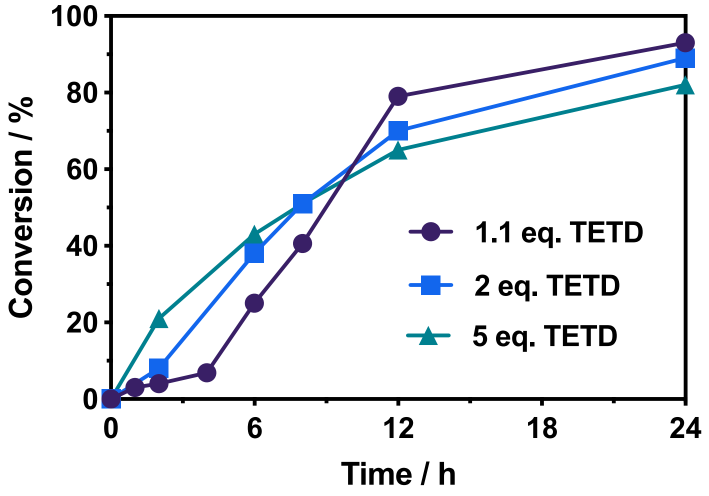

Supplementary Figure 3. Conversion for TTC-to-DTC transformation. Reaction was performed on 10 mmol scale using trithiocarbonate (iBVE-TTC, 1 eq.), TETD (1.1-5 eq.), and ZnTPP (1 mol %) in acetonitrile (MeCN) under red light irradiation (630 nm).

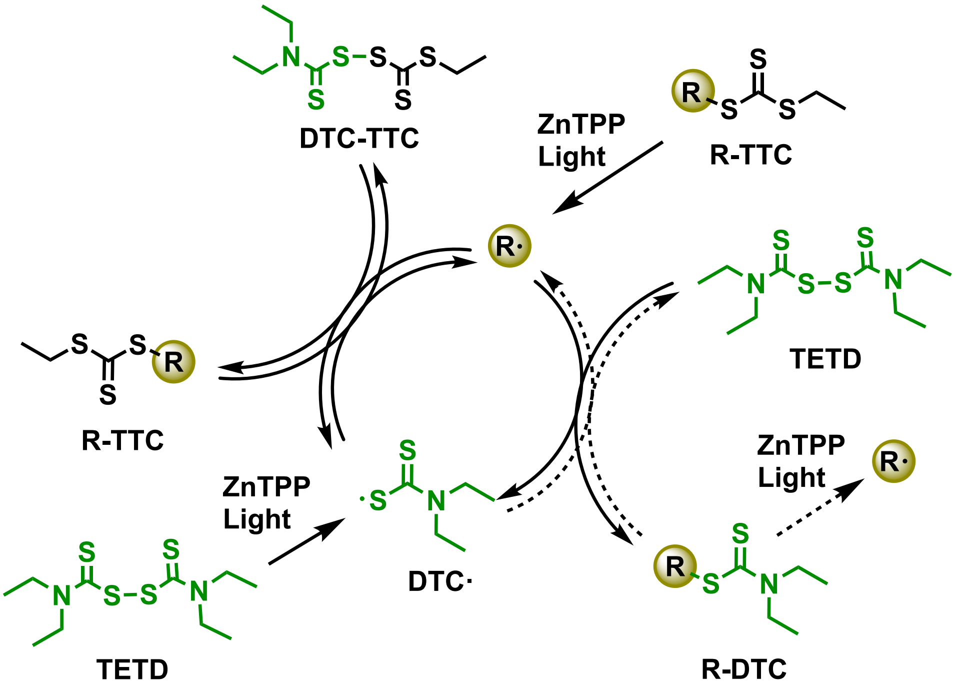

Supplementary Figure 4. proposed mechanism for TTC-to-DTC transformation in presence of ZnTPP under red light irradiation.

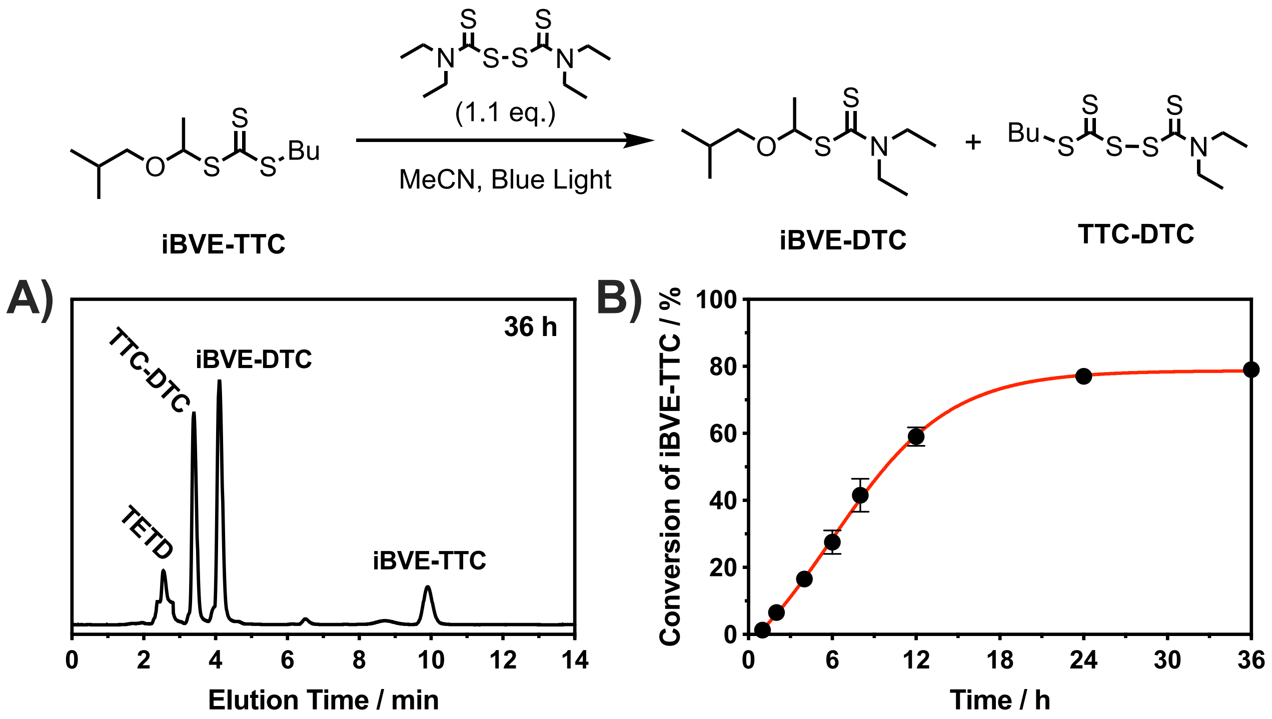

Supplementary Figure 5. Time-evolution of (a) HPLC trace (MeCN/H_2O 8/2 v/v; 300 nm absorbance) and (b) conversion recored for TTC-to-DTC transformation. Reaction was performed on 10 mmol scale using trithiocarbonate (iBVE-TTC, 1 eq.) and TETD (1.1 eq.) MeCN under blue light irradiation (460 nm). The mean and error (±s.d.) were obtained from three independent experiments (n = 2).

Synthesis of dimer trithiocarbonates via radical SUMI.

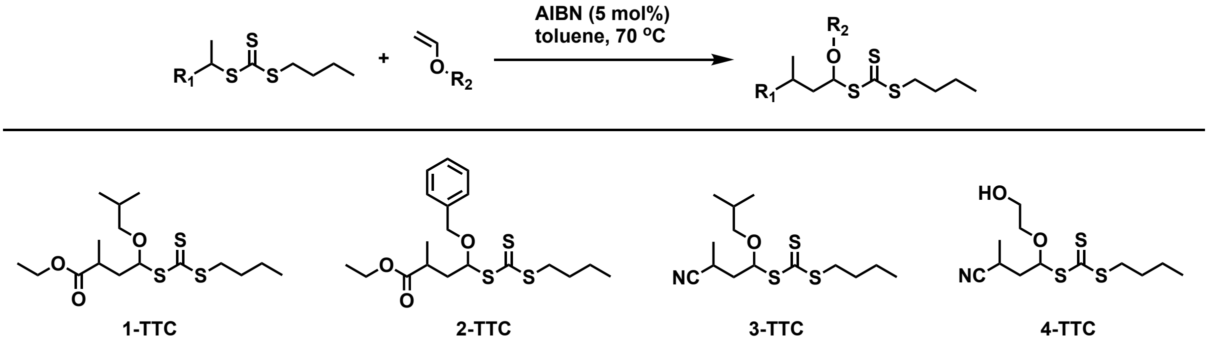

Supplementary Figure 6. Synthesis route for dimer trithiocarbonates (**1-TTC - 4-TTC**).

In a nitrogen filled glove box, trithiocarbonate (1 mmol), vinyl ether (2 mmol), AIBN (0.05 mmol), toluene (1 mL) were charged into an oven-dried 20 mL Schlenk tube equipped with a stir magneton. After being stirred for 24 h at 70 ºC, the crude product was purified by flash chromatography using petroleum ether/ethyl acetate as the eluent.

1-TTC: A yellow oil (yield: 85%). ^{1}H NMR ($CDCl_3$, δ, ppm): 6.0 (1H, -$CH_2C\underline{\mathbf{H}}$(-$OCH_2$-)S-), 4.1-4.2 (2H, $CH_3C\underline{\mathbf{H}}_2O$-), 3.2-3.5 (4H, -$SC\underline{\mathbf{H}}_2$-, $(CH_3)_2CHC\underline{\mathbf{H}}_2O$-), 2.3-2.8 (2H, >$CHC\underline{\mathbf{H}}_2CH$<), 1.9-2.2 (1H, -$CH_2C\underline{\mathbf{H}}(CH_3)COOCH_2$-), 1.7-1.9 (1H, -$C\underline{\mathbf{H}}(CH_3)_2$-), 1.6-1.7 (2H, -$C\underline{\mathbf{H}}_2$-), 1.4-1.5 (2H, -$C\underline{\mathbf{H}}_2CH_3$), 1.2-1.3 (6H, -$CH_2CH(C\underline{\mathbf{H}}_3)COOCH_2$-, $C\underline{\mathbf{H}}_3CH_2OOC$-), 0.9 (9H, -$CH_2C\underline{\mathbf{H}}_3$, $(C\underline{\mathbf{H}}_3)_2CH$-). ^{13}C NMR ($CDCl_3$, δ, ppm): 175, 91, 60, 40, 41, 37, 30, 28, 22, 19, 18, 17, 14, 13. ESI-MS: m/z calc. for $C_{16}H_{30}O_3S_3Na$: 389.06 $[M+Na]^+$; found: 389.06.

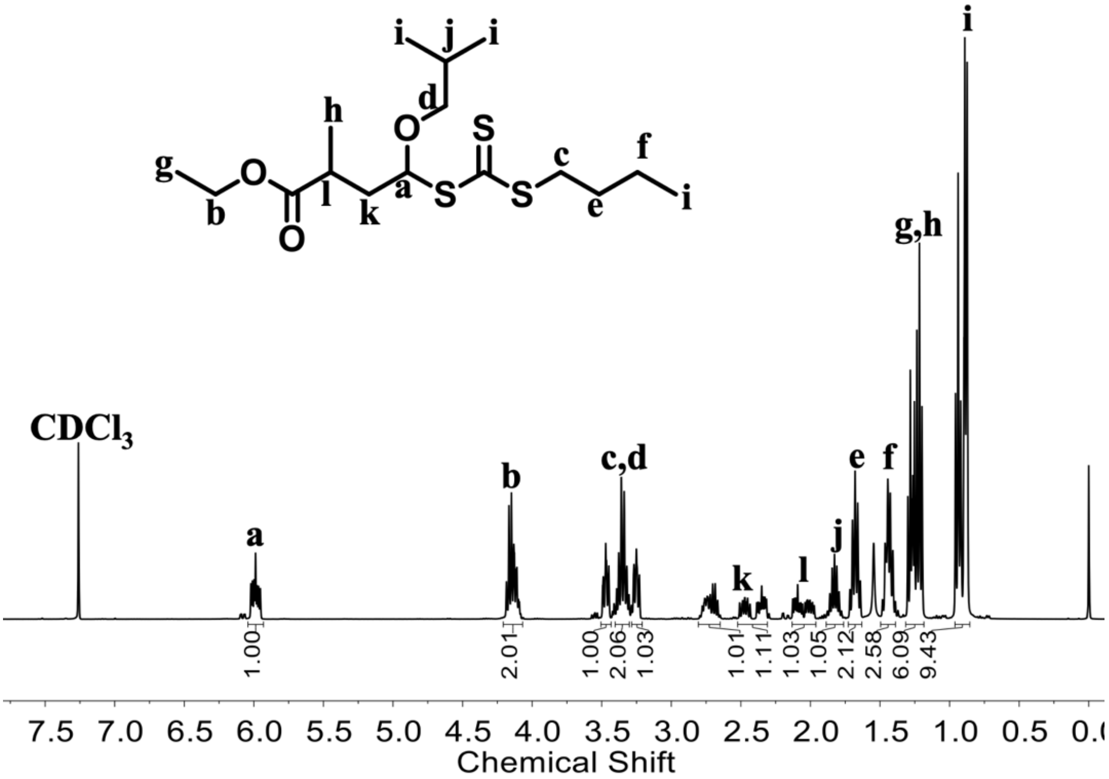

Supplementary Figure 7. ^{1}H NMR spectrum for **1-TTC** in $CDCl_3$.

2-TTC: A yellow oil (yield: 97%). ^{1}H NMR ($CDCl_3$, δ, ppm): 7.3-7.5 (5H, aromatic proton), 6.1-6.2 (1H, $-CH_2C\underline{\mathbf{H}}(-OCH_2-)S-$), 4.6-4.8 (2H, $-OC\underline{\mathbf{H}}_2$-Ph), 4.0-4.2 (2H, $CH_3C\underline{\mathbf{H}}_2OOC-$), 3.4-3.5 (2H, $-SC\underline{\mathbf{H}}_2CH_2CH_2CH_3$), 2.0-2.8 (3H, $-OOCC\underline{\mathbf{H}}(-CH_3)C\underline{\mathbf{H}}_2CH<$), 1.7-1.8 and 1.4-1.5 (2H, $-SC\underline{\mathbf{H}}_2C\underline{\mathbf{H}}_2CH_2CH_3$), 1.2-1.3 (6H, $C\underline{\mathbf{H}}_3CH_2OOCCH(-C\underline{\mathbf{H}}_3)-$), 0.9 (3H, $-SCH_2CH_2CH_2C\underline{\mathbf{H}}_3$). ^{13}C NMR ($CDCl_3$, δ, ppm): 175, 142, 136, 129, 128, 127, 90, 72, 66, 65, 60, 40, 36, 30, 23, 18, 17, 14, 13. ESI-MS: m/z calc. for $C_{19}H_{29}O_3S_3$: 401.1. $[M+H]^+$; found: 401.1.

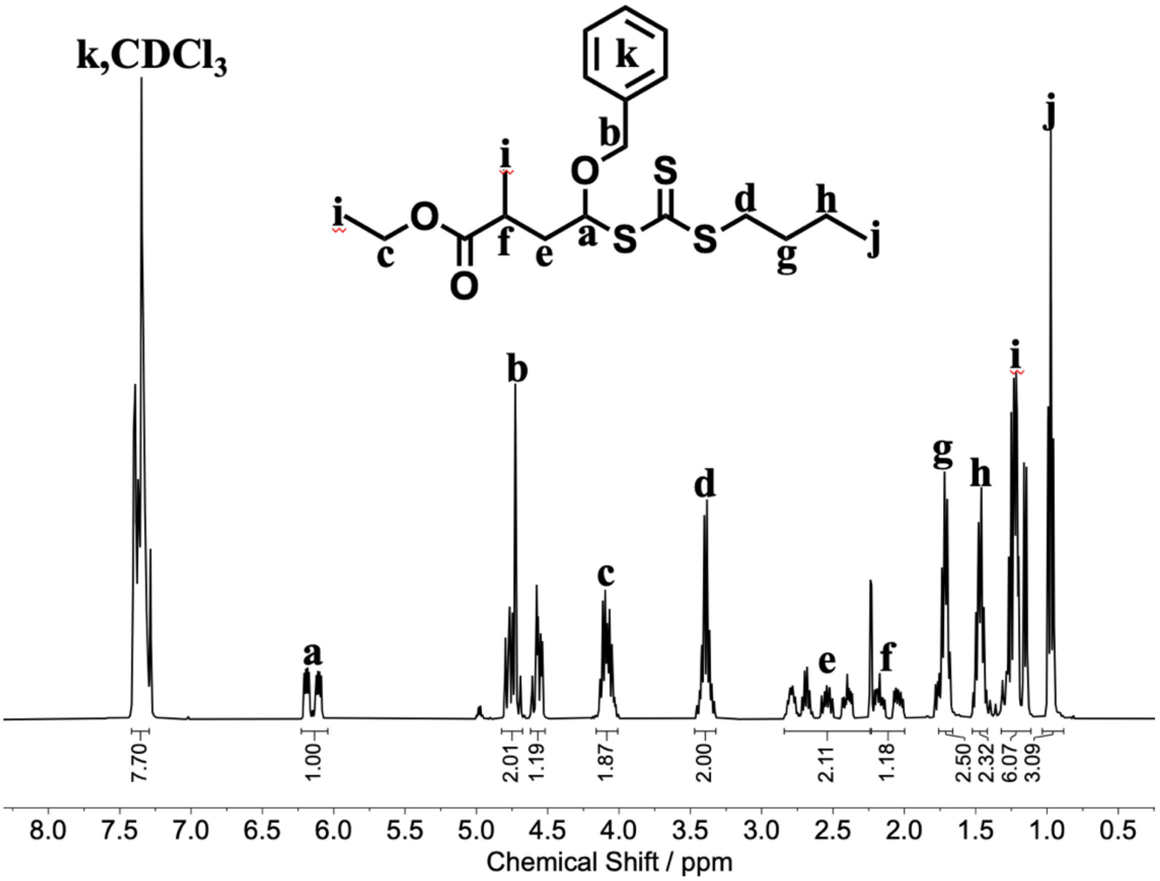

Supplementary Figure 8. ^{1}H NMR spectrum for **2-TTC** in $CDCl_3$.

3-TTC: A yellow oil (yield: 88%). ^{1}H NMR ($CDCl_3$, δ, ppm): 5.9-6.1 (1H, -CH_2C**H**(-OCH_2-)S-), 3.2-3.6 (4H, $(CH_3)_2CHC$**H**$_2$O-, -SC**H**$_2$-), 2.8-3.0 (1H, NCC**H**(CH_3)CH_2-), 2.1-2.5 (2H, >CHC**H**$_2$CH<), 1.9 (1H, $(CH_3)_2C$**H**-), 1.7 (2H, -C**H**$_2$-), 1.3-1.5 (5H, -C**H**$_2CH_3$, NCCH(C**H**$_3$)CH_2-), 0.9 (9H, -CH_2C**H**$_3$, (C**H**$_3$)$_2$CH-).^{13}C NMR ($CDCl_3$, δ, ppm): 122, 90, 40, 41, 36, 30, 28, 23, 22, 19, 18, 17, 13. ESI-MS: m/z calc. for $C_{14}H_{25}NOS_3Na$: 342.08 $[M+Na]^+$; found: 342.08.

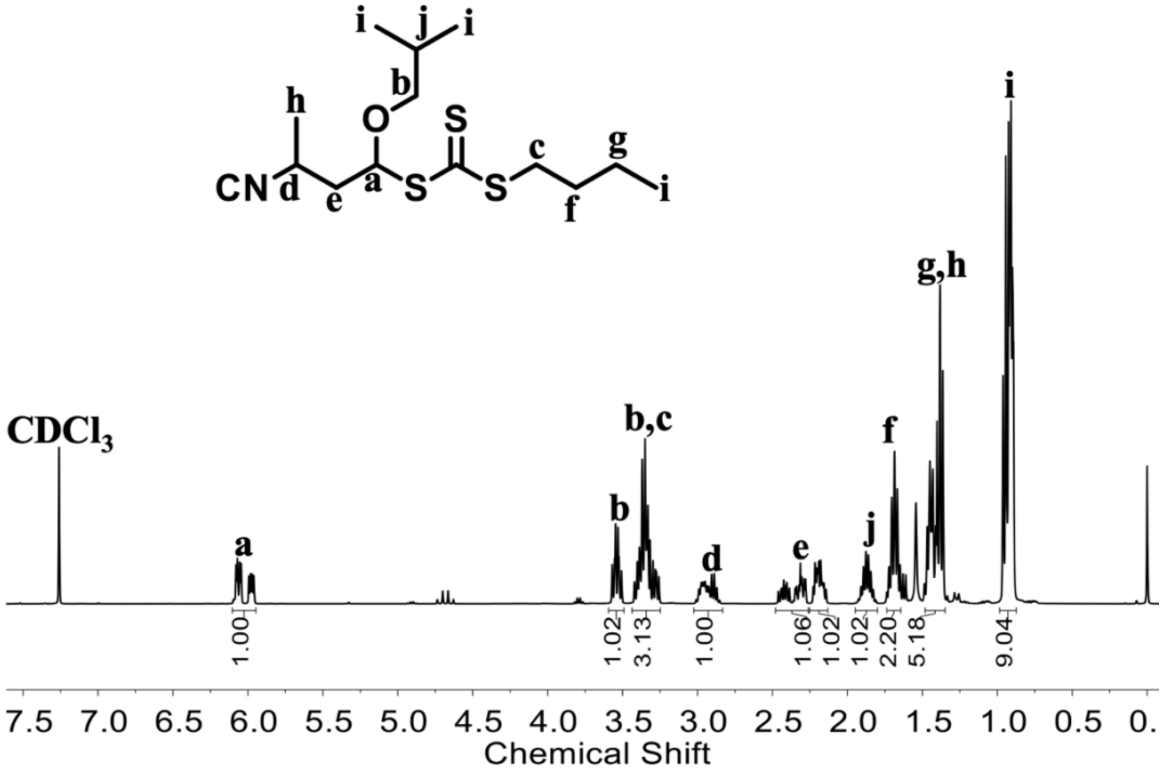

Supplementary Figure 9. ^{1}H NMR spectrum for **3-TTC** in $CDCl_3$.

4-TTC: A yellow oil (yield: 77%). ^{1}H NMR ($CDCl_3$, δ, ppm): 6.0-6.2 (1H, -CH_2C**H**(-OCH_2-)S-), 3.6-4.0 (4H, -OC**H**$_2$C**H**$_2$OH), 3.3-3.5 (2H, -SC**H**$_2$-), 2.8-3.0 (1H, NCC**H**(-CH_3)CH_2-), 2.1-2.5 (2H, >CHC**H**$_2$CH<), 1.7 (2H, -C**H**$_2$-), 1.3-1.5 (5H, -C**H**$_2$$CH_3$, NCCH(-C**H**$_3$)$CH_2$-), 0.9 (3H, -$CH_2$C**H**$_3$). ^{13}C NMR ($CDCl_3$, δ, ppm):123, 122, 90, 89, 71, 61, 40, 37, 30, 23, 22, 18, 14. ESI-MS: m/z calc. for $C_{12}H_{21}NO_2S_3Na$: 330.03 [M+Na]+; found: 330.03.

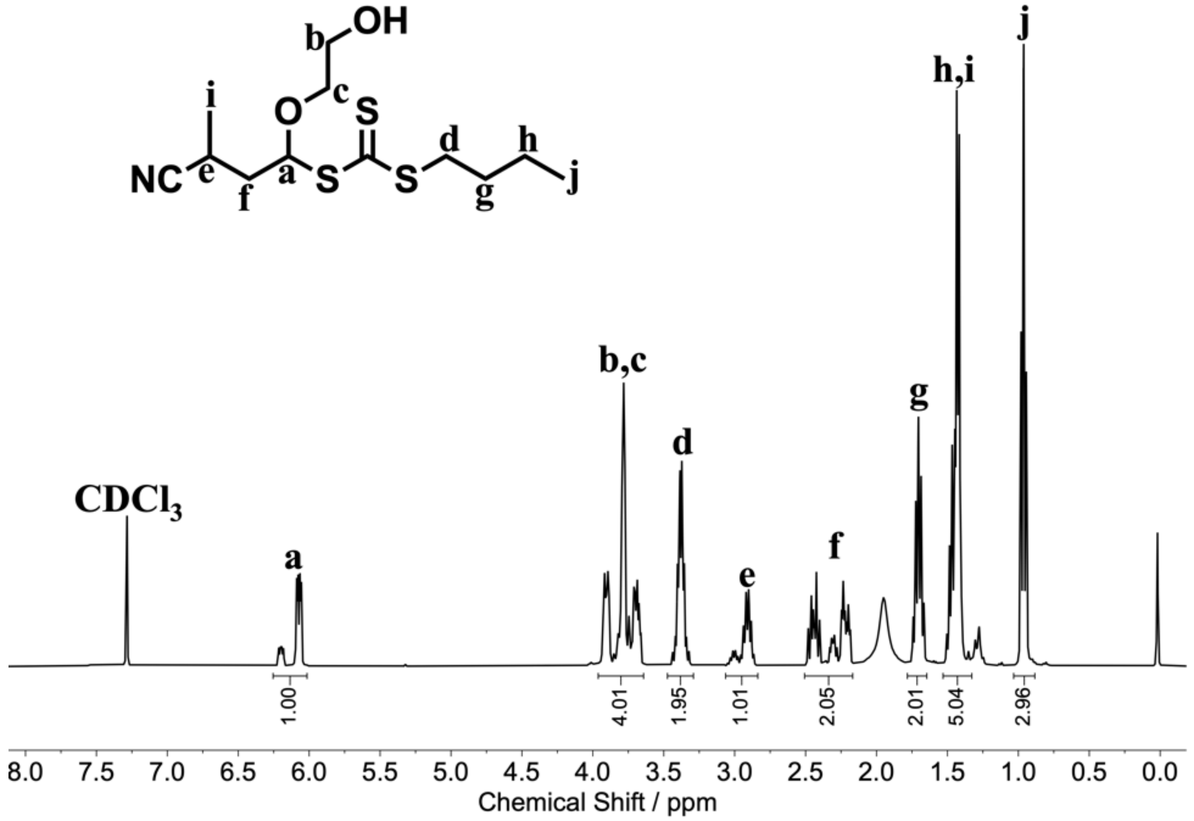

Supplementary Figure 10. ^{1}H NMR spectrum for **4-TTC** in $CDCl_3$.

Synthesis of trimer trithiocarbonates via two-step radical SUMI.

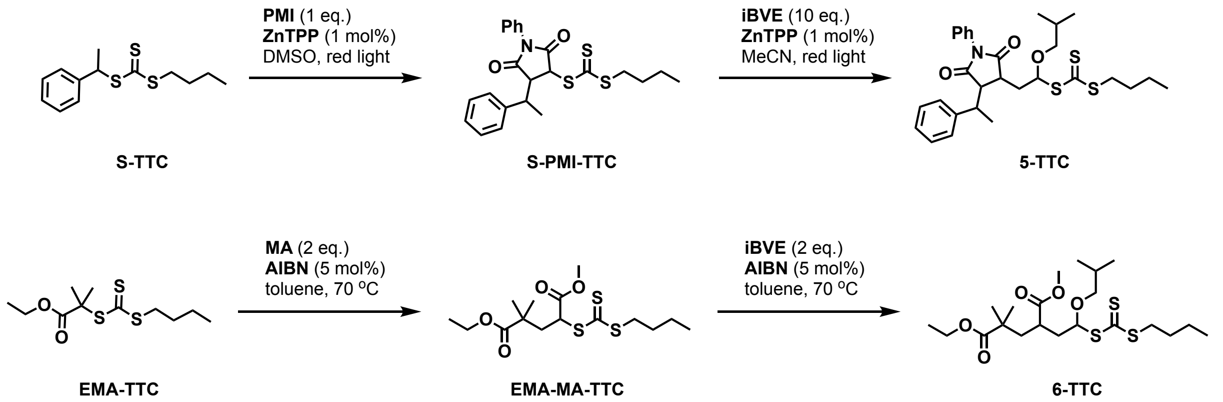

Supplementary Figure 11. Synthesis route for trimer trithiocarbonates (**5-TTC** and **6-TTC**).

Synthesis of S-PMI-TTC: It was synthesized according to the literature.[11]

Synthesis of 5-TTC: In a nitrogen filled glove box, **S-PMI-TTC** (1 equiv), **iBVE** (10 equiv), ZnTPP (1 mol %), and MeCN were charged into an oven-dried 20 mL Schlenk tube equipped with a stir magneton. The mixture was stirred under red light (630 nm) irradiation over a period of 24 h at 25 ºC. The crude product was further purified by flash chromatography using PETROLEUM ETHER/ETHYL ACETATE as the eluent, affording **5-TTC** as a yellow solid (yield: 92%).^{1}H NMR ($CDCl_3$, δ, ppm): 6.8-7.5 (10H, aromatic proton), 5.8-6.2 (1H, -C**H**(O-)S-), 3.2-3.8 (5H, -SC**H**$_2$-, -OC**H**$_2$-, and Ph-C**H**<), 2.9-3.1 (2H, >C**H**CO-), 2.1-2.5 (2H, >CHC**H**$_2$CH<), 1.7-1.9 (3H, -CH_3), 1.4-1.6 (5H, -C**H**(CH_3)$_2$ and -C**H**$_2$C**H**$_2$-), 0.8-1.0 (9H, -C**H**$_3$ and -CH(C**H**$_3$)$_2$). ^{13}C NMR ($CDCl_3$, δ, ppm): 178, 132, 129, 128, 127, 126, 110, 90, 89, 52, 50, 41, 40, 38, 36, 30, 29, 22, 19 16, 14. ESI-MS: m/z calc. for $C_{29}H_{37}NO_3S_3Na$: 566.18 $[M+Na]^+$; found: 566.18.

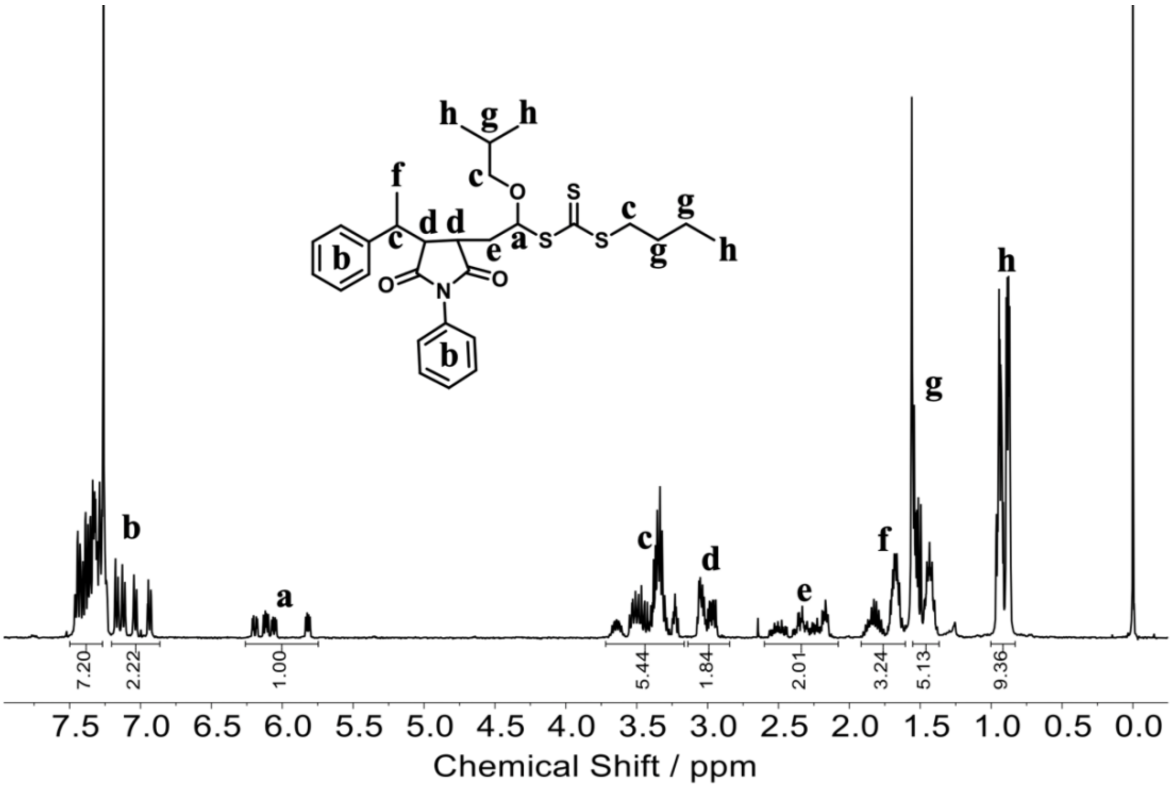

Supplementary Figure 12. ^{1}H NMR spectrum for **5-TTC** in $CDCl_3$.

Synthesis of EMA-MA-TTC: In a nitrogen filled glove box, EMA-TTC (10 mmol), MA (20 mmol), AIBN (0.5 mmol), toluene (10 mL) were charged into an oven-dried 50 mL Schlenk tube equipped with a stir magneton. After being stirred for 24 h at 70 °C, the crude product was purified by flash chromatography affording **EMA-MA-TTC** as a yellow solid (yield: 87%). ^{1}H NMR ($CDCl_3$, δ, ppm): 4.9-5.0 (1H, $-CH_2C\underline{\mathbf{H}}(COOCH_3)S-$), 4.1-4.2 (2H, $CH_3C\underline{\mathbf{H}}_2OOC-$), 3.7-3.8 (3H, $-CH(COOC\underline{\mathbf{H}}_3)S-$), 3.4-3.5 (2H, $-SC\underline{\mathbf{H}}_2\ CH_2\ CH_2\ CH_3$), 2.1-2.5 (2H, $-C(CH_3)_2C\underline{\mathbf{H}}_2\ CH(COOCH_3)S-$), 1.7 (2H, $-SCH_2\ C\underline{\mathbf{H}}_2\ CH_2\ CH_3$), 1.4 ($-SCH_2\ CH_2\ C\underline{\mathbf{H}}_2\ CH_3$), 1.2-1.3 (9H, $C\underline{\mathbf{H}}_3CH_2-$, $-C(C\underline{\mathbf{H}}_3)_2CH_2\ C<$), 0.9-1.0 (3H, $-SCH_2\ CH_2\ CH_2\ C\underline{\mathbf{H}}_3$). ^{13}C NMR ($CDCl_3$, δ, ppm): 176, 171, 61, 53, 50, 42, 41, 37, 30, 27, 25, 22, 14. ESI-MS: m/z calc. for $C_{15}H_{27}O_4S_3$: 367.1 $[M+Na]^+$; found: 367.1.

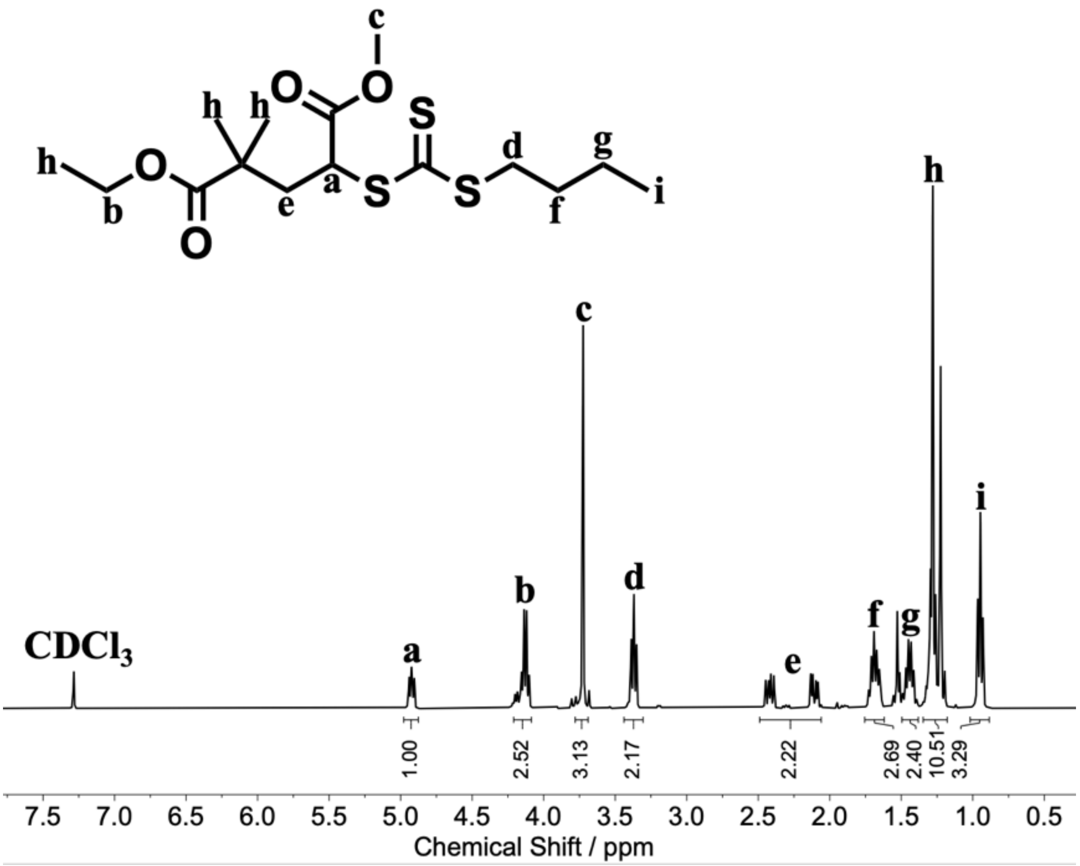

Supplementary Figure 13. ^{1}H NMR spectrum for **EMA-MA-TTC** in $CDCl_3$.

Synthesis of 6-TTC: In a nitrogen filled glove box, **EMA-MA-TTC** (10 mmol), iBVE (20 mmol), AIBN (0.5 mmol), toluene (10 mL) were charged into an oven-dried 50 mL Schlenk tube equipped with a stir magneton. After being stirred for 24 h at 70 °C, the crude product was purified by flash chromatography affording **6-TTC** as a yellow solid (yield: 87%). ^{1}H NMR ($CDCl_3$, δ, ppm): 5.8-6.0 (1H, $-CH_2C\underline{\mathbf{H}}(OCH_2-)S-$), 4.1-4.2 (2H, $CH_3C\underline{\mathbf{H}}_2OOC-$), 3.6-3.7 (3H, $>CH(COOC\underline{\mathbf{H}}_3)-$), 3.3-3.5 (4H, $CH(CH_3)_2C\underline{\mathbf{H}}_2O-$, $-SC\underline{\mathbf{H}}_2\ CH_2\ CH_2\ CH_3$), 2.0-3.0 (4H, $-OOCC(CH_3)_2C\underline{\mathbf{H}}_2\ CH\ -$, $>CHC\underline{\mathbf{H}}_2CH<$), 1.6-1.7 (4H, $-C\underline{\mathbf{H}}(COOCH_3)-$, $C\underline{\mathbf{H}}(CH_3)_2CH_2O-$, $-SCH_2\ C\underline{\mathbf{H}}_2\ CH_2\ CH_3$), 1.4-1.5 ($-SCH_2\ CH_2\ C\underline{\mathbf{H}}_2\ CH_3$), 1.2-1.3 (9H, $C\underline{\mathbf{H}}_3CH_2-$, $-C(C\underline{\mathbf{H}}_3)_2CH_2\ C<$), 0.9-1.0 (9H, $CH(C\underline{\mathbf{H}}_3)_2CH_2O-$, $-SCH_2\ CH_2\ CH_2\ C\underline{\mathbf{H}}_3$). ^{13}C NMR ($CDCl_3$, δ, ppm): 200, 177, 175, 92, 91, 70, 61, 51, 47, 43, 42, 40, 36, 30, 29, 25, 22, 20, 14. ESI-MS: m/z calc. for $C_{21}H_{38}O_5S_3Na$: 489.7 $[M+Na]^+$; found: 489.7.

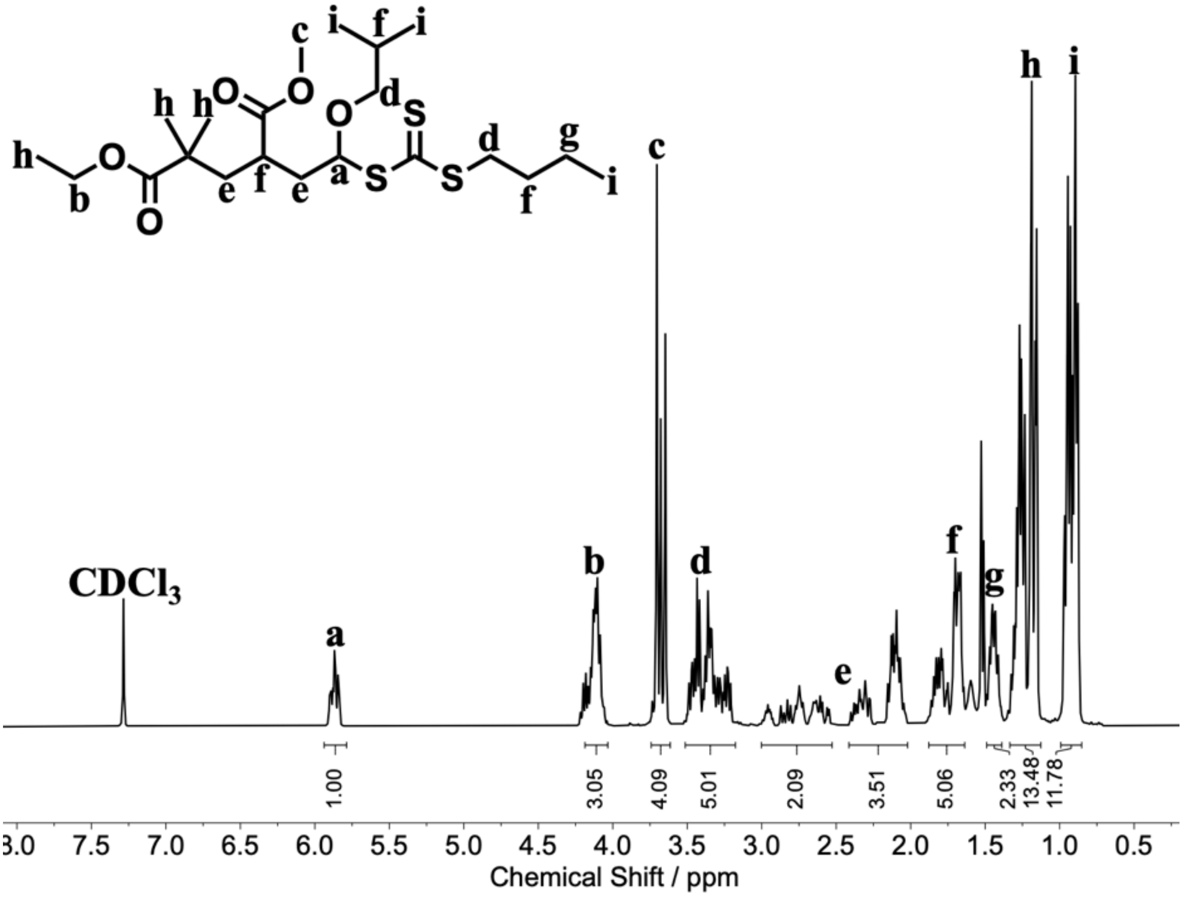

Supplementary Figure 14. ^{1}H NMR spectrum for **6-TTC** in $CDCl_3$.

Synthesis of trimer trithiocarbonates via three-step radical SUMI.

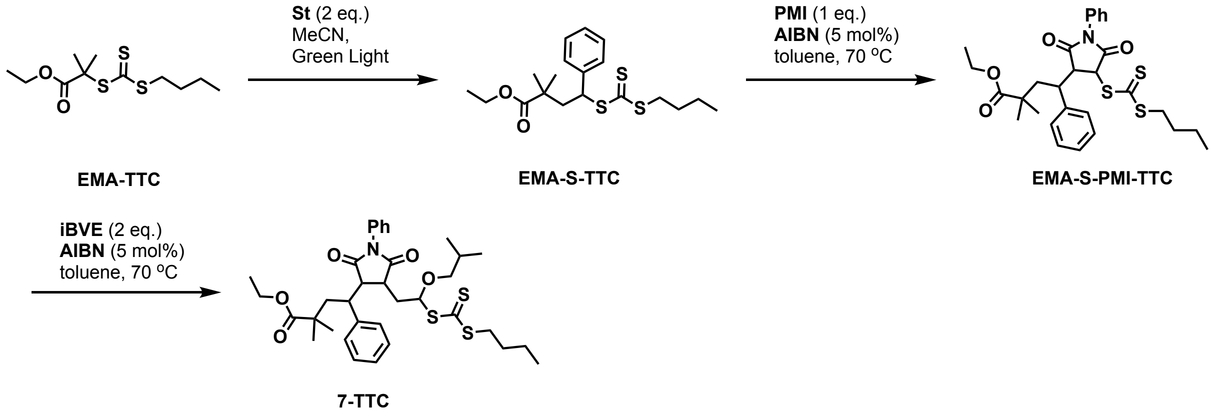

Supplementary Figure 15. Synthesis route for trimer trithiocarbonate, **7-TTC**.

Synthesis of EMA-S-TTC: In a nitrogen filled glove box, EMA-TTC (10 mmol), styrene (20 mmol), MeCN (10 mL) were charged into an oven-dried 50 mL Schlenk tube equipped with a stir magneton. After being stirred under green light (530 nm) for 24 h, the crude product was purified by flash chromatography as the eluent affording **EMA-S-TTC** as a yellow solid (yield: 82%). ^{1}H NMR ($CDCl_3$, δ, ppm): 7.2-7.4 (5H, aromatic proton), 5.3-5.4 (1H, -CH_2C<u>**H**</u>(-Ph)S-), 3.6-3.8 (2H, CH_3C<u>**H**</u>$_2$OOC-), 3.3-3.4 (2H, -SC<u>**H**</u>$_2$ CH_2 CH_2 CH_3), 2.3-2.5 (2H, -OOCC(CH_3)$_2$C<u>**H**</u>$_2$CH(-Ph)-), 1.7 and 1.4 (4H, -SCH_2 C<u>**H**</u>$_2$ C<u>**H**</u>$_2$ CH_3), 1.2-1.3 (9H, -OOCC(C<u>**H**</u>$_3$)$_2$$CH_2$-, C<u>**H**</u>$_3$$CH_2$OOCC-), 0.9 (3H, -$SCH_2$ CH_2 CH_2 C<u>**H**</u>$_3$). ^{13}C NMR ($CDCl_3$, δ, ppm): 176, 140, 129, 60, 52, 45, 42, 36, 30, 26, 25, 22, 14. ESI-MS: m/z calc. for $C_{19}H_{29}O_2S_3$: 385.1. $[M+H]^+$; found: 385.1.

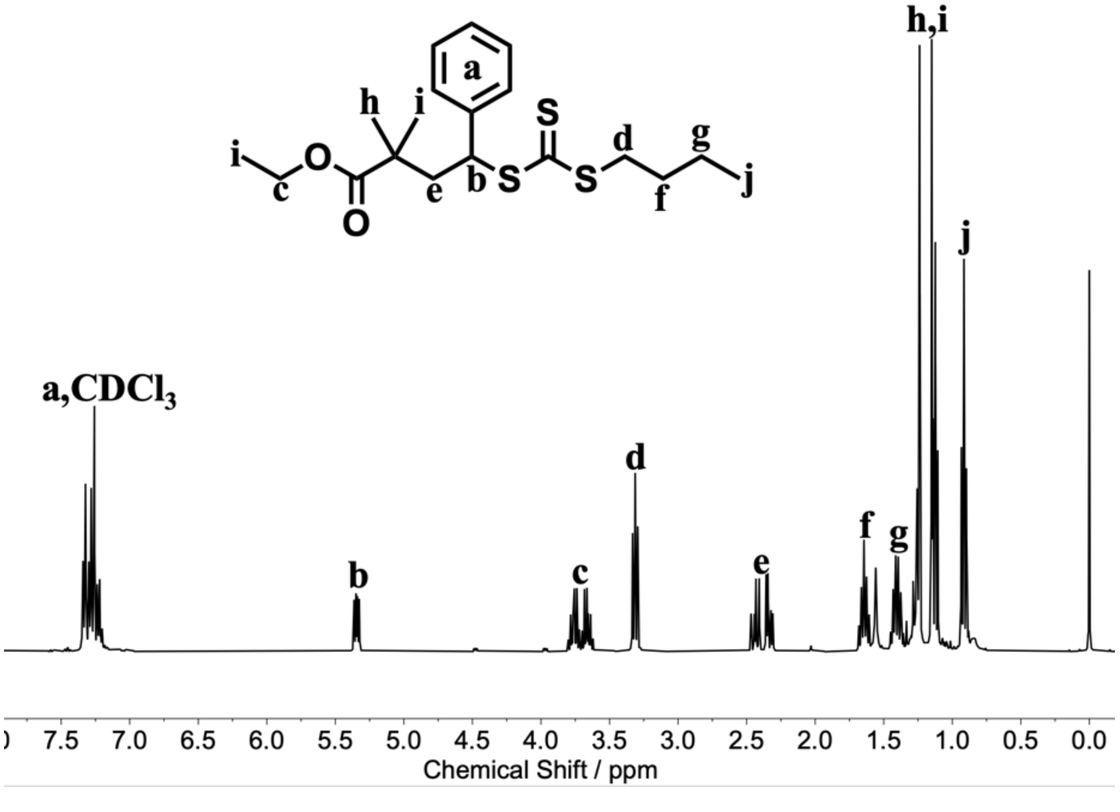

Supplementary Figure 16. ^{1}H NMR spectrum for **EMA-S-TTC** in $CDCl_3$.

Synthesis of EMA-S-PMI-TTC: In a nitrogen filled glove box, **EMA-S-TTC** (10 mmol), PMI (10 mmol), AIBN (0.5 mmol), toluene (10 mL) were charged into an oven-dried 50 mL Schlenk tube equipped with a stir magneton. After being stirred for 24 h at 70 °C, the crude product was purified by flash chromatography as the eluent affording **EMA-S-PMI-TTC** as a yellow solid (yield: 67%). ^{1}H NMR ($CDCl_3$, δ, ppm): 7.2-7.5 (10H, aromatic proton), 4.6 (1H, >C**H**S-), 3.5-4.0 (4H, $CH_3C\underline{\mathbf{H}}_2OOC$-, -$CH_2C\underline{\mathbf{H}}$(-Ph)C**H**<), 3.4 (2H, -$SC\underline{\mathbf{H}}_2$ CH_2 CH_2 CH_3), 2.3 and 2.7-2.8 (2H, -$C\underline{\mathbf{H}}_2CH$(-Ph)-), 1.7-1.8 and 1.5 (4H, -SCH_2 $C\underline{\mathbf{H}}_2$ $C\underline{\mathbf{H}}_2$ CH_3), 1.1-1.3 (9H, -$OOCC(C\underline{\mathbf{H}}_3)_2CH_2$-, $C\underline{\mathbf{H}}_3CH_2OOCC$-), 1.0 (3H, -$SCH_2$ CH_2 CH_2 $C\underline{\mathbf{H}}_3$). ^{13}C NMR ($CDCl_3$, δ, ppm): 177, 175, 172, 167, 138, 132, 130, 128, 60, 53, 48, 43, 37, 30, 27, 25, 22, 15. ESI-MS: m/z calc. for $C_{29}H_{33}NO_4S_3Na$: 578.8. $[M+Na]^+$; found: 578.8.

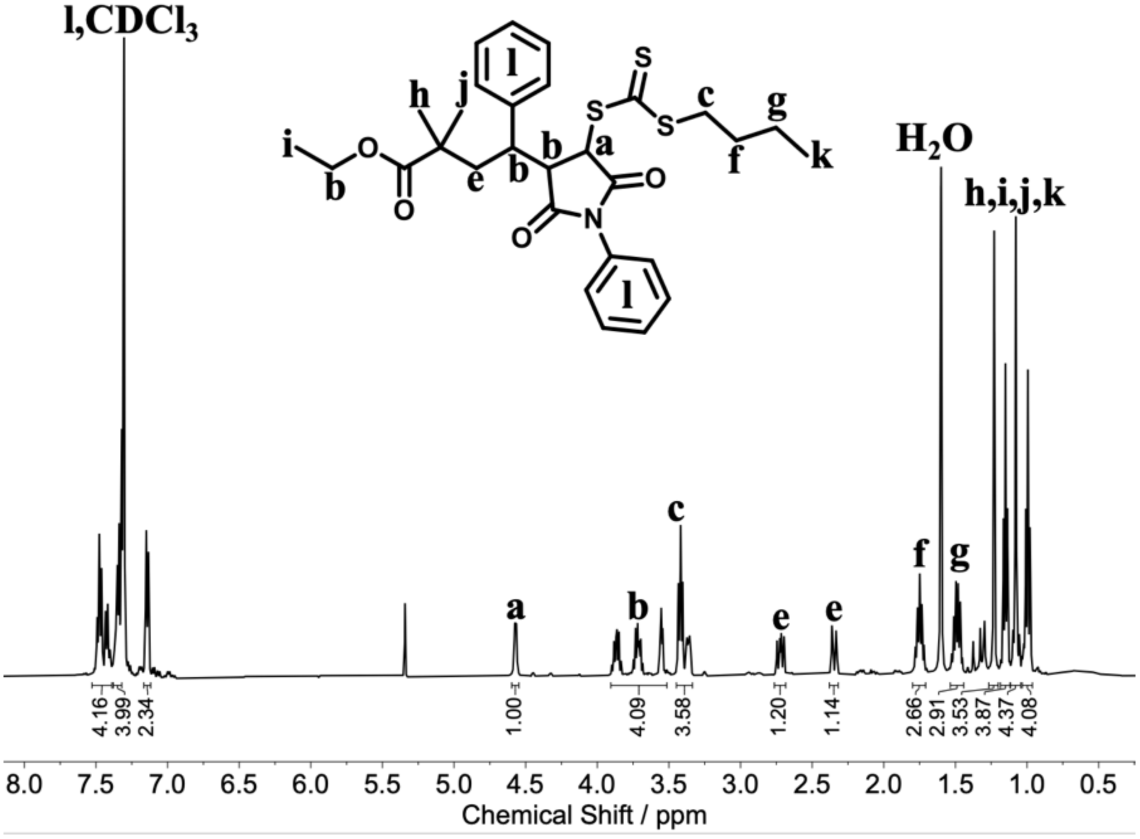

Supplementary Figure 17. ^{1}H NMR spectrum for **EMA-S-PMA-TTC** in $CDCl_3$.

Synthesis of 7-TTC: In a nitrogen filled glove box, **EMA-S-PMA-TTC** (10 mmol), iBVE (20 mmol), AIBN (0.5 mmol), toluene (10 mL) were charged into an oven-dried 50 mL Schlenk tube equipped with a stir magneton. After being stirred for 24 h at 70 °C, the crude product was purified by flash chromatography as the eluent affording **7-TTC** as a yellow solid (yield: 52%). ^{1}H NMR ($CDCl_3$, δ, ppm): 7.3-7.5 and 6.7-7.0 (10H, aromatic proton), 6.2 (1H, -$CH_2C\underline{\mathbf{H}}$(-O-)S-), 3.0-3.8 (9H, $CH_3C\underline{\mathbf{H}}_2OOC$-, -$CH_2C\underline{\mathbf{H}}$(-Ph) -, >$C\underline{\mathbf{H}}C\underline{\mathbf{H}}$<, -$OC\underline{\mathbf{H}}_2CH(CH_3)_2$, -$SC\underline{\mathbf{H}}_2$ CH_2 CH_2 CH_3), 2.0-2.6 (4H, -$C\underline{\mathbf{H}}_2$CH(-Ph)-, >$CHC\underline{\mathbf{H}}_2$CH(-O-)S-), 1.8-2.0 (1H, -$OCH_2C\underline{\mathbf{H}}(CH_3)_2$), 1.7 and 1.4-1.5 (4H, -SCH_2 $C\underline{\mathbf{H}}_2$ $C\underline{\mathbf{H}}_2$ CH_3), 1.2-1.4 (6H, -$OOCC(C\underline{\mathbf{H}}_3)_2CH_2$-), 0.9-1.2 (12H, $C\underline{\mathbf{H}}_3CH_2OOCC$-, -$SCH_2$ CH_2 CH_2 $C\underline{\mathbf{H}}_3$, -$OCH_2CH(C\underline{\mathbf{H}}_3)_2$). ^{13}C NMR ($CDCl_3$, δ, ppm): 176, 175, 129, 128, 90, 60, 54, 53, 44, 43, 40, 36, 30, 29, 27, 25. ESI-MS: m/z calc. for $C_{35}H_{45}NO_5S_3Na$: 678.9. $[M+Na]^+$; found:678.9.

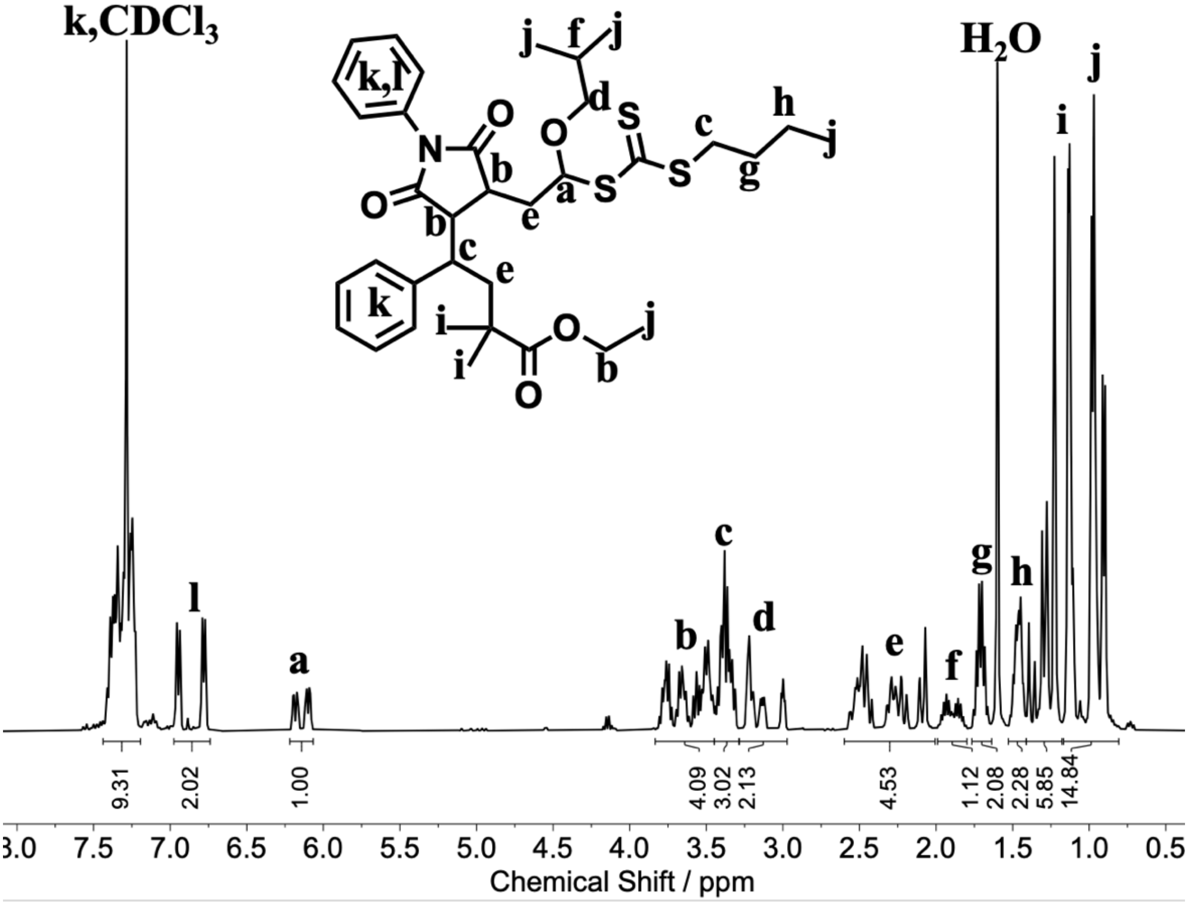

Supplementary Figure 18. ^{1}H NMR spectrum for **7-TTC** in $CDCl_3$.

Transfor from trithiocarbonate to dithiocarbamate via radical process

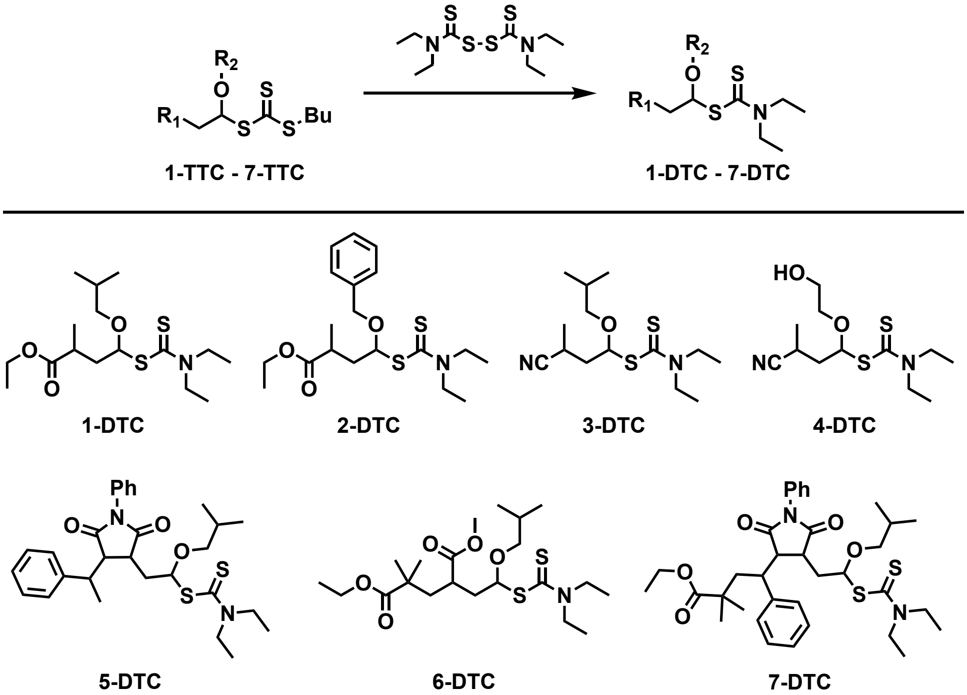

Supplementary Figure 19. Synthesis route for dithiocarbamates (**1-DTC** – **7-DTC**) from trithiocarbonates (**1-TTC** - **7-TTC**).

In a nitrogen filled glove box, trithiocarbonate (1 equiv), TETD (1.1 equiv), ZnTPP (0.01 equiv), MeCN (2.2 mL) were charged into an oven-dried 20 mL Schlenk tube equipped with a stir magneton. The mixture was stirred under red light irradiation (630 nm) at room temperature for 24 h. The crude product was purified by flash chromatography using petroleum ether/ethyl acetate (PE/EA) as the eluent.

1-DTC: A yellow solid (yield: 87%). ^{1}H NMR ($CDCl_3$, δ, ppm): ^{1}H NMR ($CDCl_3$, δ, ppm): 5.9-6.0 (1H, -CH2C**H**(-OCH2-)S-), 3.9-4.3 (4H, -N(C**H**2CH3)2), 3.7-3.8 (2H, CH3C**H**2O-), 3.2-3.6 (2H, (CH3)2CHC**H**2O-), 2.4-2.9 (2H, >CHC**H**2CH<), 2.1 (1H, -CH2C**H**(CH3)COOCH2-), 1.8-1.9 (1H, -C**H**(CH3)2), 1.2-1.4 (12H, -CH2CH(C**H**3)COOCH2-, C**H**3CH2O- and -N(CH2C**H**3)2), 0.9 (6H, -CH(C**H**3)2).^{13}C NMR ($CDCl_3$, δ, ppm): 194, 176, 93, 76, 60, 49, 47, 41, 37, 29, 20, 18, 17, 14, 13, 12. ESI-MS: m/z calc. for $C_{16}H_{31}NO_3S_2Na$: 372.25 $[M+Na]^+$; found: 372.25.

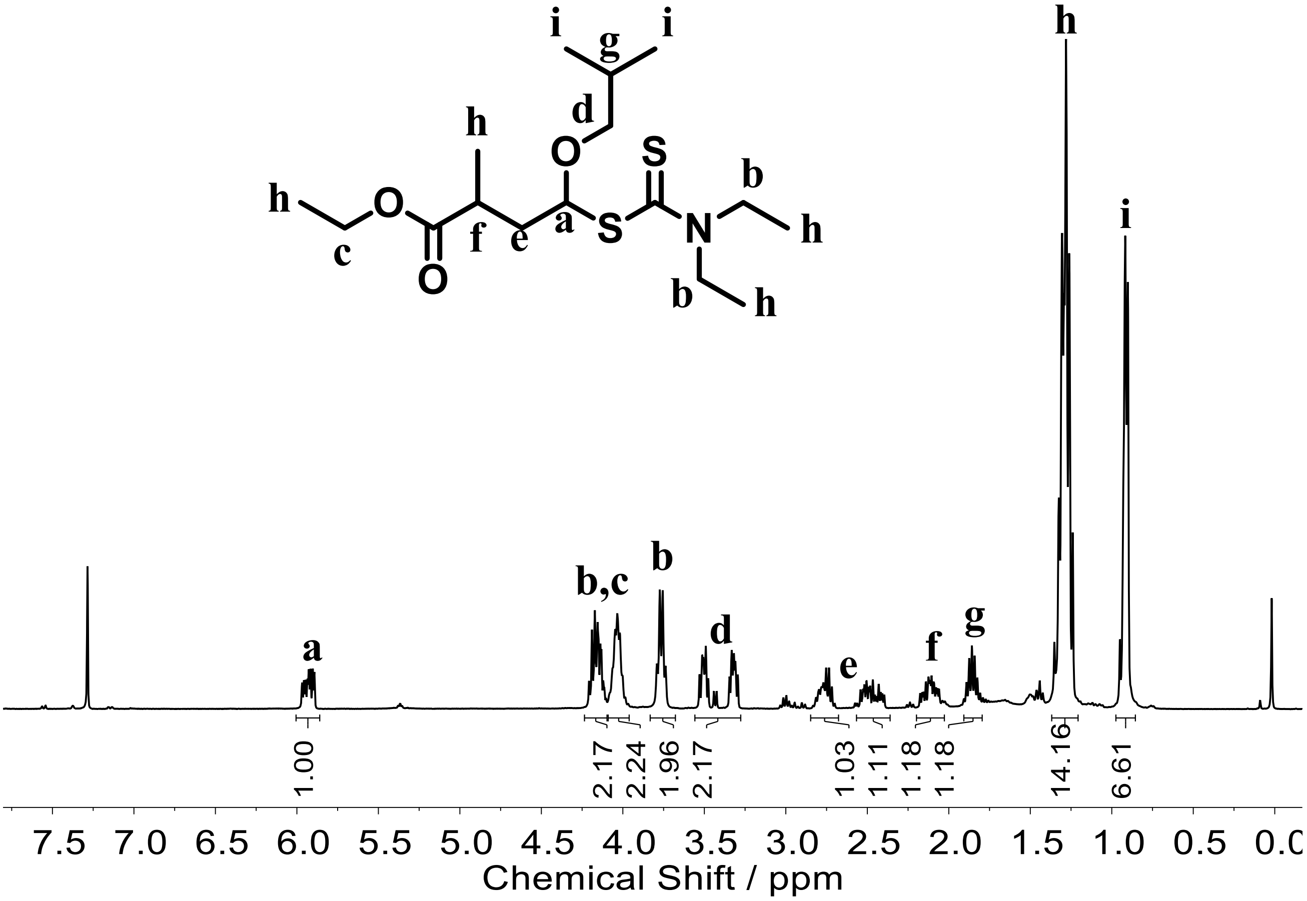

Supplementary Figure 20. ^{1}H NMR spectrum for **1-DTC** in $CDCl_3$.

2-DTC: A yellow solid (yield: 88%).^{1}H NMR ($CDCl_3$, δ, ppm): 7.3-7.5 (5H, aromatic proton), 6.1-6.2 (1H, $-CH_2C\underline{\mathbf{H}}(-OCH_2-)S-$), 4.6-4.7 (2H, $-OC\underline{\mathbf{H}}_2Ph$), 4.1-4.2 and 3.7-3.8 (6H, $CH_3C\underline{\mathbf{H}}_2OOC-$, $-N(C\underline{\mathbf{H}}_2CH_3)_2$), 2.5-3.1 (2H, $>CHC\underline{\mathbf{H}}_2CH<$), 2.1-2.2 (1H, $-OOCC\underline{\mathbf{H}}(-CH_3)-$), 1.4-1.6 (6H, $C\underline{\mathbf{H}}_3CH_2OOCCH(-C\underline{\mathbf{H}}_3)-$), 1.3-1.4 (6H, $-N(CH_2C\underline{\mathbf{H}}_3)_2$).^{13}C NMR ($CDCl_3$, δ, ppm): 195, 194, 176, 154, 138, 128, 94, 71, 60, 52, 49, 48, 47, 41, 37, 17, 14, 13, 12, 11. ESI-MS: m/z calc. for $C_{19}H_{29}NO_3S_2Na$: 406.6. $[M+Na]^+$; found: 406.6.

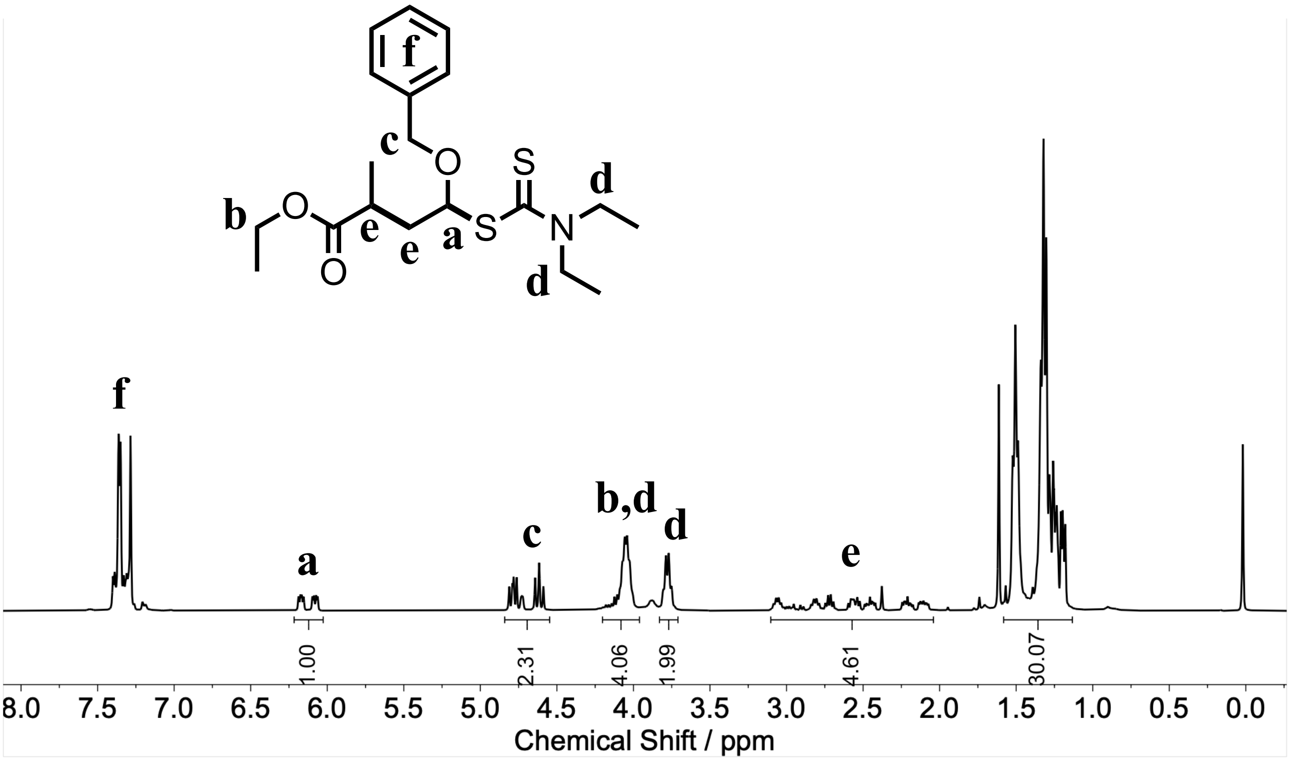

Supplementary Figure 21. ^{1}H NMR spectrum for **2-DTC** in $CDCl_3$.

3-DTC: A yellow oil (yield: 90%). ^{1}H NMR (CDCl$_3$, δ, ppm): 5.8-6.0 (1H, $-CH_2C\underline{\mathbf{H}}(-OCH_2-)S-$), 4.0-4.1 (2H, $CH_3C\underline{\mathbf{H}}_2N<$), 3.7-3.8 (2H, $CH_3C\underline{\mathbf{H}}_2N<$), 3.3-3.6 (2H, $(CH_3)_2CHC\underline{\mathbf{H}}_2O-$), 2.9-3.0 (1H, $NCC\underline{\mathbf{H}}(CH_3)CH_2-$), 2.2-2.5 (2H, $>CHC\underline{\mathbf{H}}_2CH<$), 1.9 (1H, $(CH_3)_2C\underline{\mathbf{H}}-$), 1.5 (3H, $NCCH(C\underline{\mathbf{H}}_3)CH_2-$), 1.4 (6H, $-N(CH_2C\underline{\mathbf{H}}_3)_2$), 0.9-1.0 (6H, $(C\underline{\mathbf{H}}_3)_2CH-$). ^{13}C NMR (CDCl$_3$, δ, ppm): 194, 123, 92, 76, 49, 47, 41, 29, 23, 19, 18, 17, 13, 12. ESI-MS: m/z calc. for $C_{14}H_{26}N_2OS_2Na$: 325.14 $[M+Na]^+$; found: 325.14.

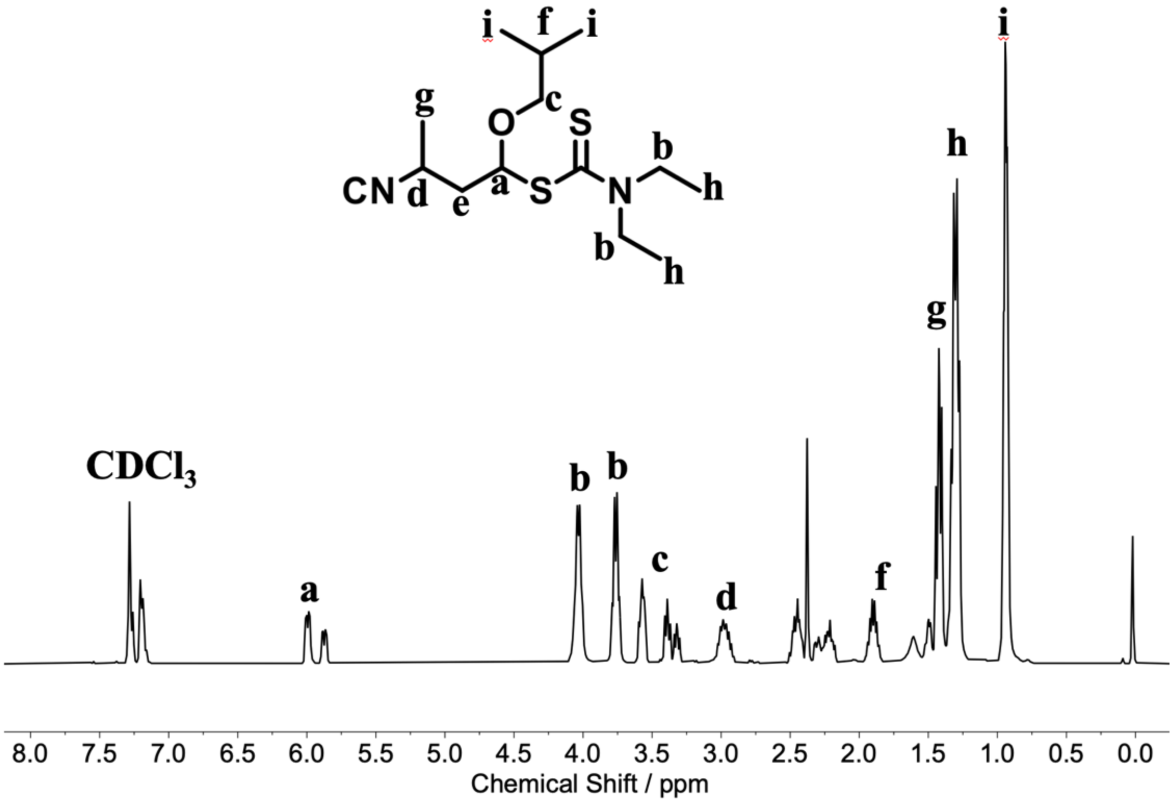

Supplementary Figure 22. ^{1}H NMR spectrum for **3-DTC** in CDCl$_3$.

4-DTC: A yellow solid (yield: 75%).^{1}H NMR ($CDCl_3$, δ, ppm): 6.8 and 7.4 (4H, aromatic proton), 5.3-5.5 (1H, $-CH_2C\underline{\mathbf{H}}(-Ph)S-$), 4.1 and 3.7 (4H, $-N(C\underline{\mathbf{H}}_2CH_3)_2$), 3.8 (3H, $-OC\underline{\mathbf{H}}_3$), 3.0-3.4 (3H, $-C\underline{\mathbf{H}}(O-)CH_3$ and $-C\underline{\mathbf{H}}_2O-$), 2.0-2.5 (2H, $>CHC\mathbf{H}_2CH<$), 1.7-1.9 (1H, $-C\underline{\mathbf{H}}(CH_3)_2$), 1.1-1.3 (9H, $-N(CH_2C\underline{\mathbf{H}}_3)_2$ and $-CH(O-)C\underline{\mathbf{H}}_3$), 0.9-1.1 (6H, $-CH(C\underline{\mathbf{H}}_3)_2$). ^{13}C NMR ($CDCl_3$, δ, ppm): 195, 159, 132, 129, 114, 76, 75, 74, 55, 51, 48, 45, 43, 42, 29, 19, 12, 11. ESI-MS: m/z calc. for $C_{20}H_{33}NO_2S_2Na$: 406.18 $[M+Na]^+$; found: 406.18.

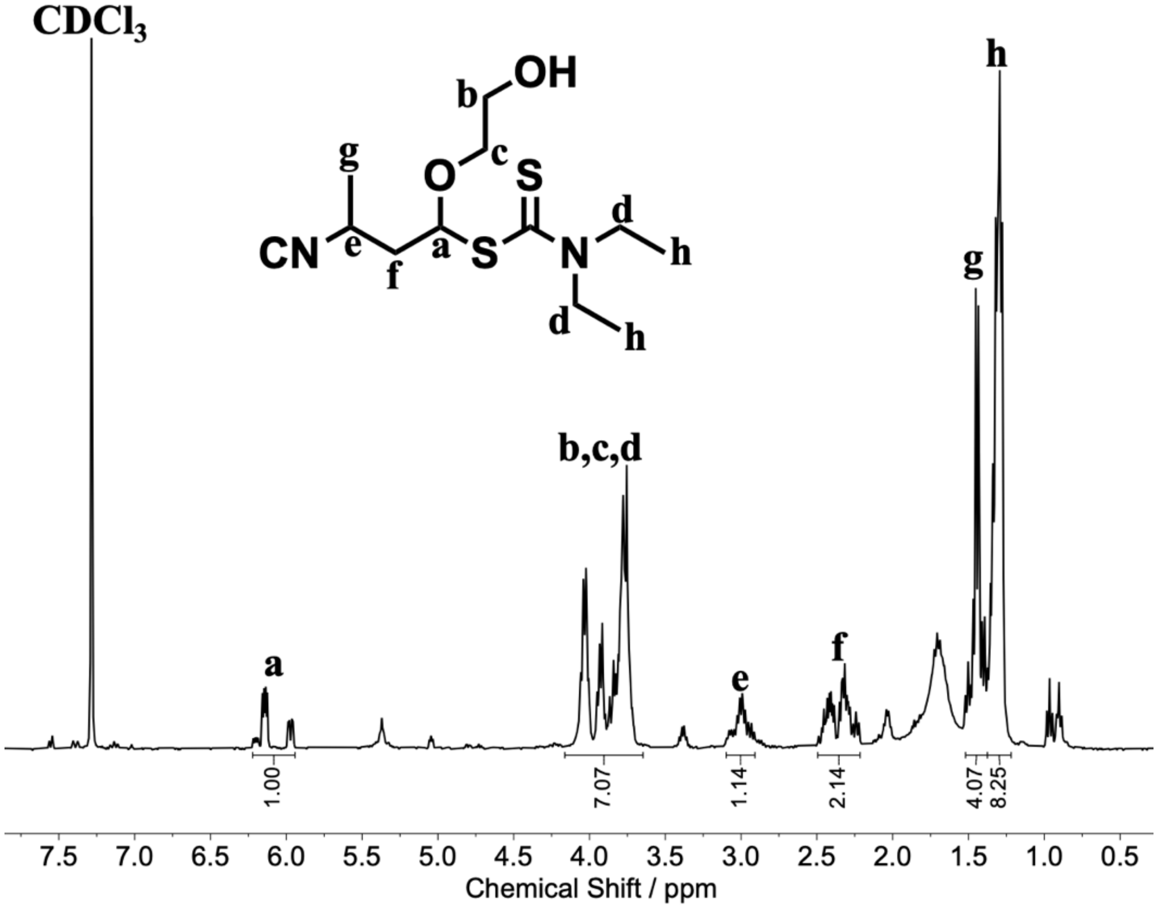

Supplementary Figure 23. ^{1}H NMR spectrum for **4-DTC** in $CDCl_3$.

5-DTC: A yellow solid (yield: 91 %). ^{1}H NMR ($CDCl_3$, δ, ppm): 6.9-7.4 (10H, aromatic proton), 5.7-6.1 (1H, -C**H**(O-)S-), 3.7 and 4.0 (4H, -N(C**H**$_2$CH$_3$)$_2$), 3.0-3.6 (5H, >C**H**CO-, -OC**H**$_2$-, and Ph-C**H**<), 2.2-2.5 (2H, >CHC**H**$_2$CH<), 1.5-2.0 (3H, -CH$_3$), 1,3 (6H, -N(CH$_2$C**H**$_3$)$_2$), 0.9 (6H, -C**H**$_3$). ^{13}C NMR ($CDCl_3$, δ, ppm): 194, 177, 176, 142, 141, 131, 129, 128, 127, 92, 52, 50, 48, 46, 40, 39, 38 37, 28, 26, 19, 18, 16, 13, 11. ESI-MS: m/z calc. for $C_{29}H_{38}N_2O_3S_2Na$: 549.22, $[M+Na]^+$; found: 549.22.

6-DTC: A yellow solid (yield: 90%). ^{1}H NMR ($CDCl_3$, δ, ppm): 5.7-5.8 (1H, -CH$_2$C**H**(OCH$_2$-)S-), 4.0-4.2 and 3.7-3.8 (6H, CH$_3$C**H**$_2$OOC-, -N(C**H**$_2$CH$_3$)$_2$), 3.6 (3H, >CH(COOC**H**$_3$)), 3.3-3.5 (2H, CH(CH$_3$)$_2$C**H**$_2$O-), 2.6-2.8 and 2.2-2.4 (4H, -OOCC(CH$_3$)$_2$C**H**$_2$ CH <, >CHC**H**$_2$CH<), 1.7-1.8 (1H, -C**H**(COOCH$_3$)-), 1.5 (1H, C**H**(CH$_3$)$_2$CH$_2$O-), 1.2-1.4 (15H, C**H**$_3$CH$_2$O-, -OOCC(C**H**$_3$)$_2$CH$_2$CH<, -N(CH$_2$C**H**$_3$)$_2$), 0.9-1.0 (6H, CH(C**H**$_3$)$_2$CH$_2$O-,). ^{13}C NMR ($CDCl_3$, δ, ppm): 195, 194, 177, 176, 94, 60, 52, 49, 47, 42, 40, 31, 30, 29, 25, 20, 14, 12, 11. ESI-MS: m/z calc. for $C_{21}H_{39}NO_5S_2Na$: 472.7. $[M+Na]^+$; found: 472.7.

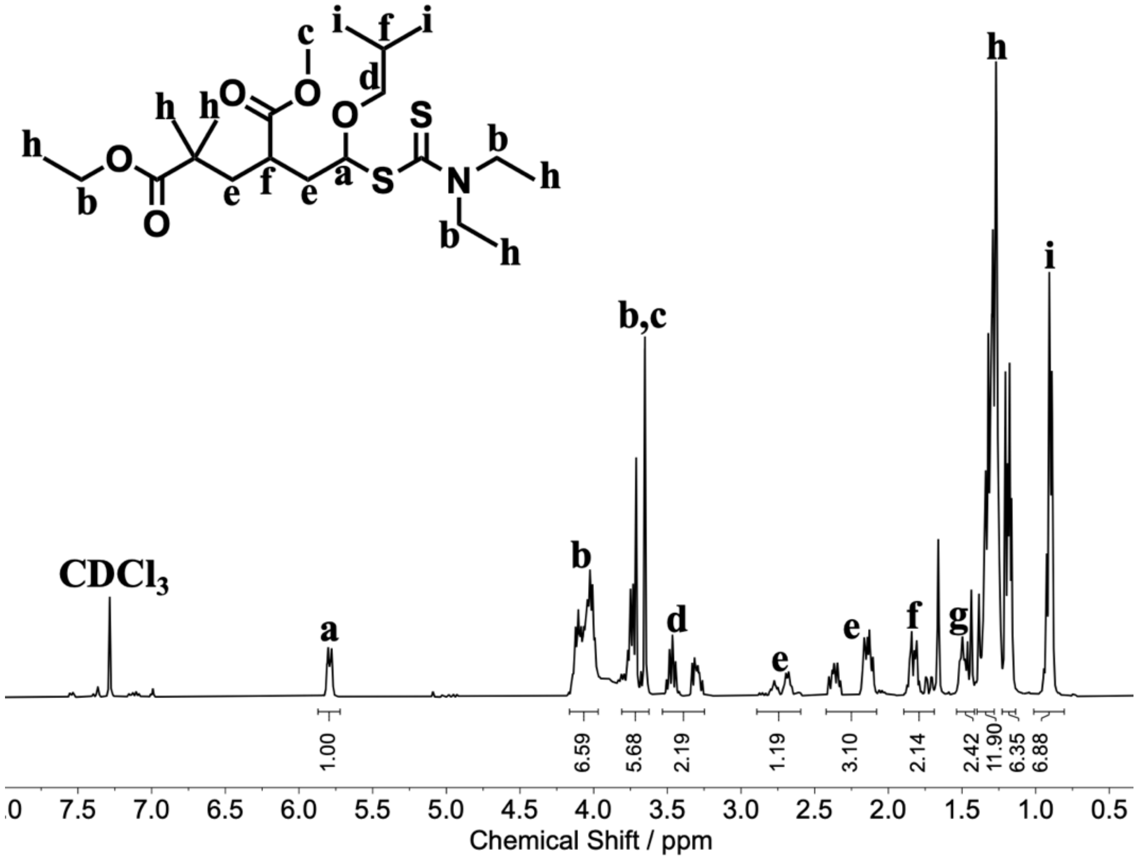

Supplementary Figure 24. ^{1}H NMR spectrum for **6-DTC** in $CDCl_3$.

7-DTC: A yellow solid (yield: 85%).A[1]H NMR ($CDCl_3$, δ, ppm): 6.8 and 7.4 (4H, aromatic proton), 5.3-5.5 (1H, $-CH_2C\underline{\mathbf{H}}(-Ph)S-$), 4.1 and 3.7 (4H, $-N(C\underline{\mathbf{H}}_2CH_3)_2$), 3.8 (3H, $-OC\underline{\mathbf{H}}_3$), 3.0-3.4 (3H, $-C\underline{\mathbf{H}}(O-)CH_3$ and $-C\underline{\mathbf{H}}_2O-$), 2.0-2.5 (2H, $>CHC\mathbf{H}_2CH<$), 1.7-1.9 (1H, $-C\underline{\mathbf{H}}(CH_3)_2$), 1.1-1.3 (9H, $-N(CH_2C\underline{\mathbf{H}}_3)_2$ and $-CH(O-)C\underline{\mathbf{H}}_3$), 0.9-1.1 (6H, $-CH(C\underline{\mathbf{H}}_3)_2$). ^{13}C NMR ($CDCl_3$, δ, ppm): 195, 159, 132, 129, 114, 76, 75, 74, 55, 51, 48, 45, 43, 42, 29, 19, 12, 11. ESI-MS: m/z calc. for $C_{20}H_{33}NO_2S_2Na$: 406.18 $[M+Na]^+$; found: 406.18.

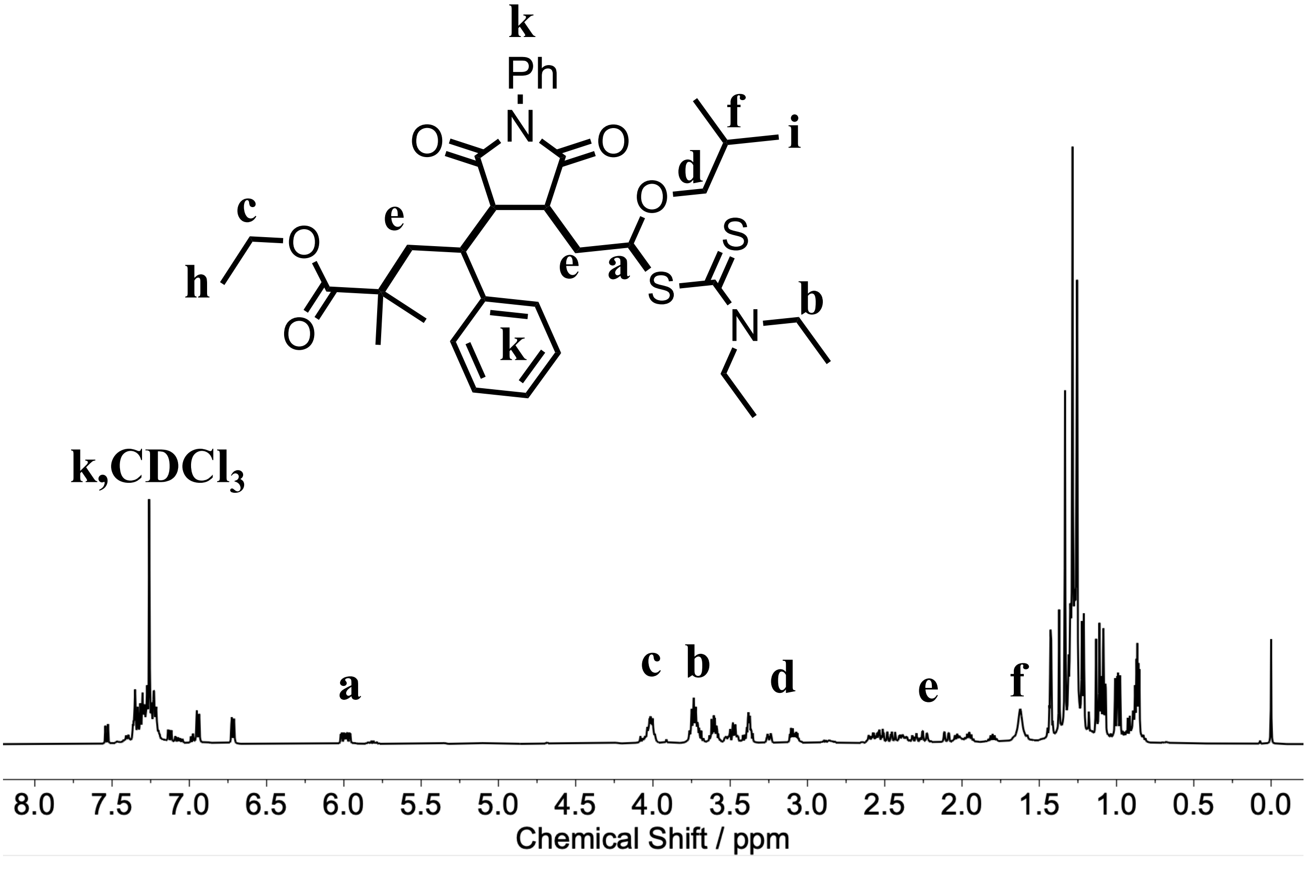

Supplementary Figure 25. ^1H NMR spectrum for **7-DTC** in $CDCl_3$.

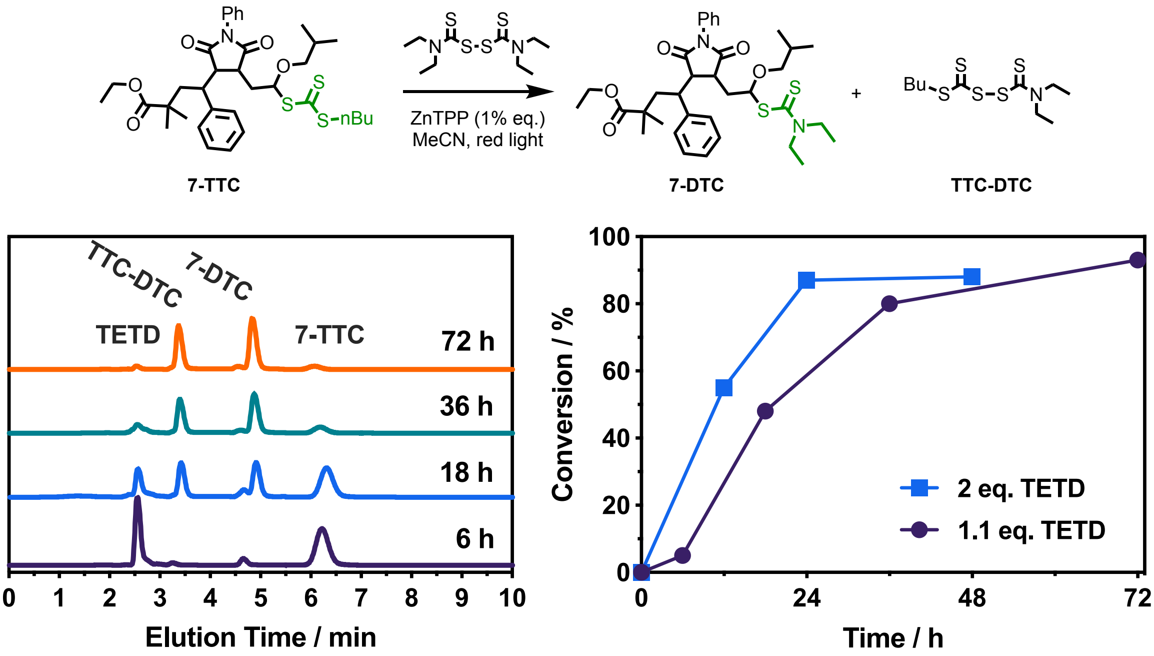

Supplementary Figure 26. Time-evolution of (a) HPLC traces (MeCN/H_2O 8/2 v/v; 300 nm absorbance) and (b) conversion recored for TTC-to-DTC transformation. Reaction was performed on 10 mmol scale using trithiocarbonate (iBVE-TTC, 1 eq.), TETD (1.1 or 2 eq.), and ZnTPP (1 mol %) in acetonitrile (MeCN) under red light irradiation (630 nm).

Insertion suitable monomer into dithiocarbamate which sythesized from trithiocarbonate

In a nitrogen filled glove box, dithiocarbamates (**1-DTC** or **5-DTC**) (1 mmol), suitable monomer (1-5 mmol), $FcPF_6$ (0.01 mmol), DCM (1 mL) were charged into an oven-dried 20 mL Schlenk tube equipped with a stir magneton. After being stirred for 24 h at 25 °C, the crude product was further purified by flash chromatography using PE/EA as the eluent.

8-DTC: A yellow solid (yield: 87 %). ^1H NMR ($CDCl_3$, δ, ppm): 6.9-7.5 (14H, aromatic proton), 5.2-5.6 (1H, -C$\underline{\mathbf{H}}$(-Ph)S-), 4.0-3.5 (8H, -N(C$\underline{\mathbf{H}}_2$$CH_3$)$_2$, -OC$\underline{\mathbf{H}}_3$, and Ph-C$\underline{\mathbf{H}}$<), 3.5-2.7 (5H, >C$\underline{\mathbf{H}}$CO-, -OC$\underline{\mathbf{H}}_2$-, and -OC$\underline{\mathbf{H}}$<), 2.2-1.5 (5H, >CHC$\mathbf{H}_2$CH< and >CHC$\mathbf{H}$($CH_3$)CH<), 1.5-0.8 (21H, -$CH_3$, -N($CH_2C\underline{\mathbf{H}}_3$)$_2$, and -C$\underline{\mathbf{H}}_3$). ^{13}C NMR ($CDCl_3$, δ, ppm): 194, 179, 178, 177, 159, 142, 141, 135, 132, 131, 129, 128, 127, 114, 58, 55, 52, 50, 48, 46, 40, 39, 38 37, 28, 26, 19, 18, 16, 13, 11. ESI-MS: m/z calc. for $C_{39}H_{50}N_2O_4S_2Na$: 697.31, $[M+Na]^+$; found: 697.31.

9-DTC: A yellow solid (yield: 90%). ^{1}H NMR ($CDCl_3$, δ, ppm): 6.9-8.1 (15H, aromatic proton), 5.9-6.2 (1H, -C**H**(-O-)S-), 4.4 (2H, -COO-C**H**$_2$-), 4.0-3.5 (7H, -N(C**H**$_2$CH$_3$)$_2$, -OC**H**$_2$-, and -OC**H**<), 3.5-2.7 (5H, Ph-C**H**<, >C**H**CO-, -OC**H**$_2$-, and -OC**H**<), 2.2-1.5 (6H, >CHC**H**$_2$CH< and >CHC**H**(CH$_3$)CH<), 1.5-0.8 (25H, -N(CH$_2$C**H**$_3$)$_2$, and -C**H**$_3$). ^{13}C NMR ($CDCl_3$, δ, ppm): 194, 179, 178, 177, 159, 142, 141, 135, 132, 131, 129, 128, 127, 114, 58, 55, 52, 50, 48, 46, 40, 39, 38 37, 28, 26, 19, 18, 16, 13, 11. ESI-MS: m/z calc. for $C_{40}H_{50}N_2O_6S_2Na$: 741.31, [M+Na]$^+$; found: 741.31.

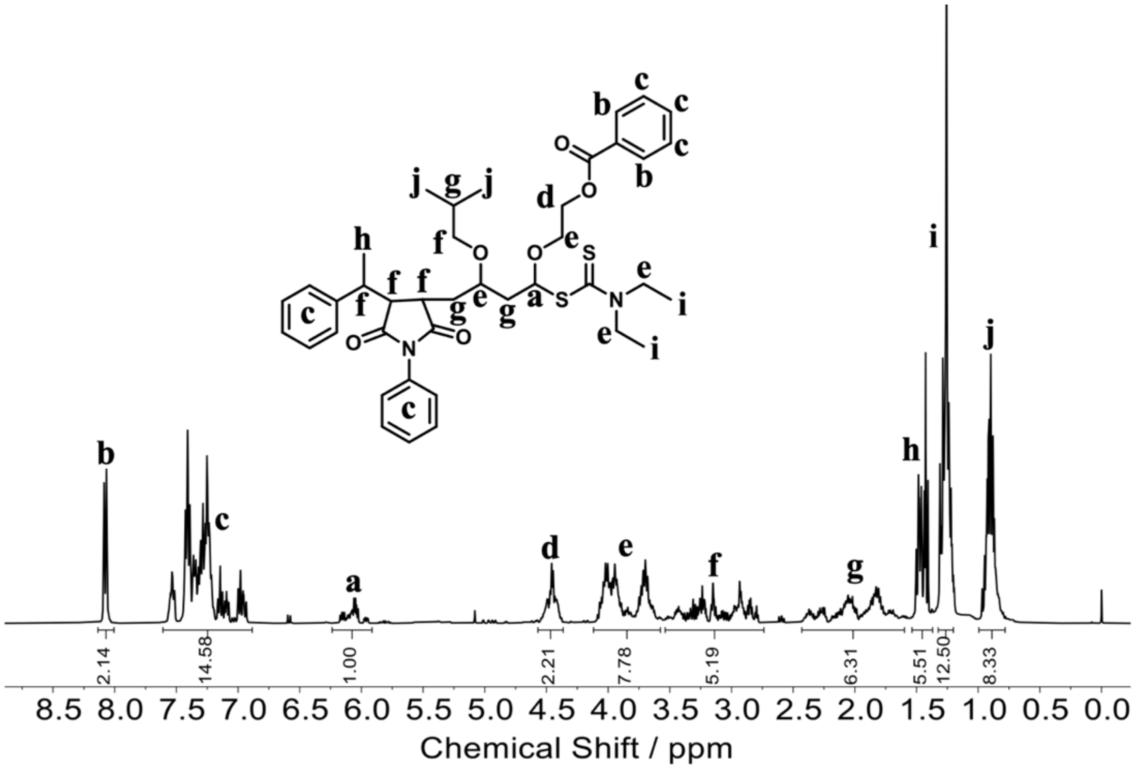

Supplementary Figure 27. ^{1}H NMR spectrum for **9-DTC** in $CDCl_3$.

10-DTC: A yellow oil (yield: 48%). ^{1}H NMR ($CDCl_3$, δ, ppm): 6.0 (1H, $-CH_2C\underline{\mathbf{H}}(-O-)S-$), 4.1-4.2 (2H, $CH_3C\underline{\mathbf{H}}_2OOCCH<$), 4.0-4.1 and 3.4-3.6 (4H, $-N(-C\underline{\mathbf{H}}_2CH_3)_2$), 3.4-3.6 (2H, $-OC\underline{\mathbf{H}}_2CH_2CH_2CH_3$), 3.1-3.4 (2H, $-OC\underline{\mathbf{H}}_2CH(CH_3)_2$), 1.8-2.6 (5H, $>CHC\underline{\mathbf{H}}_2C\underline{\mathbf{H}}(-O-)C\underline{\mathbf{H}}_2CH<$), 1.4-1.6 (6H, $-OOCC\underline{\mathbf{H}}(-CH_3)-$, $-CH_2C\underline{\mathbf{H}}(-CH_3)_2$, $-OCH_2C\underline{\mathbf{H}}_2C\underline{\mathbf{H}}_2CH_3$), 1.2-1.4 (12H, $C\underline{\mathbf{H}}_3CH_2O-$, $-OOCCH(-C\underline{\mathbf{H}}_3)-$, $-N(-CH_2C\underline{\mathbf{H}}_3)_2$), 0.9 (9H, $-CH_2CH(-C\underline{\mathbf{H}}_3)_2$, $-OCH_2CH_2CH_2C\underline{\mathbf{H}}_3$). ^{13}C NMR ($CDCl_3$, δ, ppm): 196, 103, 93, 74, 69, 60, 49, 47, 42, 39, 36, 32, 31, 30, 29, 20, 14, 13, 12. LC-MS: m/z calc. for $C_{22}H_{43}NO_4S_2Na$: 472.72 $[M+Na]^+$; found: 472.72.

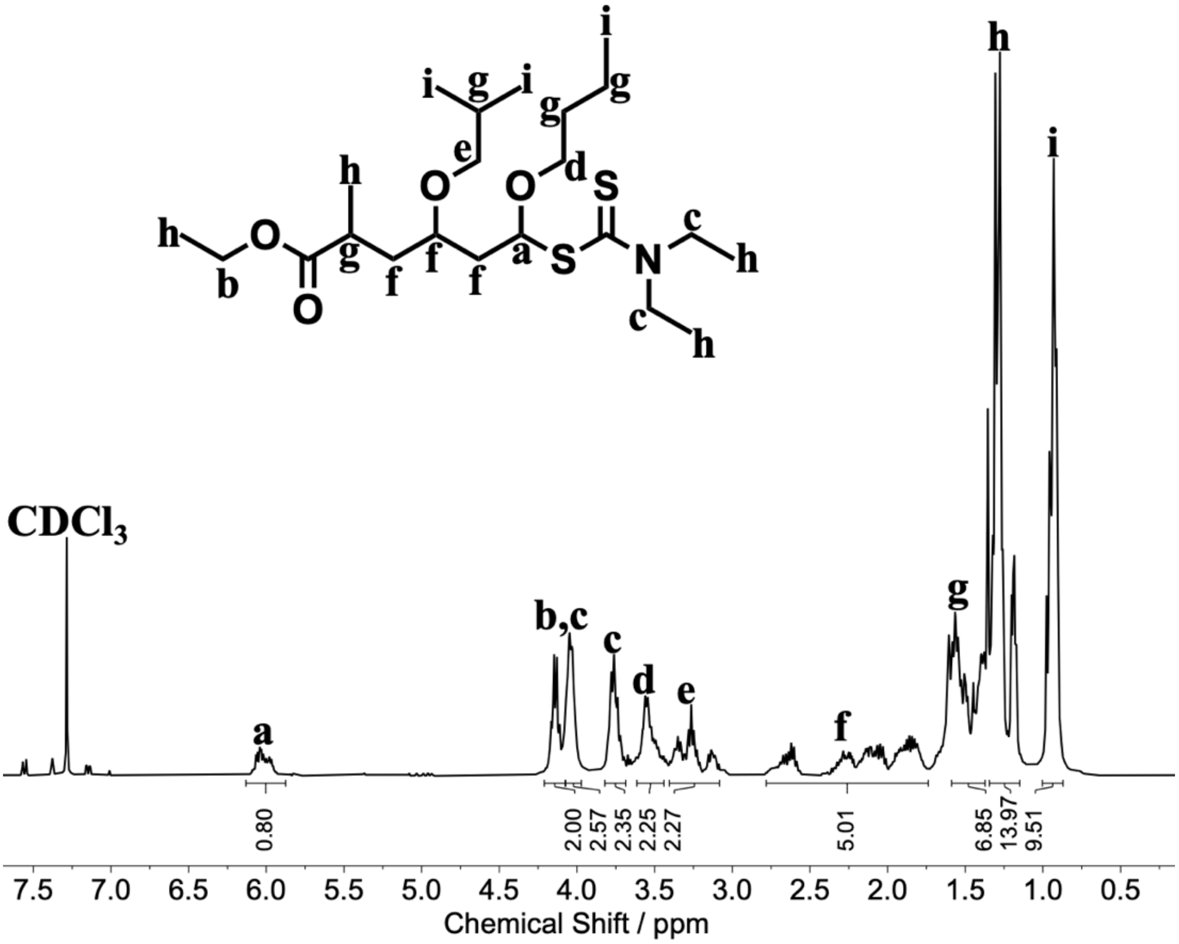

Supplementary Figure 28. ^{1}H NMR spectrum for **10-DTC** in $CDCl_3$.

Insertion BzEVE into 10-DTC via second-step cationic SUMI

In a nitrogen filled glove box, **10-DTC** (1 mmol), BzEVE (1.5 mmol), $FcPF_6$ (0.05 mmol), DCM (1 mL) were charged into an oven-dried 20 mL Schlenk tube equipped with a stir magneton. After being stirred for 24 h at 25 °C, the crude product was further purified by flash chromatography using PE/EA as the eluent affording a yellow solid (yield: 78%). ^{1}H NMR ($CDCl_3$, δ, ppm): 8.0-8.2 and 7.4-7.7 (5H, aromatic proton), 6.2 (1H, -$CH_2C\underline{\mathbf{H}}$(-O-)S-), 4.5 (2H, PhCOO$C\underline{\mathbf{H}}_2$CH-$_2$O-), 4.0-4.2 (6H, $CH_3C\underline{\mathbf{H}}_2$OOCCH<, -N($C\underline{\mathbf{H}}_2CH_3)_2$), 3.1-3.8 (9H, -O$C\underline{\mathbf{H}}_2CH(CH_3)_2$, -O$C\underline{\mathbf{H}}_2CH_2CH_2CH_3$, -O$C\underline{\mathbf{H}}_2CH_2$OOC-, -OOCCH($CH_3$)$C\underline{\mathbf{H}}_2$CH<, >$C\underline{\mathbf{H}}CH_2$CH(-O-)S-), 2.1-2.8 (4H, >CH$C\underline{\mathbf{H}}_2$CH(-O-)S-, -CH(-O-)$C\underline{\mathbf{H}}_2$CH(-O-)-), 1.7-2.0 (3H, -$OCH_2C\underline{\mathbf{H}}(CH_3)_2$, -$C\underline{\mathbf{H}}(CH_3)CH_2C\underline{\mathbf{H}}$(-O-)-), 1.4-1.7 (4H, -$OCH_2C\underline{\mathbf{H}}_2C\underline{\mathbf{H}}_2CH_3$), 1.3-1.4 (9H, $C\underline{\mathbf{H}}_3CH_2$OOCCH-, -N($CH_2C\underline{\mathbf{H}}_3)_2$), 1.2-1.3 (3H, -OOCCH($C\underline{\mathbf{H}}_3$)$CH_2$-), 0.9-1.0 (9H, -$CH_2CH(C\underline{\mathbf{H}}_3)_2$, -$OCH_2CH_2CH_2C\underline{\mathbf{H}}_3$). ^{13}C NMR ($CDCl_3$, δ, ppm): 180, 166, 134, 130, 129, 75, 66, 64, 61, 60, 49, 47, 36, 33, 30, 29, 20, 14, 12. LC-MS: m/z calc. for $C_{33}H_{55}NO_7S_2Na$: 664.9 $[M+Na]^+$; found: 664.9.

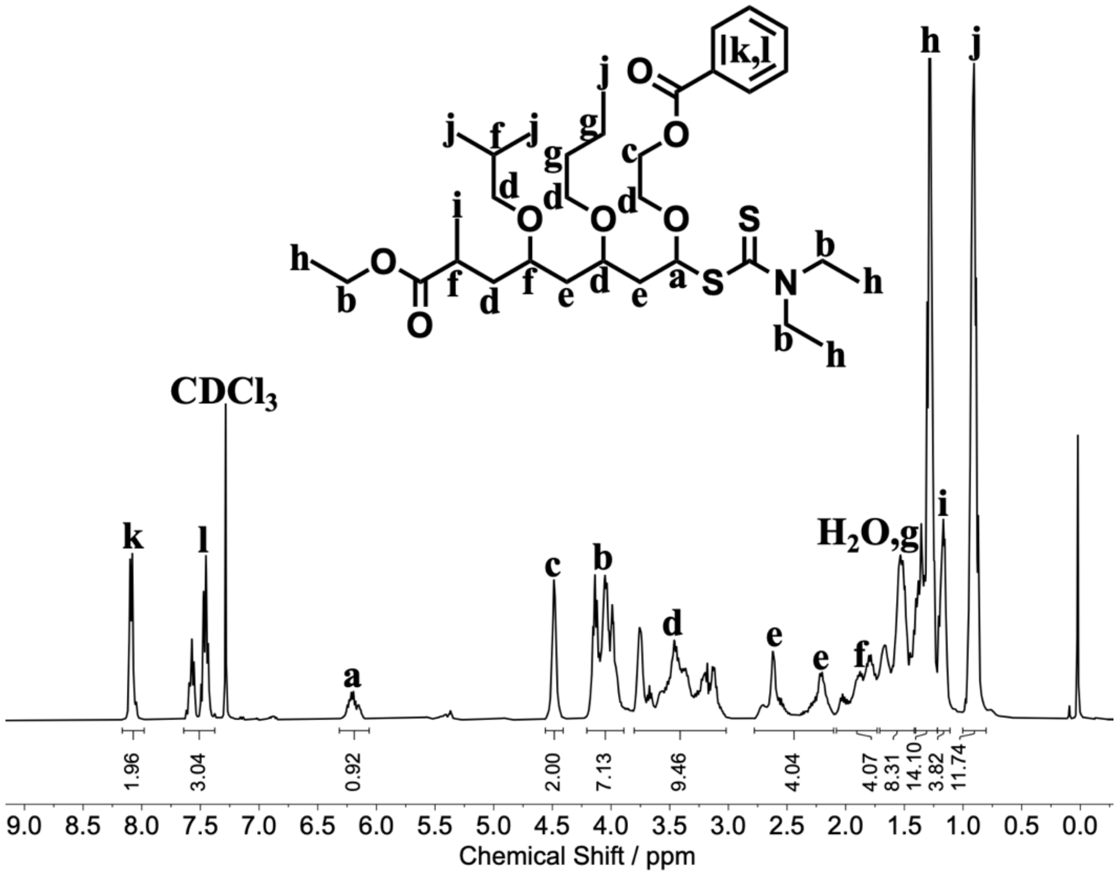

Supplementary Figure 29. ^{1}H NMR spectrum for **11-DTC** in $CDCl_3$.

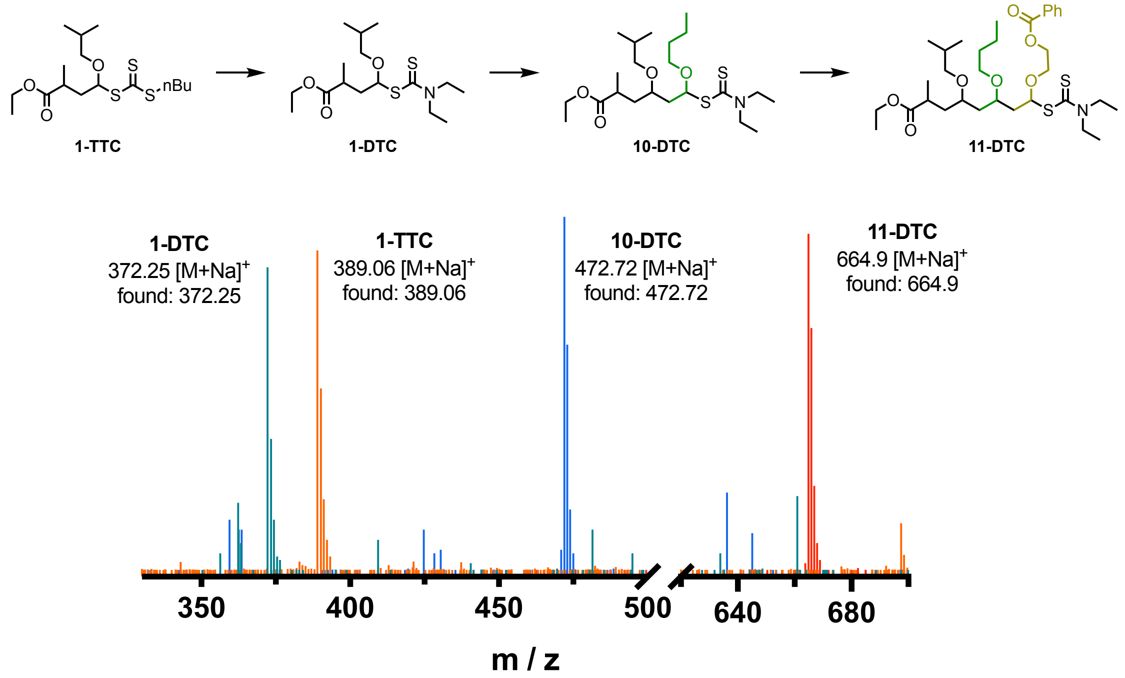

Supplementary Figure 30. ESI MS spectra for **1-TTC**, **1-DTC**, **10-DTC**, and **11-DTC**.

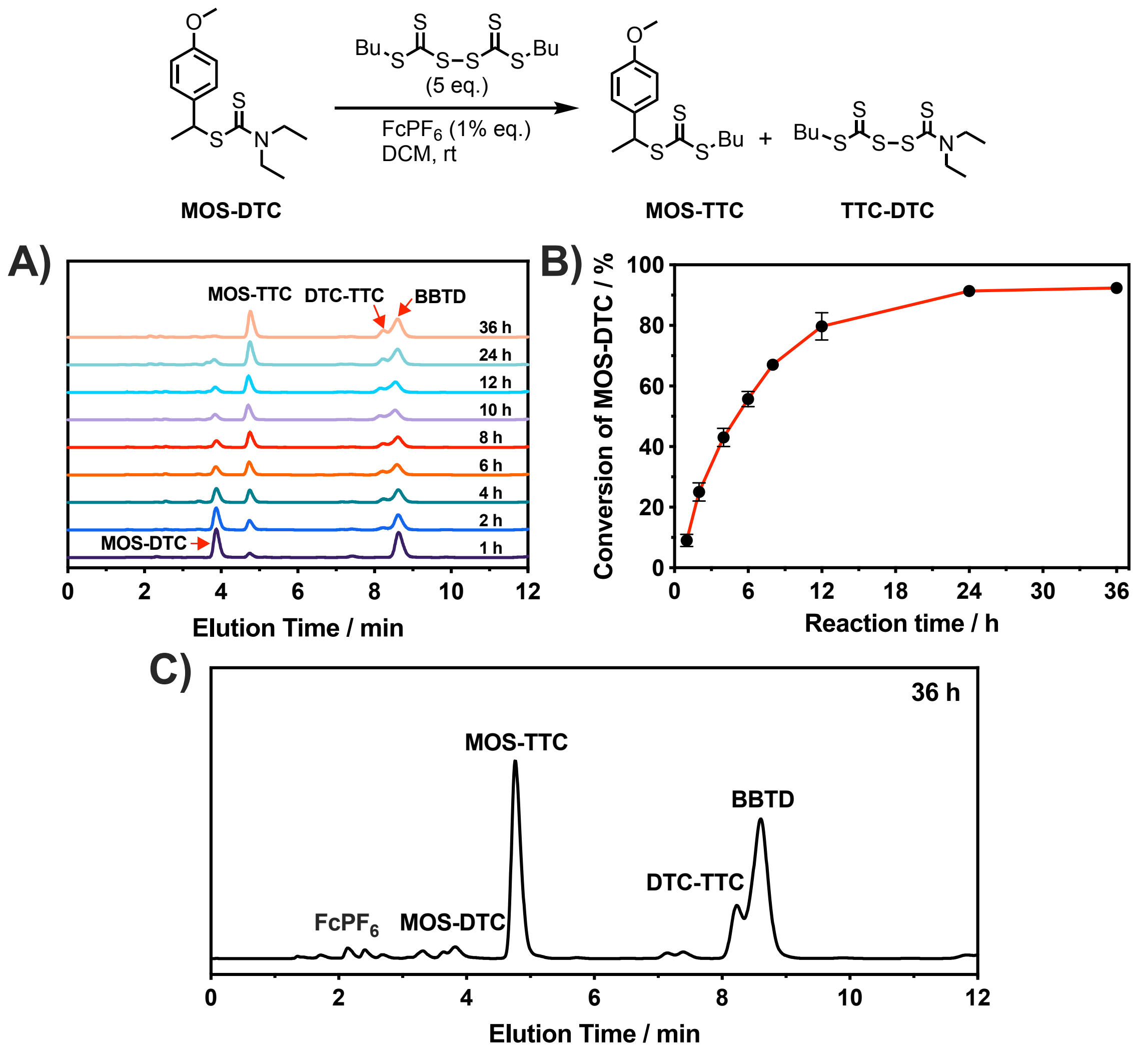

Supplementary Figure 31. Time-evolution of (a and c) HPLC traces (MeCN/H_2O 75/25 v/v; 300 nm absorbance) and (b) conversion recored for DTC-to-TTC transformation. Reaction was performed on 10 mmol scale using **MOS-DTC** (1 eq.), BBTD (5 eq.), $FcPF_6$ (1 mol %) in DCM. The mean and error (±s.d.) were obtained from three independent experiments (n = 3).

Synthesis of dithiocarbamates via cationic SUMI.

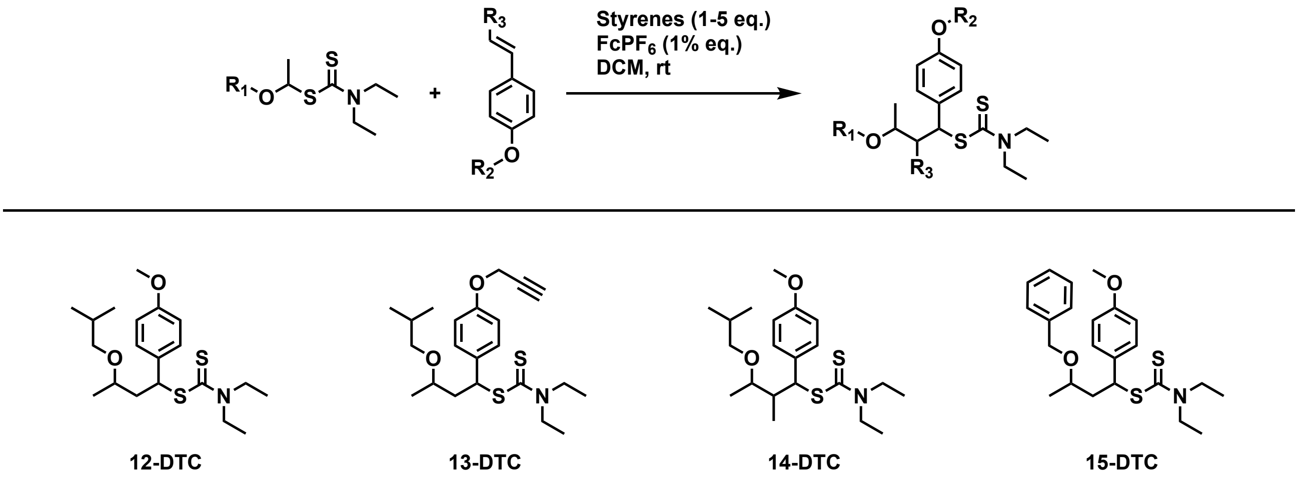

Supplementary Figure 32. Synthesis route for dimer dithiocarbamates (**12-DTC - 15-DTC**).

In a nitrogen filled glove box, dithiocarbamates (1 mmol), styrenes (1-5 mmol), $FcPF_6$ (0.01 mmol), DCM (1 mL) were charged into an oven-dried 20 mL Schlenk tube equipped with a stir magneton. After being stirred for 24 h at 25 °C, the crude product was further purified by flash chromatography using PE/EA as the eluent.

15-DTC: A yellow solid (yield: 52%). ^{1}H NMR ($CDCl_3$, δ, ppm): 7.3-7.5 and 6.8 (9H, aromatic proton), 5.4-5.5 (1H, $-CH_2C\underline{\mathbf{H}}(-Ph-)S-$), 4.5 (2H, $PhC\underline{\mathbf{H}}_2OCH<$), 4.0 and 3.7 (4H, $-N(C\underline{\mathbf{H}}_2CH_3)_2$), 3.8 (3H, $-OC\underline{\mathbf{H}}_3$), 2.0-2.7 (2H, $>CHC\underline{\mathbf{H}}_2CH(-Ph-)S-$), 1.4 (1H, $PhCH_2OC\underline{\mathbf{H}}(CH_3)-$), 1.2-1.3 (9H, $C\underline{\mathbf{H}}_3CH(-O-)CH_2-$, $-N(CH_2C\underline{\mathbf{H}}_3)_2$). ^{13}C NMR ($CDCl_3$, δ, ppm): 195, 159, 139, 134, 130, 129, 128, 114, 74, 70, 55, 54, 49, 46, 44, 30, 20, 13,12. ESI-MS: m/z calc. for $C_{23}H_{32}NO_2S_2$: 418.6. $[M+H]^+$; found: 418.6.

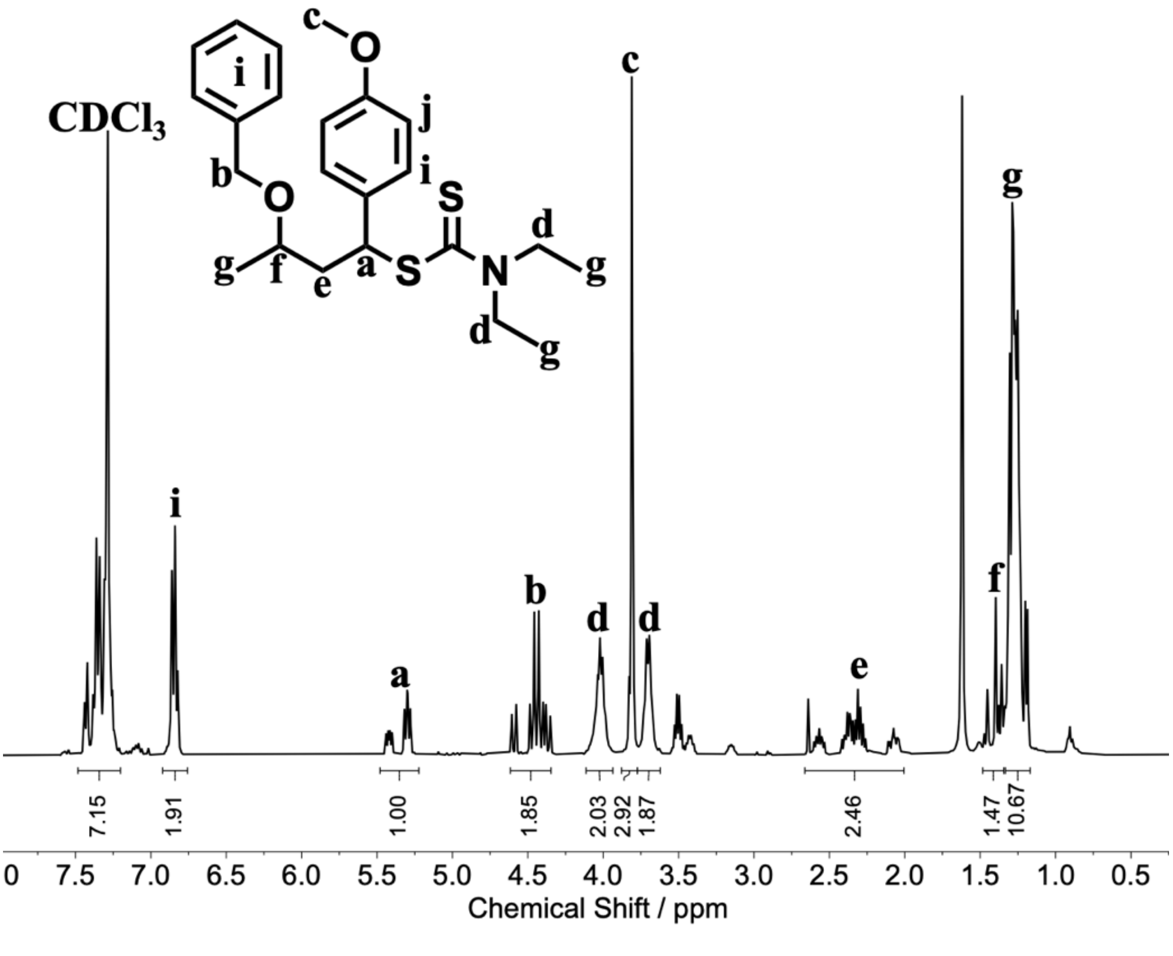

Supplementary Figure 33. ^{1}H NMR spectrum for **15-DTC** in $CDCl_3$.

Synthesis of dithiocarbamates via two-step cationic SUMI.

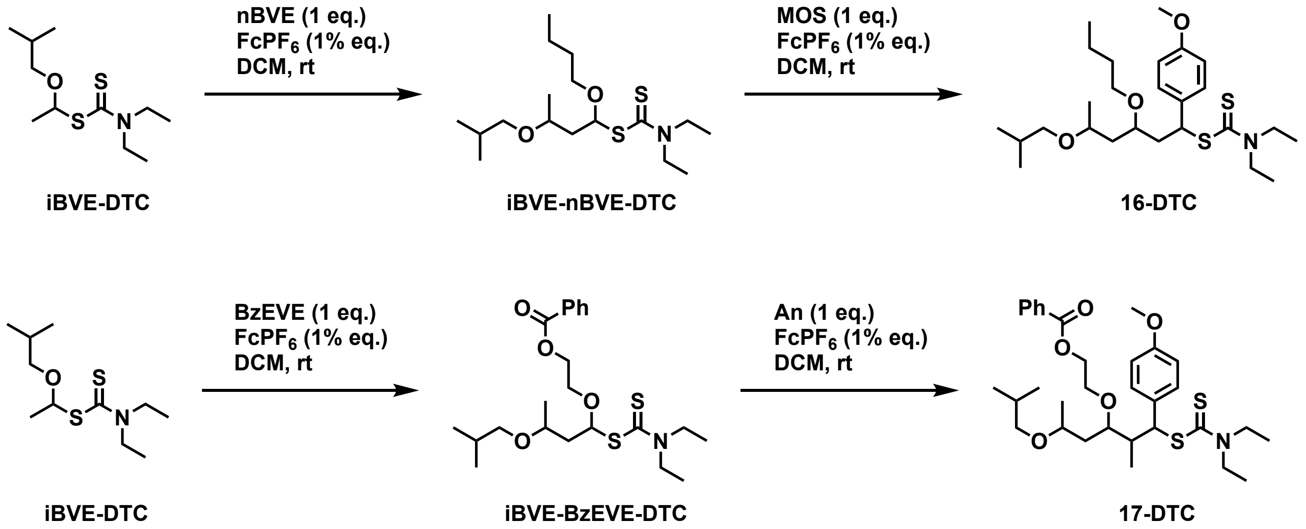

Supplementary Figure 34. Synthesis route for dithiocarbamates (**16-DTC** and **17-DTC**).

Synthesis of 17-DTC: In a nitrogen filled glove box, **iBVE-BzEVE-DTC** (5 mmol), **An** (25 mmol), $FcPF_6$ (0.25 mmol), DCM (5 mL) were charged into an oven-dried 20 mL Schlenk tube equipped with a stir magneton. The mixture was stirred at 25 °C for 24~72 h. The crude product was further purified by flash chromatography using PE/EA (*v*/*v* = 20/1~5/1) as the eluent, affording **18-DTC** as a yellow solid (yield: 83 %). ^{1}H NMR ($CDCl_3$, δ, ppm): 6.7-8.1 (9H, aromatic proton), 5.0-5.5 (1H, -C**H**(-Ph)S-), 4.5 (2H, -COOC**H**$_2$-), 2.7-4.0 (13H, -N(C**H**$_2$CH$_3$)$_2$, -COOCH$_2$C**H**$_2$O-, -C**H**(O-)CH$_3$, -C**H**$_2$O-, and -OC**H**$_3$), 1.5-2.2 (4H, >CHC**H**$_2$CH<, >CHC**H**(CH$_3$)CH<, and -C**H**(CH$_3$)$_2$), 0.7-1.3 (18H, -N(CH$_2$C**H**$_3$)$_2$, -CH(O-)C**H**$_3$, >CHCH(C**H**$_3$)CH<, and -CH(C**H**$_3$)$_2$). ^{13}C NMR ($CDCl_3$, δ, ppm): 194, 166, 157, 134, 133, 130, 129, 113, 76, 75, 72, 67, 64, 49, 46, 45, 44, 29, 27, 20, 13, 12. ESI-MS: m/z calc. for $C_{32}H_{47}NO_5S_2Na$: 612.28 [M+Na]$^+$; found: 612.28.

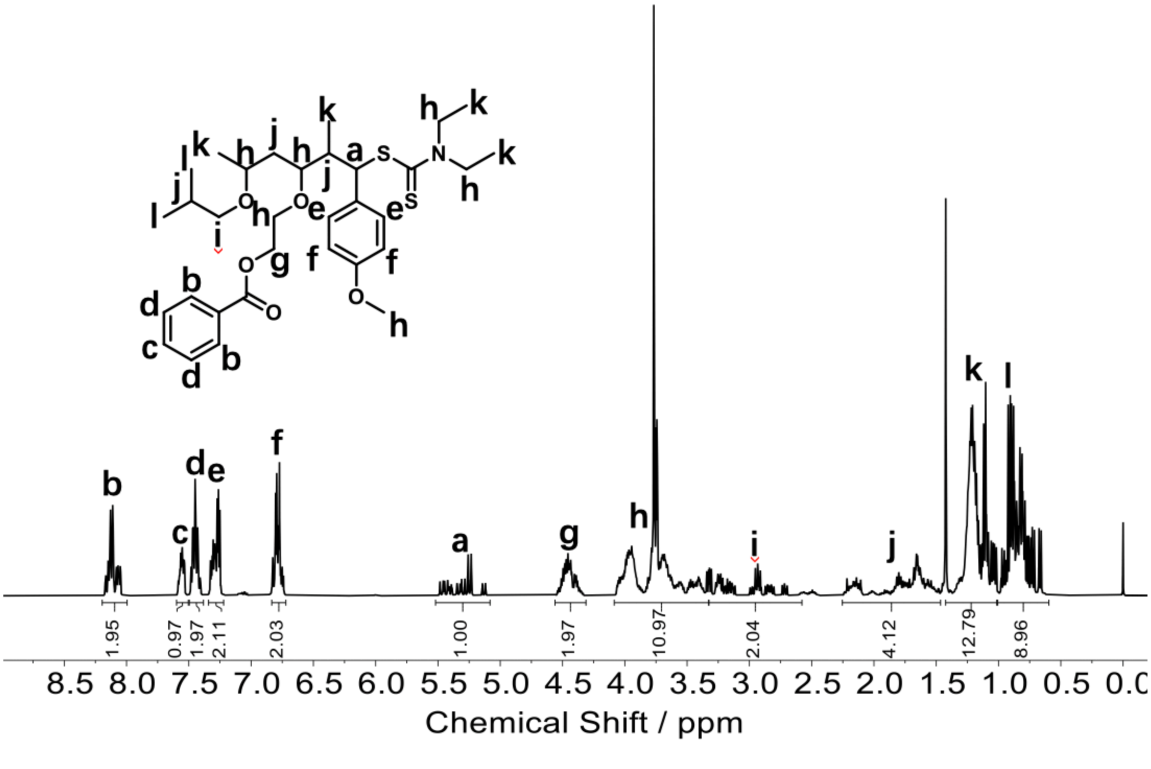

Supplementary Figure 35. ^{1}H NMR spectrum for **17-DTC** in $CDCl_3$.

Transfor from dithiocarbamate to trithiocarbonate via radical process

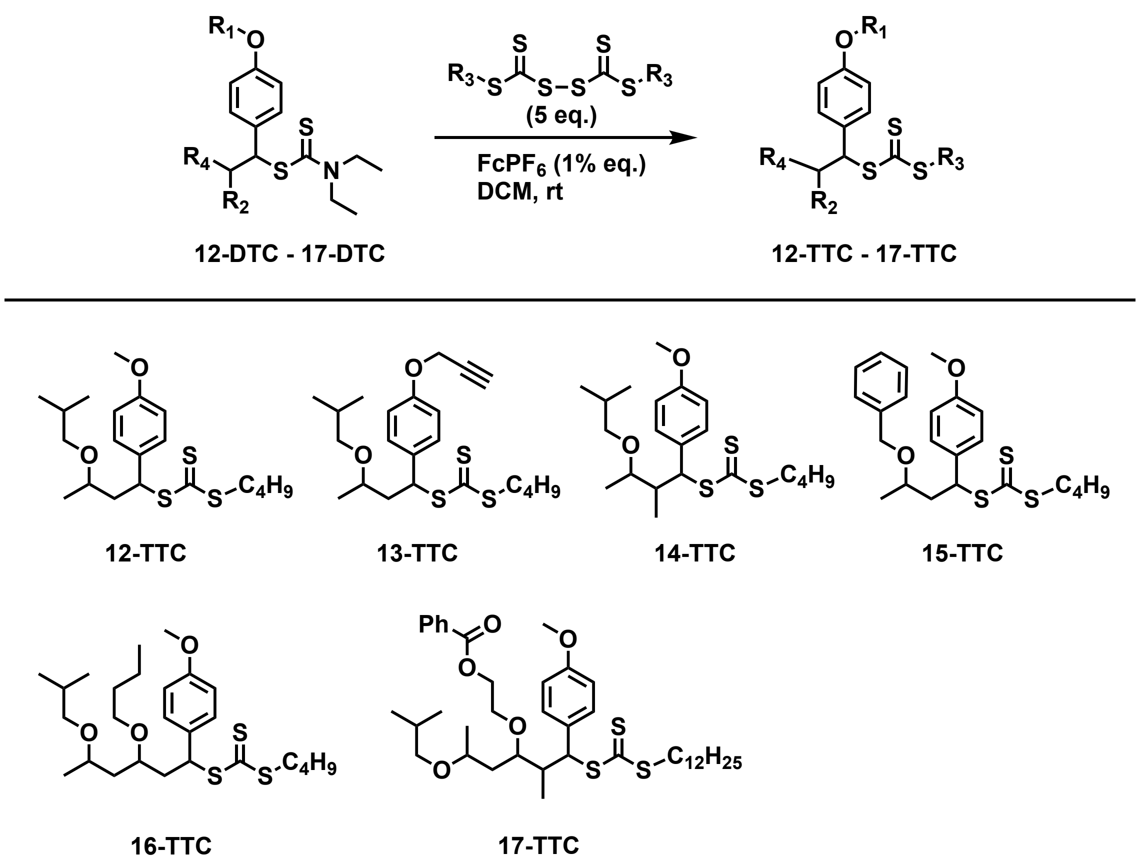

Supplementary Figure 36. Synthesis route for trithiocarbonates (**12-TTC – 17-TTC**) from dithiocarbamates (**12-DTC – 17-DTC**).

In a nitrogen filled glove box, dithiocarbamate (1 equiv), BBTD/BDTD (5 equiv), $FcPF_6$ (5 mol %), and DCM were charged into an oven-dried 20 mL Schlenk tube equipped with a stir magneton. The mixture was stirred at room temperature for 24 h. The crude product was purified by flash chromatography using PE/EA as the eluent.

12-TTC: A yellow oil (yield: 92%). ^{1}H NMR (CDCl$_3$, δ, ppm): 6.8 and 7.3 (4H, aromatic proton), 5.3-5.5 (1H, -CH$_2$C**H**(-Ph)S-), 3.8-3.9 (3H, -OC**H**$_3$), 3.3-3.5 (2H, -SC**H**$_2$CH$_2$-), 2.7-3.3 (3H, -C**H**(O-)CH$_3$ and -C**H**$_2$O-), 1.9-2.4 (2H, >CHC**H**$_2$CH<), 1.8-1.9 (1H, -C**H**(CH$_3$)$_2$), 1.6-1.8 (2H, - CH$_2$C**H**$_2$CH$_2$-), 1.4-1.5 (2H, -CH$_2$C**H**$_2$CH$_3$), 1.2-1.4 (3H, -CH(O-)C**H**$_3$), 0.9-1.2 (9H, -CH(C**H**$_3$)$_2$ and - CH$_2$C**H**$_3$). ^{13}C NMR (CDCl$_3$, δ, ppm): 222, 159, 133, 132, 129, 114, 76, 72, 55, 51, 43, 36, 31, 30, 29, 22, 20, 14. ESI-MS: m/z calc. for $C_{20}H_{32}O_2S_3Na$: 423.12, [M+Na]$^+$; found: 423.12.

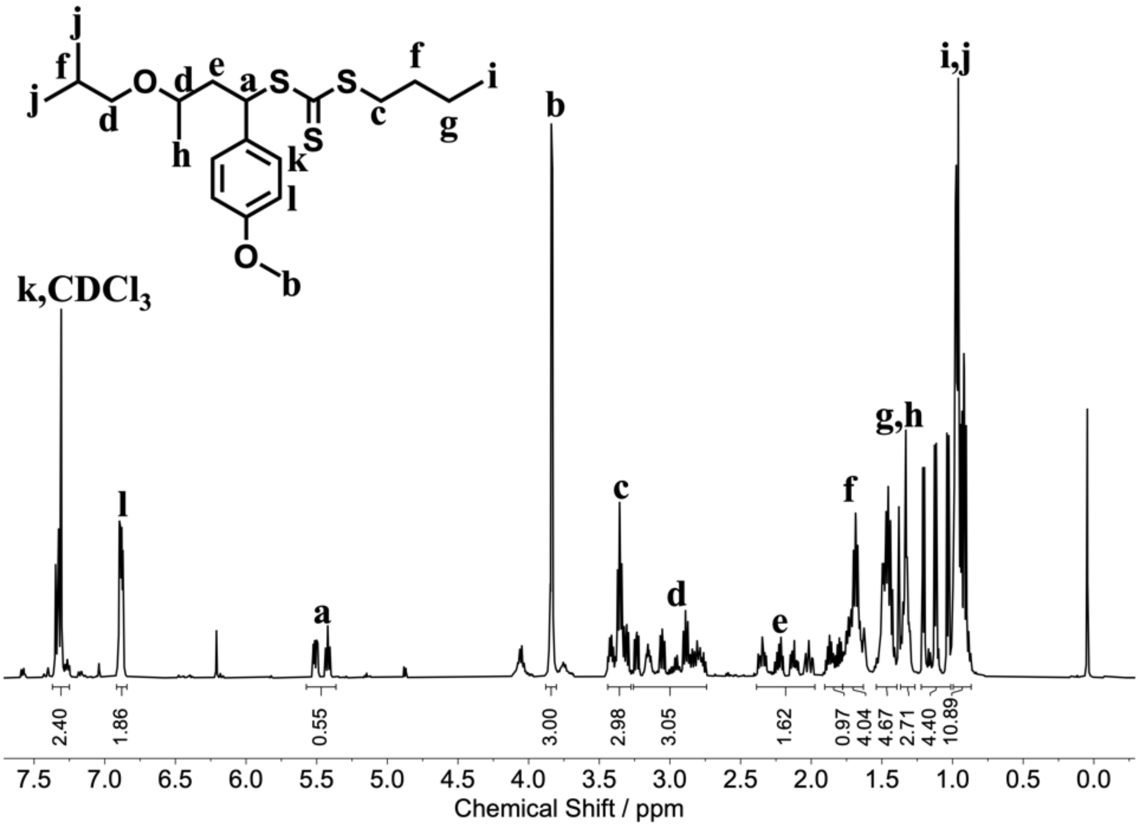

Supplementary Figure 37. ^{1}H NMR spectrum for **12-TTC** in CDCl$_3$.

13-TTC: A yellow solid (yield: 65%). ^{1}H NMR (CDCl$_3$, δ, ppm): 7.4 and 6.9-7.0 (4H, aromatic proton), 5.4-5.5 (1H, -CH$_2$C**H**(-Ph-)S-), 4.7 (2H, -C**H**$_2$OPh-), 2.8-3.5 (5H, -OC**H**$_2$CH(CH$_3$)$_2$, CH$_3$C**H**(-O-)CH$_2$-, -SC**H**$_2$CH$_2$CH$_2$CH$_3$), 1.8-2.5 (4H, CH$_3$CH(-O-)C**H**$_2$CH<, C**H**CCH$_2$OPh-, -OCH$_2$C**H**(CH$_3$)$_2$), 1.7 and 1.5 (4H, -SCH$_2$ C**H**$_2$ C**H**$_2$ CH$_3$), 1.2-1.3 (3H, C**H**$_3$CH(-O-)-), 0.8-1.1 (9H, -OCH$_2$CH(C**H**$_3$)$_2$, -SCH$_2$ CH$_2$ CH$_2$ C**H**$_3$). ^{13}C NMR (CDCl$_3$, δ, ppm): 157, 135, 134, 129, 115, 79, 75, 73, 56, 52, 44, 36, 30, 29, 22, 20, 14. ESI-MS: m/z calc. for $C_{22}H_{32}O_2S_3Na$: 447.68 $[M+Na]^+$; found: 447.68.

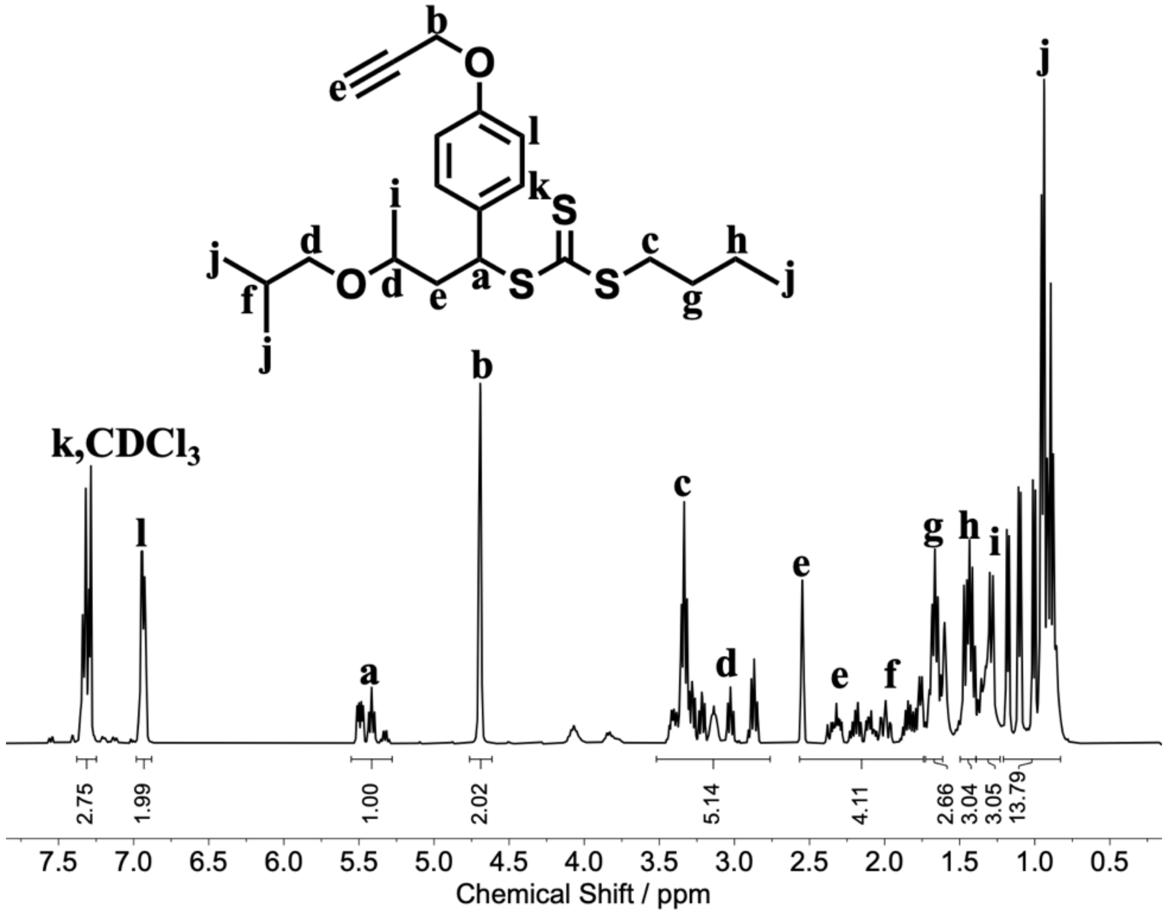

Supplementary Figure 38. ^{1}H NMR spectrum for **13-TTC** in CDCl$_3$.

14-TTC: A yellow solid (yield: 87%). ^{1}H NMR ($CDCl_3$, δ, ppm): 7.3 and 6.8 (4H, aromatic proton), 5.4-5.5 (1H, >CHC**H**(-Ph-)S-), 3.8 (3H, C**H**$_3$OPh-), 2.7-3.4 (5H, -OC**H**$_2$CH(CH$_3$)$_2$, CH$_3$C**H**(-O-)CH<, -SC**H**$_2$CH$_2$CH$_2$CH$_3$), 1.8-2.0 (2H, >C**H**CH(-Ph-)S-, -OCH$_2$C**H**(CH$_3$)$_2$), 1.7 and 1.5 (4H, -SCH$_2$ C**H**$_2$ C**H**$_2$ CH$_3$), 0.8-1.2 (15H, C**H**$_3$CH(-O-)CH(C**H**$_3$)CH<, -OCH$_2$CH(C**H**$_3$)$_2$, -SCH$_2$CH$_2$CH$_2$C**H**$_3$). ^{13}C NMR ($CDCl_3$, δ, ppm): 158, 134, 130, 114, 76, 75, 74, 59, 55, 45, 44, 36, 30, 29, 22, 20, 18, 14, 12.

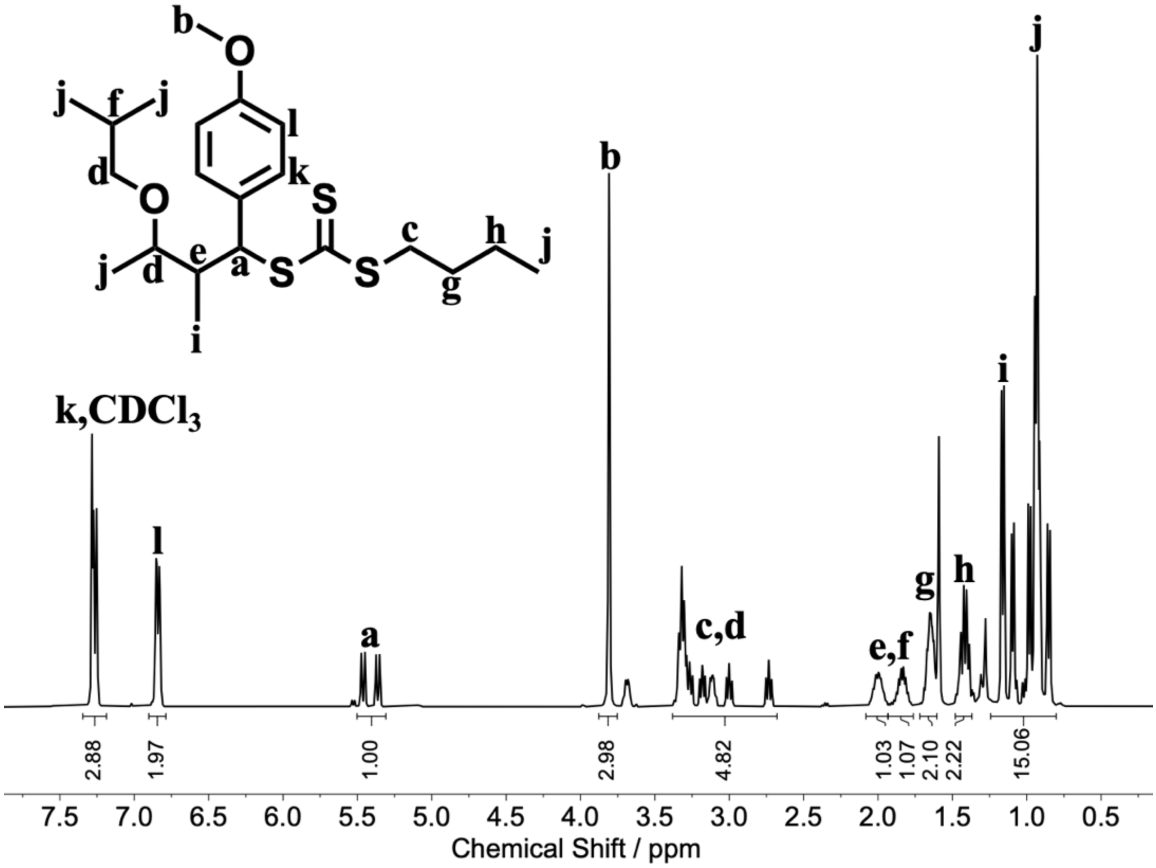

Supplementary Figure 39. ^{1}H NMR spectrum for **14-TTC** in $CDCl_3$.

15-TTC: A yellow solid (yield: 80%). ^{1}H NMR ($CDCl_3$, δ, ppm): 7.3-7.5 and 6.8 (9H, aromatic proton), 5.5 (1H, -CH$_2$C**H**(-Ph-)S-), 4.5 (2H, PhC**H**$_2$OCH<), 3.8 (3H, -OC**H**$_3$), 3.3-3.4 (2H, -SC**H**$_2$CH$_2$CH$_2$CH$_3$), 2.0-2.5 (2H, >CHC**H**$_2$CH(-Ph-)S-), 1.7 and 1.4 (4H, -SCH$_2$C**H**$_2$C**H**$_2$CH$_3$) 1.2-1.3 (4H, C**H**$_3$C**H**(-O-)CH$_2$-), 0.9-1.0 (3H, -SCH$_2$CH$_2$CH$_2$C**H**$_3$). ^{13}C NMR ($CDCl_3$, δ, ppm): 159, 139, 133, 130, 129, 128, 114, 72, 70, 55, 52, 43, 37, 30, 23, 20, 14.

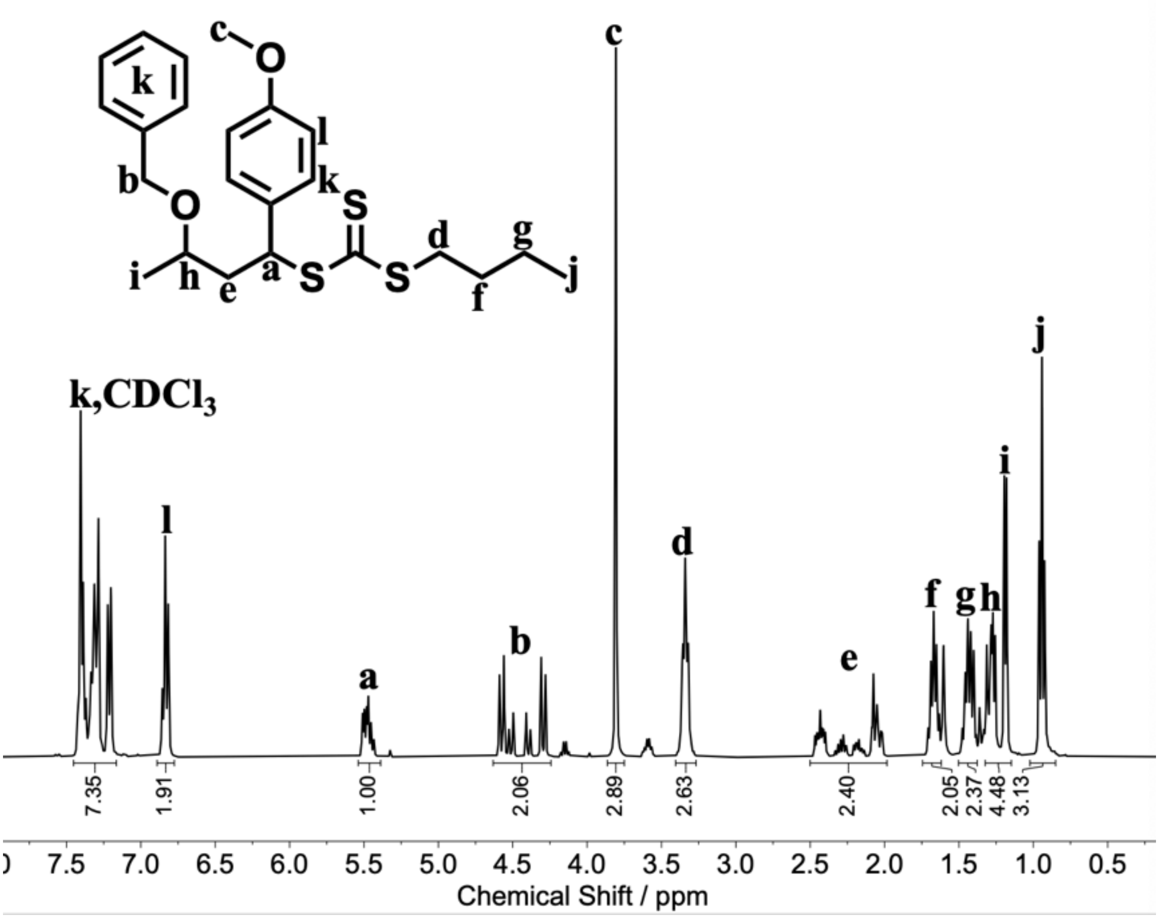

Supplementary Figure 40. ^{1}H NMR spectrum for **15-TTC** in $CDCl_3$.

16-TTC: A yellow solid (yield: 85%). ^{1}H NMR ($CDCl_3$, δ, ppm): 7.1-7.2 and 6.8 (4H, aromatic proton), 5.4-5.5 (1H, $-CH_2C\underline{\mathbf{H}}(-Ph-)S-$), 3.8 (3H, $-OC\underline{\mathbf{H}}_3$), 2.8-3.2 and 3.2-3.5 (6H, $-OC\underline{\mathbf{H}}_2CH(CH_3)_2$, $CH_3C\underline{\mathbf{H}}(-O-)CH_2C\underline{\mathbf{H}}(-O-)CH_2-$, $-OC\underline{\mathbf{H}}_2CH_2CH_2CH_3$), 3.3 (2H, $-SC\underline{\mathbf{H}}_2CH_2CH_2CH_3$)， 2.0-2.5 (4H, $CH_3CH(-O-)C\underline{\mathbf{H}}_2CH(-O-)C\underline{\mathbf{H}}_2CH(-Ph-)-$), 1.9 (3H, $C\underline{\mathbf{H}}_3CH(-O-)CH_2-$), 0.8-1.0 (12H, $-OCH_2CH(C\underline{\mathbf{H}}_3)_2$, $-OCH_2CH_2CH_2C\underline{\mathbf{H}}_3$, $-SCH_2CH_2CH_2C\underline{\mathbf{H}}_3$). ^{13}C NMR ($CDCl_3$, δ, ppm): 130, 114, 76, 74, 69, 68, 55, 41, 36, 30, 22, 20, 14. ESI-MS: m/z calc. for $C_{26}H_{44}O_3S_3Na$: 523.82 $[M+Na]^+$; found: 523.82.

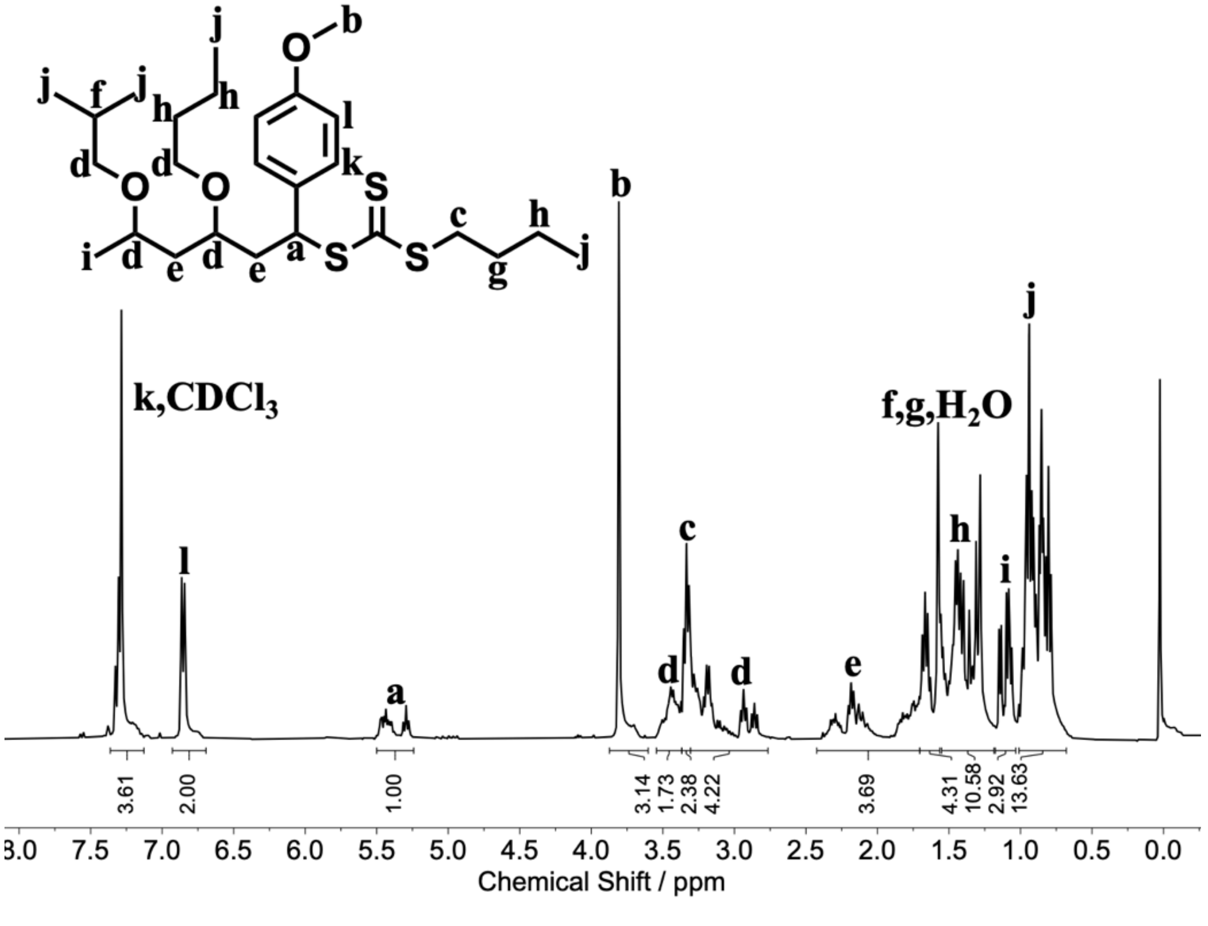

Supplementary Figure 41. ^{1}H NMR spectrum for **16-TTC** in $CDCl_3$.

17-TTC: A yellow solid (yield: 83 %). ^{1}H NMR ($CDCl_3$, δ, ppm): 6.7-8.1 (9H, aromatic proton), 5.0-5.5 (1H, -C**H**(-Ph)S-), 4.5 (2H, -COOC**H**$_2$-), 2.7-4.0 (11H, -SC**H**$_2$-, -COOCH$_2$C**H**$_2$O-, -C**H**(O-)CH$_3$, -C**H**$_2$O-, and -OC**H**$_3$), 1.5-2.2 (4H, >CHC**H**$_2$CH<, >CHC**H**(CH$_3$)CH<, and -C**H**(CH$_3$)$_2$), 0.7-1.3 (44H, -N(CH$_2$C**H**$_3$)$_2$, -CH(O-)C**H**$_3$, >CHCH(C**H**$_3$)CH<, -(C**H**$_2$)$_{10}$-, and -CH(C**H**$_3$)$_2$). ^{13}C NMR ($CDCl_3$, δ, ppm): 166, 157, 134, 133, 130, 129, 113, 76, 75, 72, 67, 64, 49, 46, 45, 44, 29, 27, 20, 13, 12. ESI-MS: m/z calc. for $C_{40}H_{63}O_5S_3$: 719.38 [M+H]$^+$; found: 719.38.

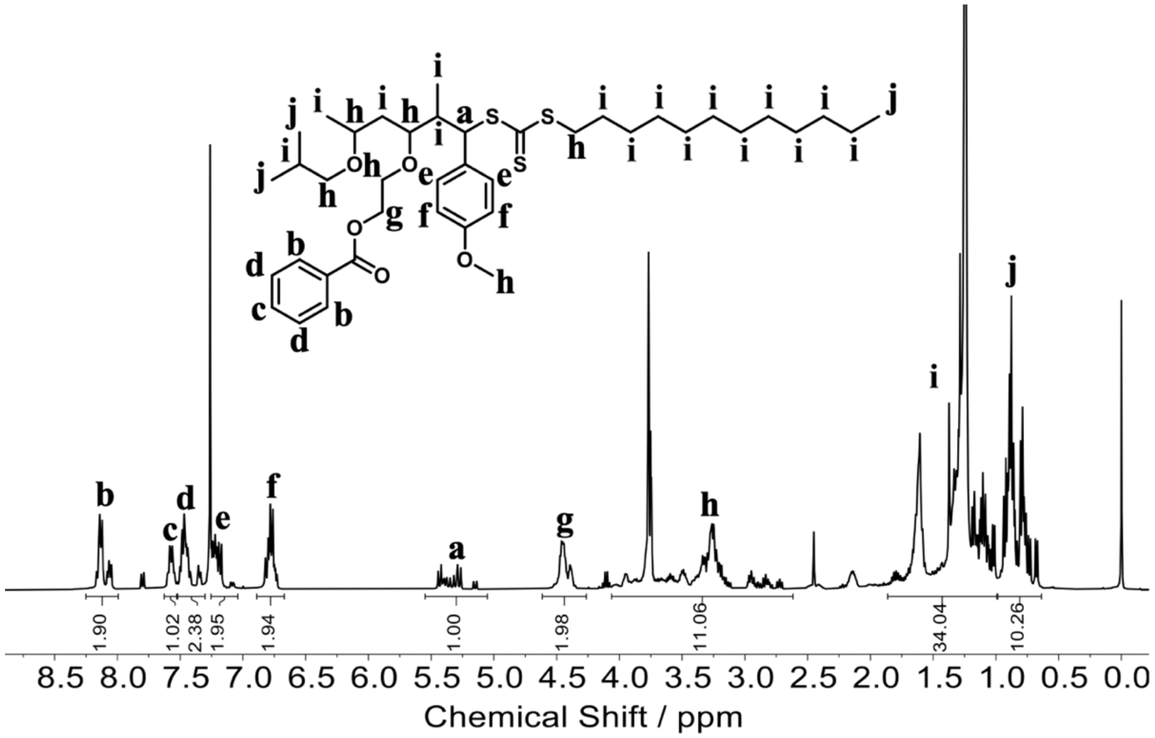

Supplementary Figure 42. ^{1}H NMR spectrum for **17-TTC** in $CDCl_3$.

Insertion maleimide into trithiocarbonate which sythesized from dithiocarbamate

In a nitrogen filled glove box, trithiocarbonates (**12-TTC or 17-TTC**, 10 mmol), maleimide (10 mmol), AIBN (0.5 mmol), toluene (10 mL) were charged into an oven-dried 50 mL Schlenk tube equipped with a stir magneton. After being stirred for 24 h at 70 °C, the crude product was purified by flash chromatography using PE/EA as the eluent.

18-TTC: A yellow solid (yield: 92 %). ^{1}H NMR ($CDCl_3$, δ, ppm): 6.7-8.1 (14H, aromatic proton), 4.5-5.5 (1H, -C**H**(-CO-)S-), 4.2-4.5 (2H, -COOC**H**$_2$-), 2.5-4.0 (15H, -SC**H**$_2$-, -COOCH$_2$C**H**$_2$O-, -C**H**(O-)CH$_3$, -C**H**$_2$O-, and -OC**H**$_3$), 0.5-1.7 (51H, >CHC**H**$_2$CH<, >CHC**H**(CH$_3$)CH<, -C**H**(CH$_3$)$_2$, -N(CH$_2$C**H**$_3$)$_2$, -CH(O-)C**H**$_3$, -(C**H**$_2$)$_{10}$-, and -CH(C**H**$_3$)$_2$). ^{13}C NMR ($CDCl_3$, δ, ppm): 175, 171, 166, 157, 134, 133, 130, 129, 113, 76, 75, 72, 67, 64, 49, 46, 45, 44, 29, 27, 20, 13, 12. ESI-MS: m/z calc. for $C_{50}H_{70}NO_7S_3$: 892.41 [M+H]$^+$; found: 892.41.

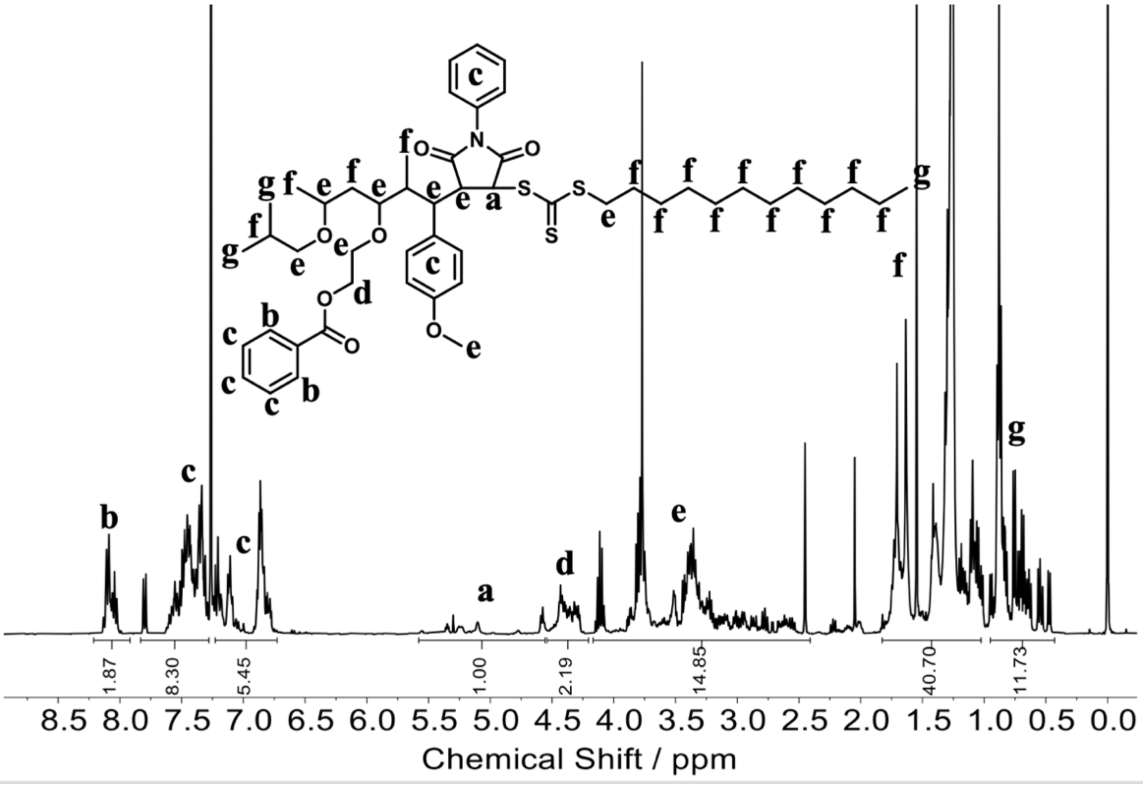

Supplementary Figure 43. ^{1}H NMR spectrum for **18-TTC** in $CDCl_3$.

19-TTC: A yellow solid (yield: 88%). ^{1}H NMR ($CDCl_3$, δ, ppm): 7.1-7.2 and 6.8 (4H, aromatic proton), 4.5-5.0 (1H, >C**H**S-), 3.8 (3H,-OC**H**$_3$), 2.9-3.6 (8H, CH_3C**H**$_2$N-, -SC**H**$_2CH_2CH_2CH_3$, -CH(-Ph)C**H**<, -OC**H**$_2CH(CH_3)_2$, CH_3C**H**(-O-)-), 2.4-2.8 (1H,-CH_2C**H**(-Ph)CH<), 1.8-2.4 (3H, -C**H**$_2$CH(-Ph)CH<, -OCH_2C**H**$(CH_3)_2$), 1.7 and 1.4-1.5 (4H, -SCH_2 C**H**$_2$ C**H**$_2$ CH_3), 1.1-1.3 (6H, -NCH_2C**H**$_3$, C**H**$_3$CH(-O-)-), 0.8-1.0 (9H, -OCH_2CH(C**H**$_3$)$_2$, -$SCH_2CH_2CH_2$C**H**$_3$). ^{13}C NMR ($CDCl_3$, δ, ppm): 176, 173, 160, 129, 128, 125, 115, 75, 73, 55, 53, 50, 43, 42, 38, 34, 30, 29, 23, 20, 14, 13. ESI-MS: m/z calc. for $C_{26}H_{40}NO_4S_3$: 526.72. $[M+H]^+$; found: 526.72.

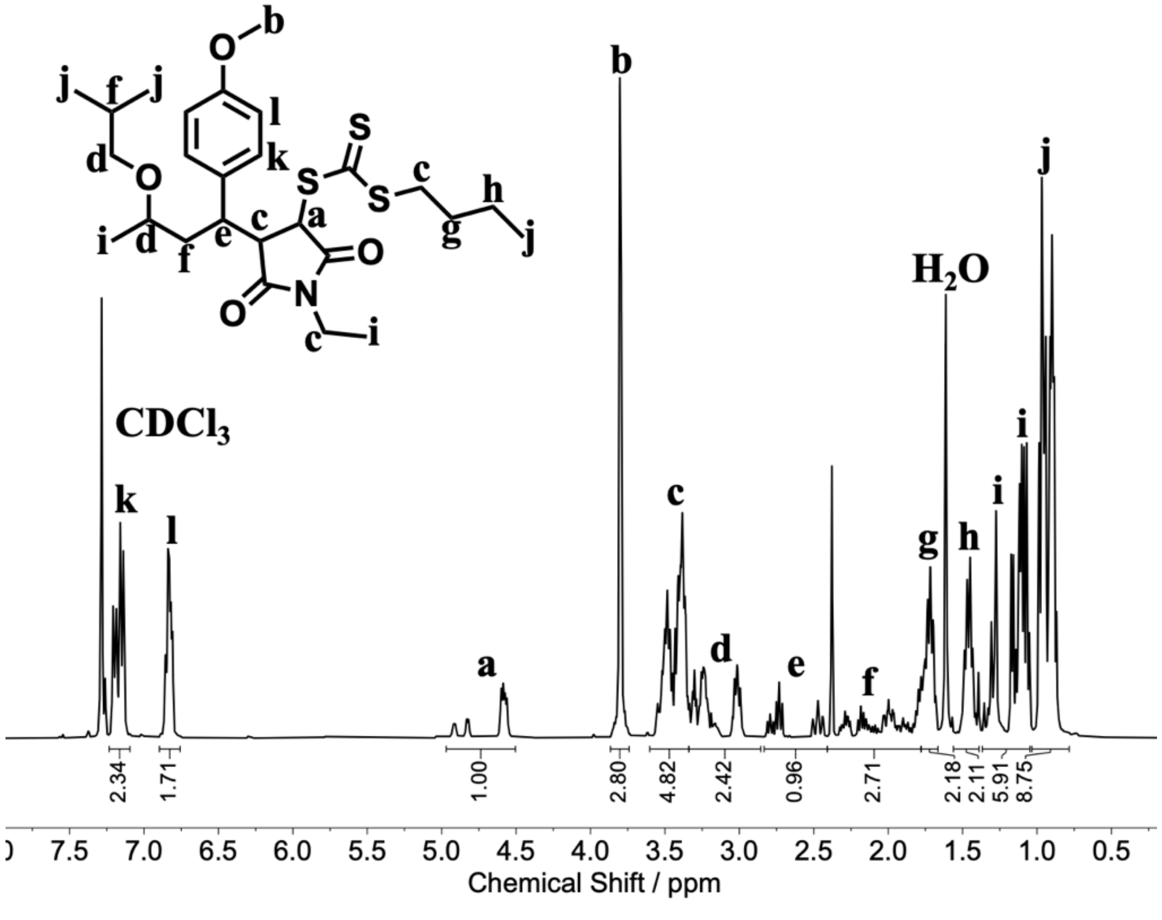

Supplementary Figure 44. ^{1}H NMR spectrum for **19-TTC** in $CDCl_3$.

Insertion iBVE into 19-TTC via second radical SUMI

In a nitrogen filled glove box, **19-TTC** (10 mmol), iBVE (10 mmol), AIBN (0.5 mmol), toluene (10 mL) were charged into an oven-dried 50 mL Schlenk tube equipped with a stir magneton. After being stirred for 24 h at 70 °C, the crude product was purified by flash chromatography using PE/EA as the eluent. affording a yellow solid (yield: 82%). ^{1}H NMR ($CDCl_3$, δ, ppm): 7.1-7.2 and 6.8 (4H, aromatic proton), 6.0-6.2 (1H, $-CH_2C\underline{\mathbf{H}}(-O-)S-$), 3.7-3.8 (3H,$-OC\underline{\mathbf{H}}_3$), 2.7-3.6 (11H, $CH_3C\underline{\mathbf{H}}_2N<$, $-SC\underline{\mathbf{H}}_2CH_2CH_2CH_3$, $-CH(-Ph)C\underline{\mathbf{H}}(-CO)C\underline{\mathbf{H}}<$, $-OC\underline{\mathbf{H}}_2CH(CH_3)_2$, $-OC\underline{\mathbf{H}}_2CH(CH_3)_2$, $CH_3C\underline{\mathbf{H}}(-O-)-$), 1.8-2.6 (7H, $-C\underline{\mathbf{H}}_2C\underline{\mathbf{H}}(-Ph)CH<$, $-OCH_2C\underline{\mathbf{H}}(CH_3)_2$, $-OCH_2C\underline{\mathbf{H}}(CH_3)_2$, $-C\underline{\mathbf{H}}_2CH(-O-)S-$), 1.7 and 1.5 (4H, $-SCH_2\ C\underline{\mathbf{H}}_2\ C\underline{\mathbf{H}}_2\ CH_3$), 1.1-1.3 (6H, $-NCH_2C\underline{\mathbf{H}}_3$, $C\underline{\mathbf{H}}_3CH(-O-)-$), 0.8-1.1 (15H, $-OCH_2CH(C\underline{\mathbf{H}}_3)_2$, $-OCH_2CH(C\underline{\mathbf{H}}_3)_2$, $-SCH_2CH_2CH_2C\underline{\mathbf{H}}_3$). ^{13}C NMR ($CDCl_3$, δ, ppm):176, 160, 131, 130, 129, 114, 55, 42, 36, 30, 29, 22, 20, 14. ESI-MS: m/z calc. for $C_{32}H_{51}NO_5S_3Na$: 648.91 $[M+Na]^+$; found: 648.92.

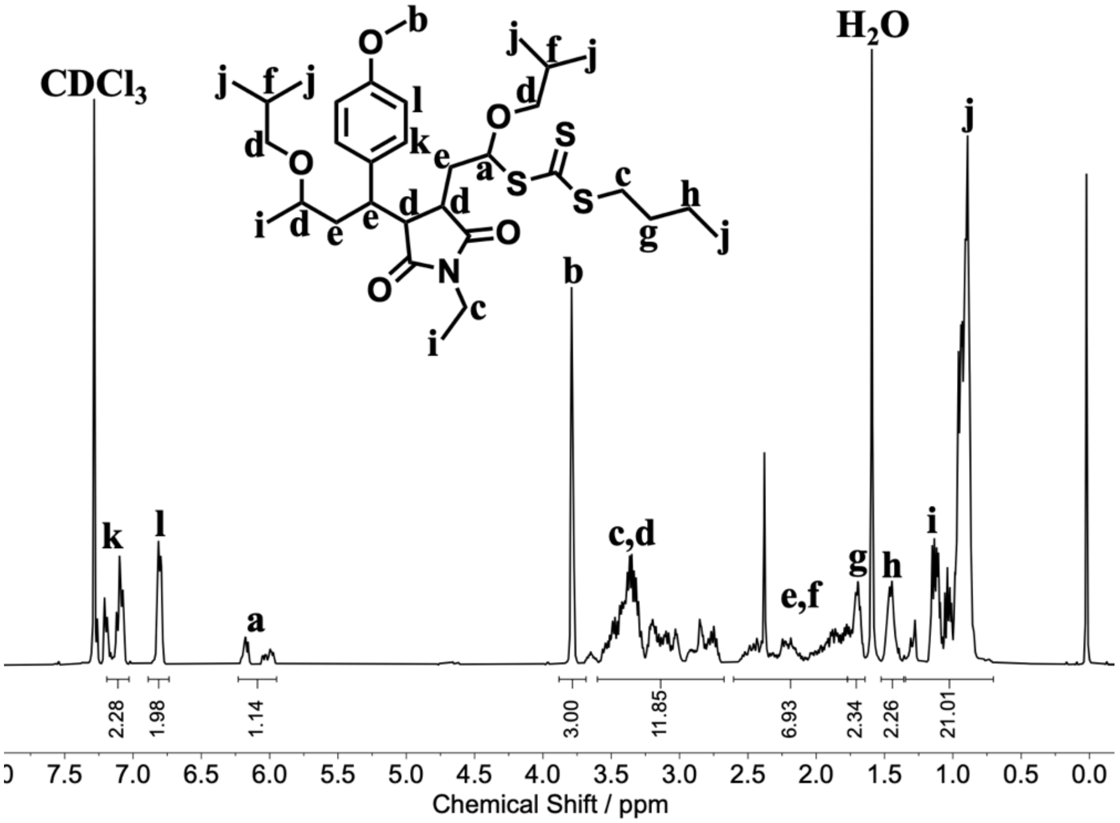

Supplementary Figure 45. ^{1}H NMR spectrum for **20-TTC** in $CDCl_3$.

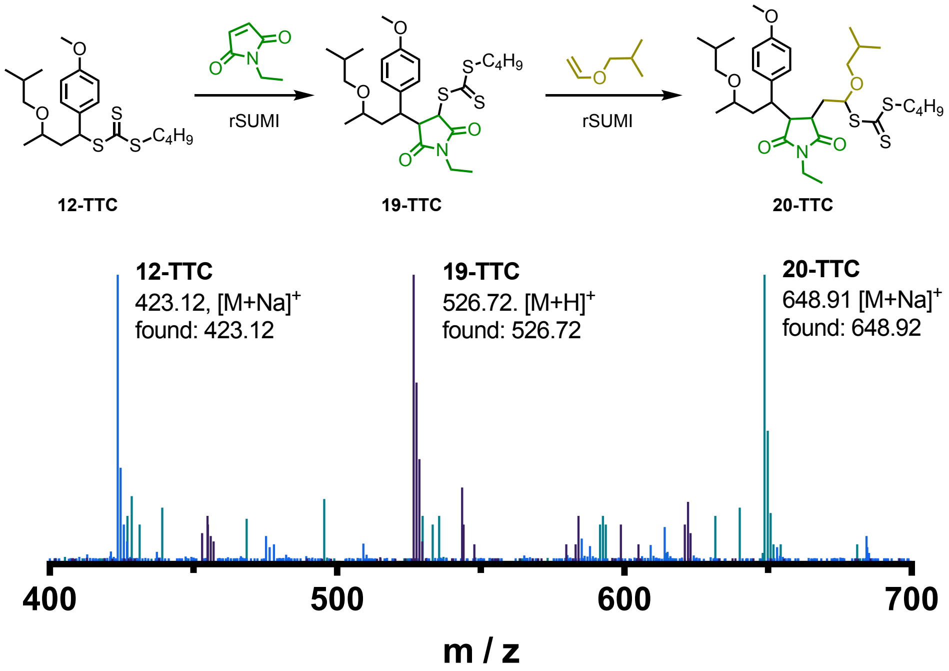

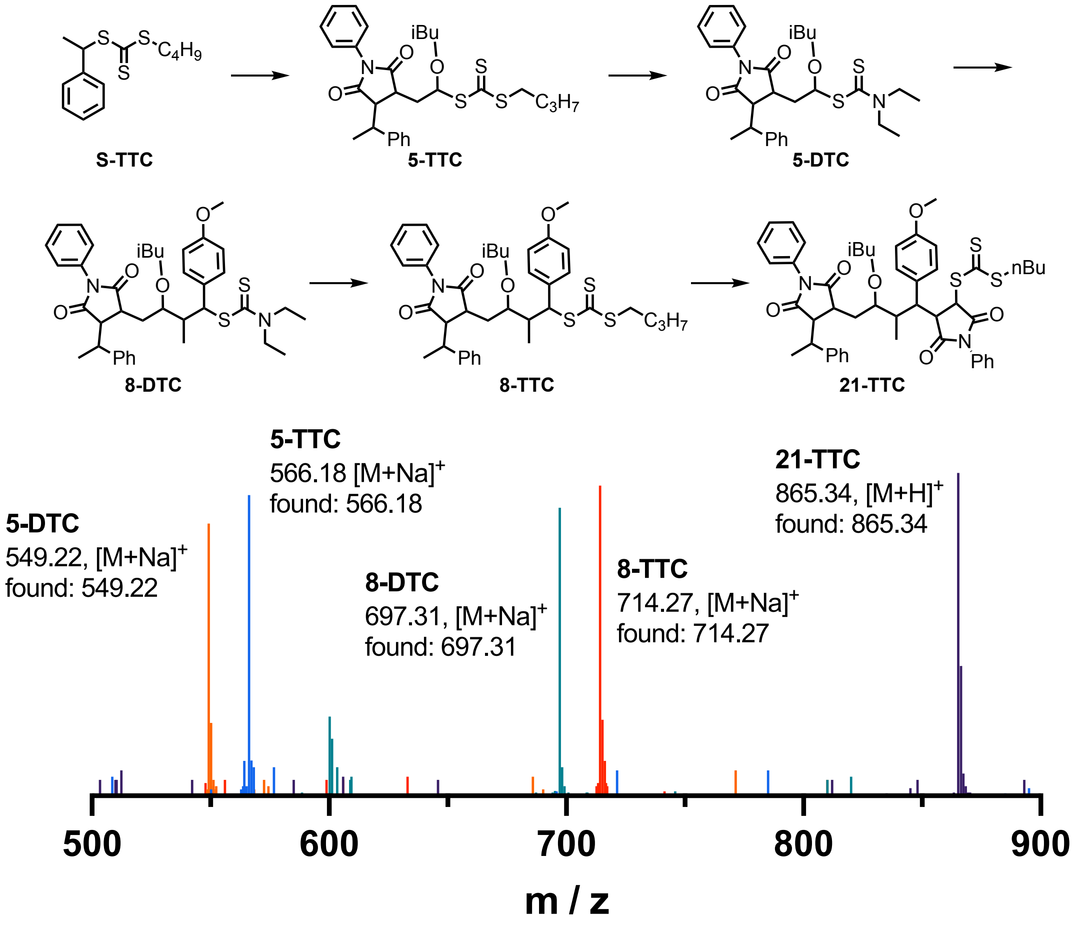

Supplementary Figure 46. ESI MS spectra for **5-DTC**, **5-TTC**, **8-DTC**, **8-TTC**, **12-TTC, 19-TTC**, **20-TTC**, and **21-TTC**.

Synthesis of 8-TTC. In a nitrogen filled glove box, **8-DTC** (1 equiv), BBTD (5 equiv), $FcPF_6$ (5 mol %), and DCM were charged into an oven-dried 20 mL Schlenk tube equipped with a stir magneton. The mixture was stirred at room temperature for 24 h. The crude product was purified by flash chromatography using PE/EA as the eluent affording a yellow solid (yield: 85%).^{1}H NMR ($CDCl_3$, δ, ppm): 6.9-7.5 (14H, aromatic proton), 5.2-5.6 (1H, -C**H**(-Ph)S-), 4.0-3.2 (4H, -OC**H**$_3$ and Ph-C**H**<), 3.3 (2H, -SC**H**$_2$-), 3.2-2.6 (4H, >C**H**CO-, and -OC**H**$_2$-), 2.2-0.9 (26H, >CHC**H**$_2$CH< and >CHC**H**(CH_3)CH<, -CH_3, -N(CH_2C**H**$_3$)$_2$, and -C**H**$_3$). ^{13}C NMR ($CDCl_3$, δ, ppm): 178, 177, 159, 142, 141, 135, 132, 131, 129, 128, 127, 114, 58, 55, 52, 50, 48, 46, 40, 39, 38 37, 28, 26, 19, 18, 16, 13, 11. ESI-MS: m/z calc. for $C_{39}H_{49}NO_4S_3Na$: 714.27, $[M+Na]^+$; found: 714.27.

Synthesis of 21-TTC. In a nitrogen filled glove box, **8-TTC** (10 mmol), PMI (12 mmol), AIBN (0.5 mmol), toluene (10 mL) were charged into an oven-dried 50 mL Schlenk tube equipped with a stir magneton. After being stirred for 24 h at 70 °C, the crude product was purified by flash chromatography using PE/EA as the eluent affording **21-TTC** as a yellow solid (yield: 76%).^{1}H NMR ($CDCl_3$, δ, ppm): 6.7-7.5 (19H, aromatic proton), 4.4-5.3 (1H, -C**H**(CO-)S-), 3.7-3.6 (4H, -OC**H**$_3$ and Ph-C**H**<), 2.5-3.5 (8H, -SC**H**$_2$-, >C**H**CO-, and -OC**H**$_2$-), 2.2-0.9 (30H, >CHC**H**$_2$CH< and >CHC**H**(CH_3)CH<, -CH_3, -N(CH_2C**H**$_3$)$_2$, and -C**H**$_3$). ESI-MS: m/z calc. for $C_{49}H_{57}N_2O_6S_3$: 865.34, $[M+H]^+$; found: 865.34.

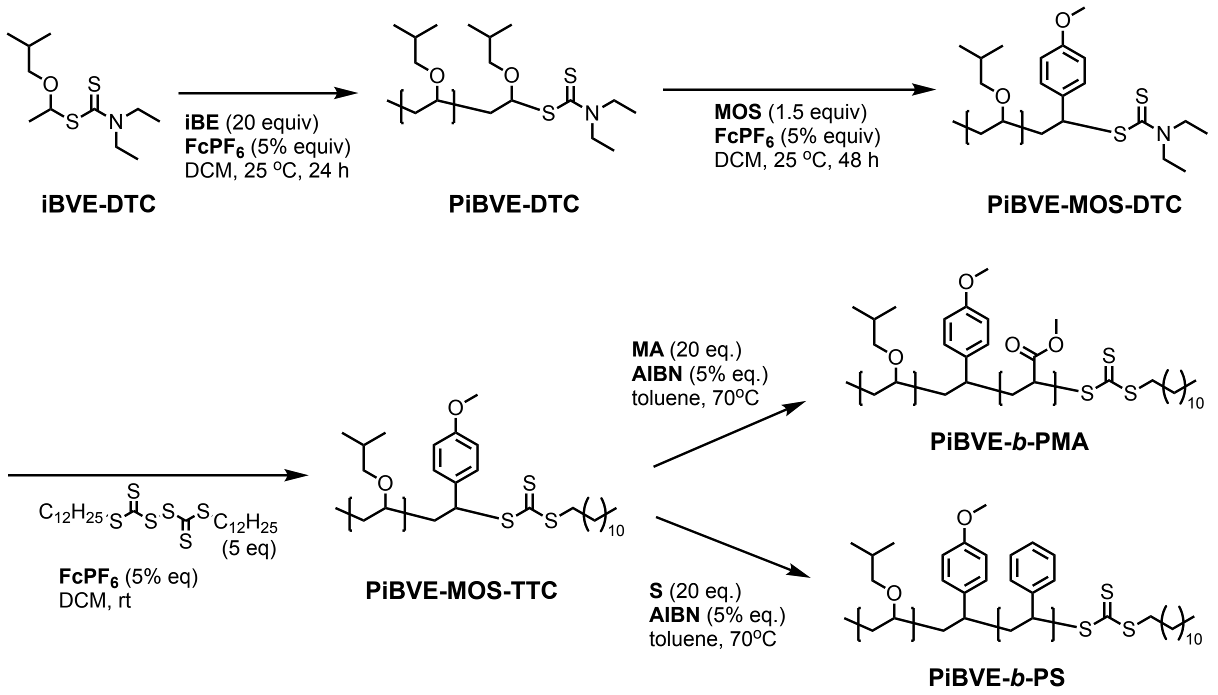

Supplementary Figure 47. Synthesis of PiBVE-*b*-PMA and PiBVE-*b*-PS diblock polymer.

Synthesis of PiBVE-DTC.

In a nitrogen filled glove box, iBVE-DTC (1 g, 4 mmol), iBVE (8 g, 80 mmol), $FcPF_6$ (13.3 mg, 0.04 mmol), anhydrous DCM (16 mL) were charged into an oven-dried 50 mL Schlenk tube equipped with a stir magneton. After being stirred for 12 h at 25 °C, the reaction tube was quenched by adding sodium diethyldithiocarbamate (100 mg). The crude product was purified by silica gel column chromatography using EtOAc/hexane (v/v = 1/5) as the eluent, affording PiBVE as a white solid (8.8 g, yield: 98%). The DP was determined to be ~20 by ^{1}H NMR analysis in $CDCl_3$ according $I_d/3I_a$, where I_d is integral ratio of proton resonance signal of peak d (>C**H**OC**H**$_2$- in **iBVE** units), I_a is that of peak a (>C**H**SCS- close to **DTC** residues). The $M_{n,NMR}$ was determined to be 2,250 Da by ^{1}H NMR analysis in $CDCl_3$ according DP*$M_{\mathbf{iBVE}}$ + $M_{\mathbf{DTC}}$, where M_{iBVE} and M_{DTC} are molecular weights of iBVE monomer and **DTC**, respectively. The molecular weight and molecular weight distribution of the product were determined by GPC using THF as the eluent, revealing an $M_{n,GPC}$ of 2,060 Da and M_w/M_n of 1.12.

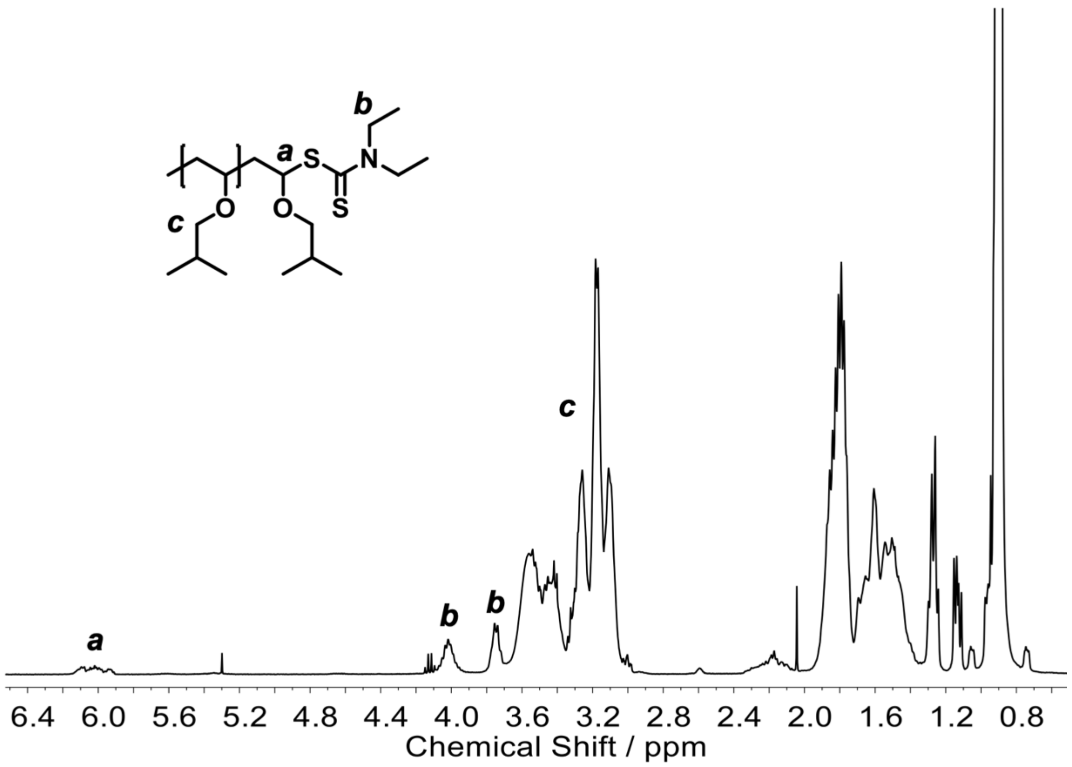

Supplementary Figure 48. ^{1}H NMR spectrum in $CDCl_3$ for **PiBVE-DTC.**

Synthesis of PiBVE-MOS-DTC.

In a nitrogen filled glove box, **PiBVE-DTC** (0.38 mmol), **MOS** (0.56 mmol), $FcPF_6$ (0.019 mmol), anhydrous DCM (2 mL) were charged into an oven-dried 20 mL Schlenk tube equipped with a stir magneton. After being stirred for 48 h at 25 ^{o}C, the reaction tube was quenched by adding sodium diethyldithiocarbamate (10 mg). The solvent and monomer were removed under reduced pressure. The crude product was purified by silica gel column chromatography using EtOAc/hexane (v/v = 1/5) as the eluent, affording **PiBVE-MOS-DTC** as a white solid (yield: 89%). The molecular weight and molecular weight distribution of the product were determined by GPC using THF as the eluent, revealing an M_n of 2,220 and M_w/M_n of 1.16. The extent of **MOS** functionalization was determined to be ~85% by ^{1}H NMR analysis in $CDCl_3$ according $I_a/5I_c$, where I_a is integral ratio of proton resonance signal of peak a (**MOS** residues), I_c is that of peak a (>C**H**SCS- close to **DTC** residues).

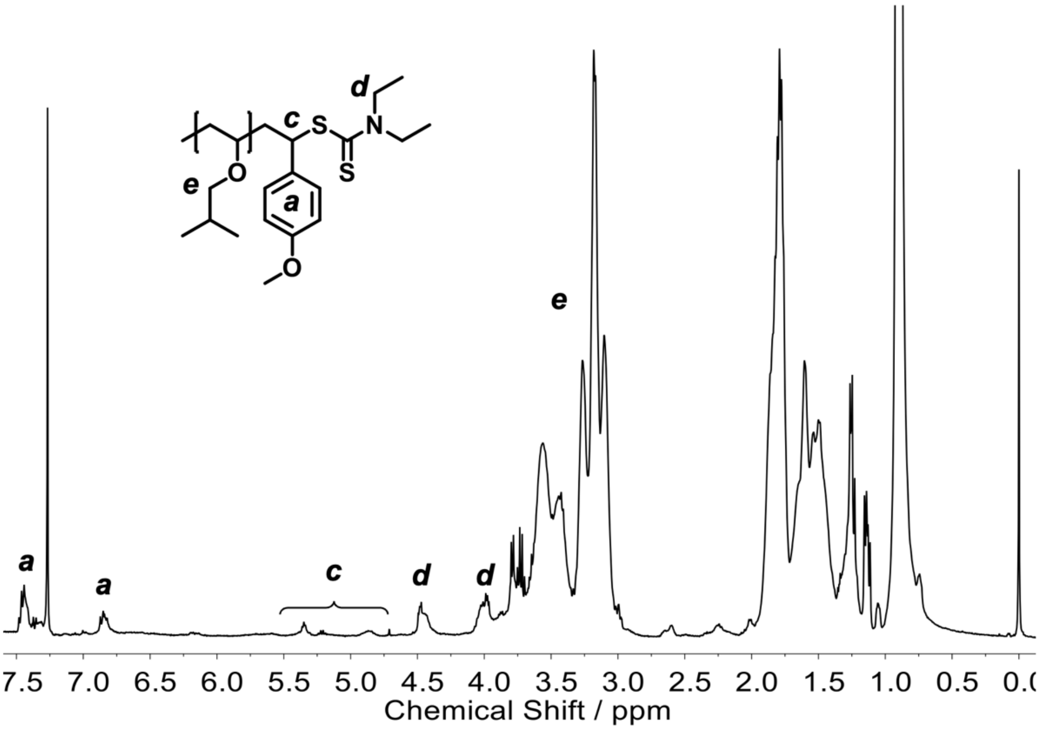

Supplementary Figure 49. ^{1}H NMR spectrum in $CDCl_3$ for **PiBVE-MOS-DTC.**

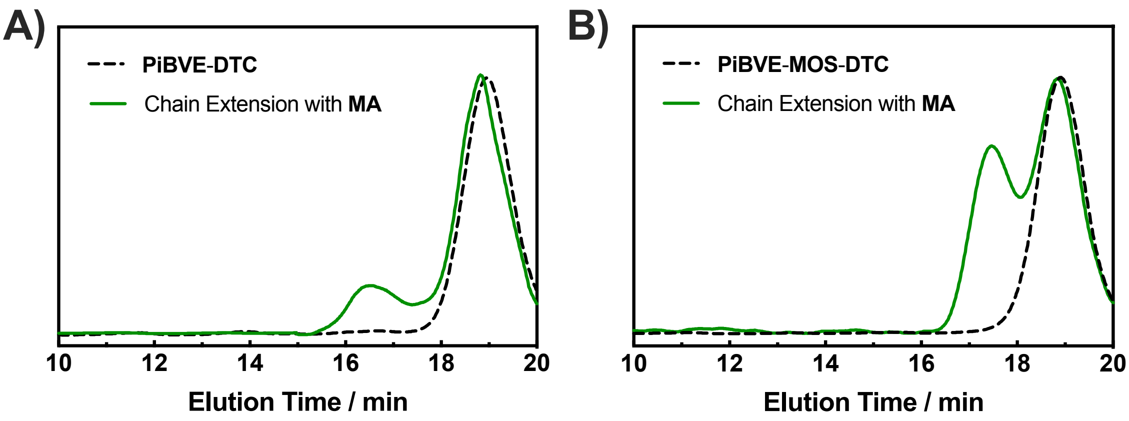

Supplementary Figure 50. GPC traces for (a) PiBVE-DTC, (b) PiBVE-MOS-DTC, and their chain extension with MA**.**

Synthesis of PiBVE-MOS-TTC.

In a nitrogen filled glove box, **PiBVE-MOS-DTC** (0.3 mmol), **BDTD** (1.5 mmol), $FcPF_6$ (0.015 mmol), anhydrous DCM (4 mL) were charged into an oven-dried 20 mL Schlenk tube equipped with a stir magneton. After being stirred for 48 h at 25 °C. The solvent and monomer were removed under reduced pressure. The crude product was purified by silica gel column chromatography using EtOAc/hexane (*v*/*v* = 1/5) as the eluent, affording **PiBVE-MOS-TTC** as a yellow solid (yield: 91%). The molecular weight and molecular weight distribution of the product were determined by GPC using THF as the eluent, revealing an M_n of 2,420 and M_w/M_n of 1.18.

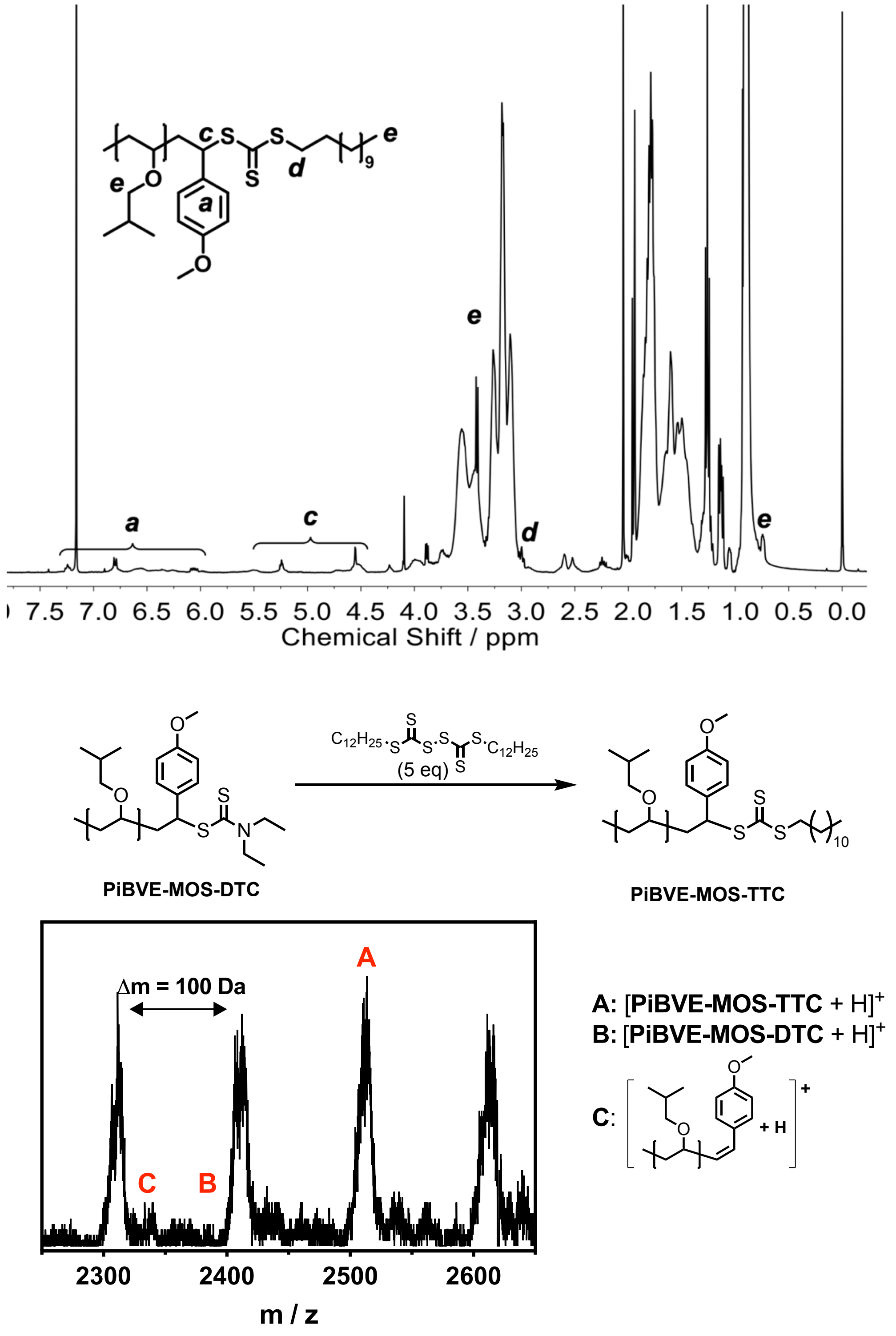

Supplementary Figure 51. ^{1}H NMR spectrum in $CDCl_3$ and MALDI-TOF mass spectra for **PiBVE-MOS-TTC.**

Synthesis of PiBVE-b-PMA or PiBVE-b-PS.

In a nitrogen filled glove box, **PiBVE-MOS-TTC** (0.2 mmol), **MA** or styrene (4 mmol), AIBN (0.04 mmol), toluene (2 mL) were charged into an oven-dried 20 mL Schlenk tube equipped with a stir magneton. After being stirred for 24 h at 70 ^{o}C, mixture was precipitated into an excess of MeOH/H_2O. The above dissolution-precipitation cycle was repeated for three times. The final product was dried in a vacuum oven overnight at room temperature, yielding a yellow solid.

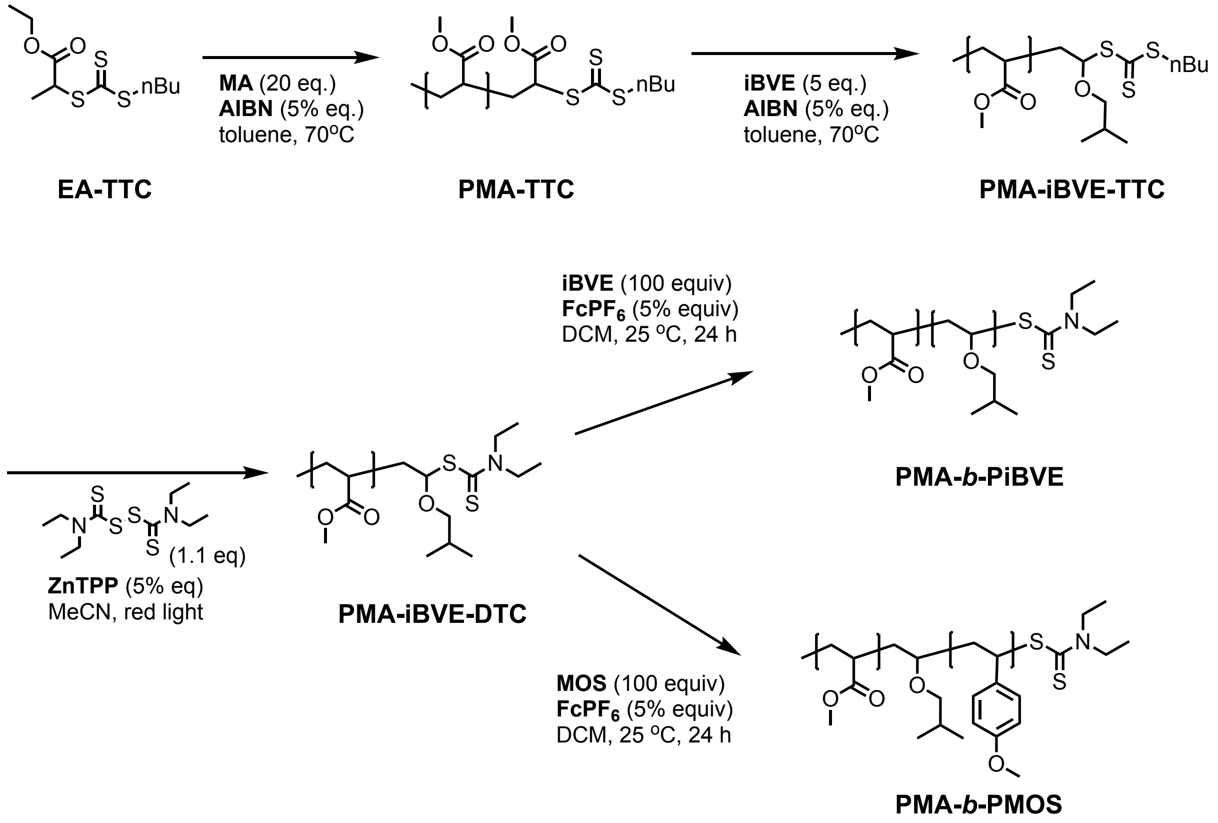

Supplementary Figure 52. Synthesis of PMA-*b*-PiBVE and PMA-*b*-PMOS diblock polymers.

Synthesis of PMA-TTC.

In a nitrogen filled glove box, **EA-TTC** (2 mmol), **MA** (40 mmol), AIBN (0.4 mmol), toluene (4 mL) were charged into an oven-dried 20 mL Schlenk tube equipped with a stir magneton. After being stirred for 24 h at 70 °C, mixture was precipitated into an excess of *n*-hexane. The above dissolution-precipitation cycle was repeated for three times. The final product was dried in a vacuum oven overnight at room temperature, yielding a yellow solid (yield: 71.9%). The DP was determined to be ~21 by ^{1}H NMR analysis in $CDCl_3$ according $I_b/3I_a$, where I_b is integral ratio of proton resonance signal of peak b (-COOC**H**3 in **MA** units), I_a is that of peak a (>C**H**SCS- close to **TT** residues). The $M_{n,NMR}$ was determined to be 2,060 Da by ^{1}H NMR analysis in $CDCl_3$ according DP*M_{MA} + $M_{EA\text{-}TT}$, where M_{MA} and $M_{EA\text{-}TT}$ are molecular weights of **MA** monomer and **EA-TTC**, respectively. The molecular weight and molecular weight distribution of the product were determined by GPC using THF as the eluent, revealing an $M_{n,GPC}$ of 2,250 Da and M_w/M_n of 1.12.

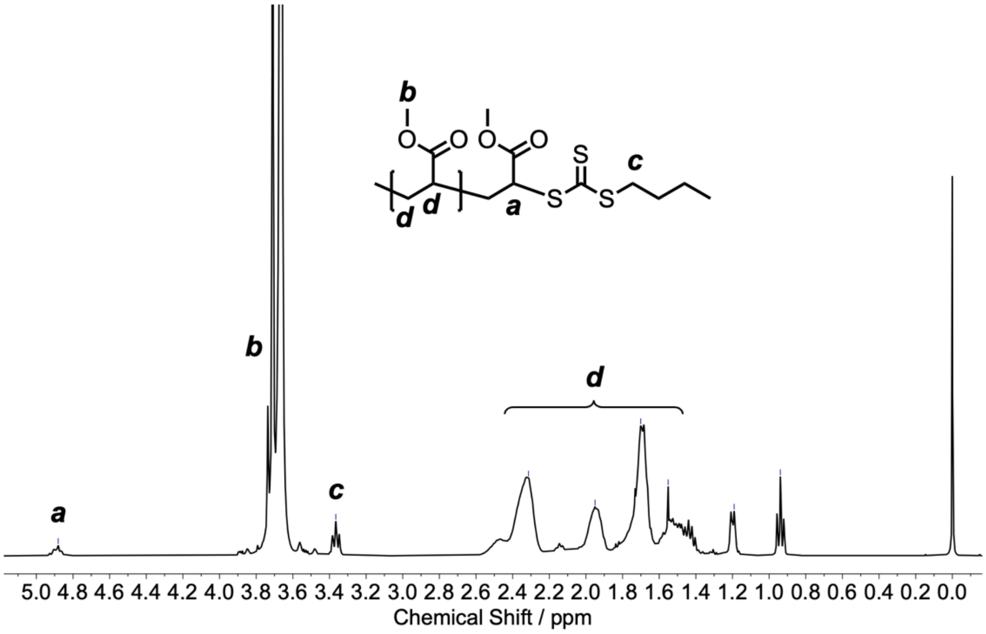

Supplementary Figure 53. ^{1}H NMR spectrum in $CDCl_3$ for **PMA-TTC.**

Synthesis of PMA-iBVE-TTC.

In a nitrogen filled glove box, **PMA-TTC** (1 mmol), **iBVE** (5 mmol), AIBN (0.05 mmol), toluene (8 mL) were charged into an oven-dried 20 mL Schlenk tube equipped with a stir magneton. After being stirred for 24 h at 70 °C, mixture was precipitated into an excess of *n*-hexane. The above dissolution-precipitation cycle was repeated for three times. The final product was dried in a vacuum oven overnight at room temperature, yielding a yellow solid (yield: 65.9%). The molecular weight and molecular weight distribution of the product were determined by GPC using THF as the eluent, revealing an M_n of 2,410 and M_w/M_n of 1.15. The extent of **iBVE** functionalization was determined to be ~89% by ^{1}H NMR analysis in $CDCl_3$ according $2I_a/I_c$, where I_c is integral ratio of proton resonance signal of peak a (-SCSC**H**$_2$CH$_3$- in **TTC** residues), I_a is that of peak a (>C**H**SCS- close to **TTC** residues).

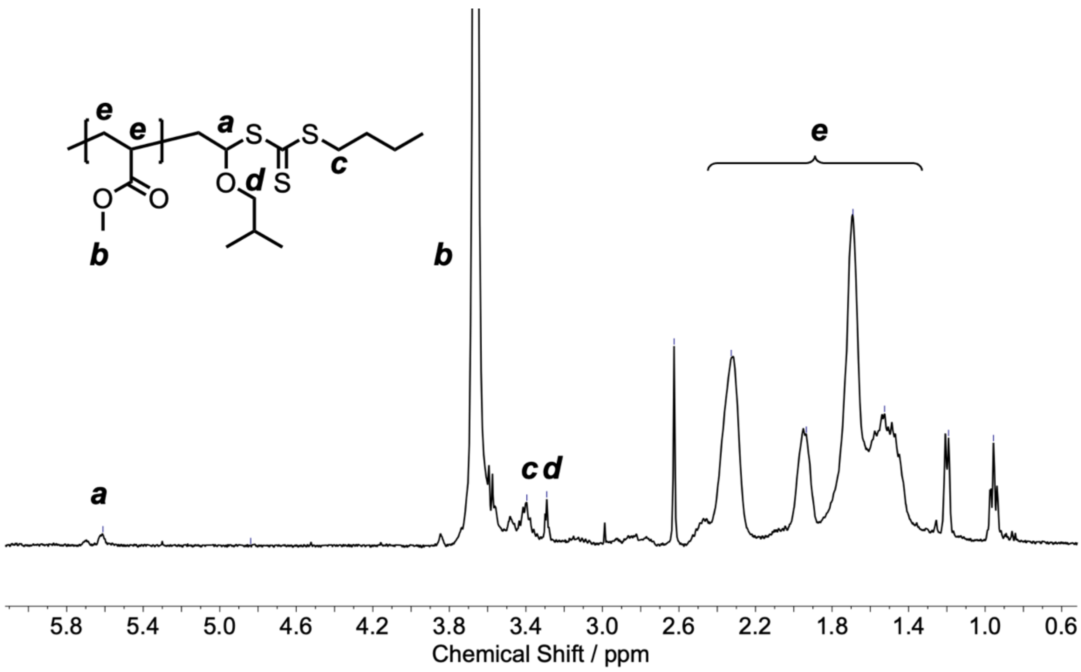

Supplementary Figure 54. ^{1}H NMR spectrum in $CDCl_3$ for **PMA-iBVE-TTC.**

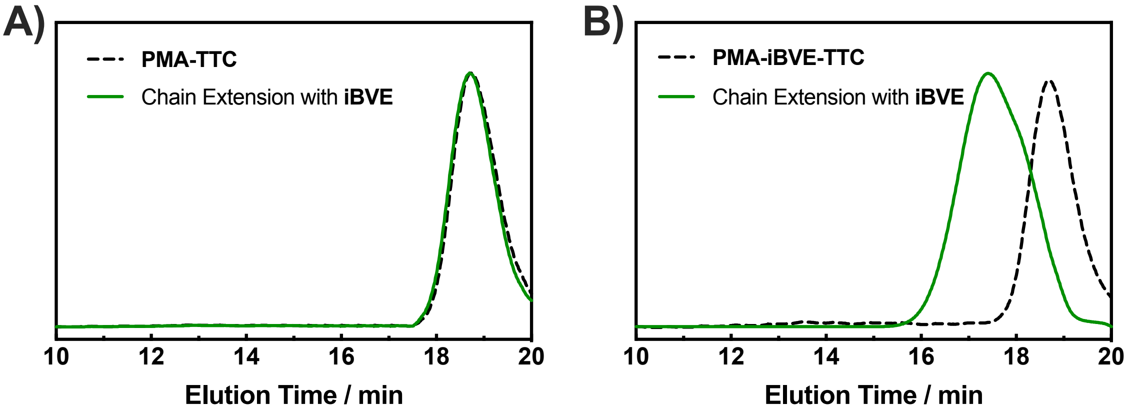

Supplementary Figure 55. GPC traces for (a) PMA-TTC, (b) PMA-iBVE-TTC, and their chain extension with iBVE**.**

Synthesis of PMA-iBVE-DTC

In a nitrogen filled glove box, **PMA-iBVE-TTC** (0.5 mmol), TETD (0.55 mmol), ZnTPP (0.01 mmol), MeCN (4 mL) were charged into an oven-dried 20 mL Schlenk tube equipped with a stir magneton. The mixture was stirred under red light irradiation (630 nm) at room temperature for 48 h. Mixture was precipitated into an excess of *n*-hexane. The above dissolution-precipitation cycle was repeated for three times. The crude product was purified by silica gel column chromatography using EtOAc/hexane (*v*/*v* = 1/3) as the eluent, yielding a yellow solid (yield: 66.2%). The molecular weight and molecular weight distribution of the product were determined by GPC using THF as the eluent, revealing an M_n of 2,440 and M_w/M_n of 1.15. The extent of **iBVE** functionalization was determined to be ~87% by ^{1}H NMR analysis in $CDCl_3$ according $I_b/2I_a$, where I_b is integral ratio of proton resonance signal of peak b (-SCSN(C**H**$_2$CH$_3$)$_2$ in **DTC** residues), I_a is that of peak a ((>C**H**SCS- close to **DTC** residues).

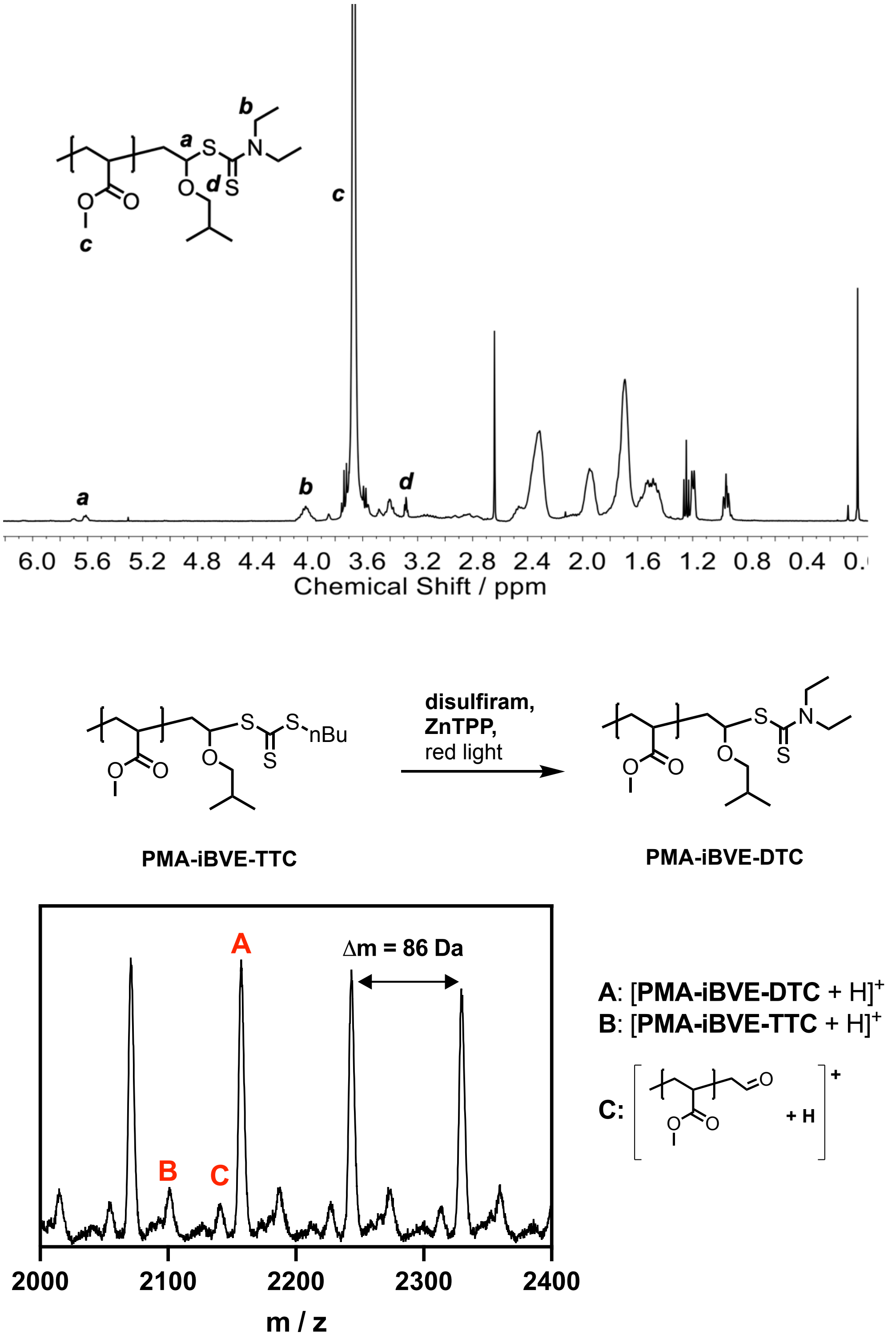

Supplementary Figure 56. ^{1}H NMR spectrum in $CDCl_3$ and MALDI-TOF MS for **PMA-iBVE-DTC.**

Synthesis of PMA-b-PiBVE and PMA-b-PMOS.

In a nitrogen filled glove box, **PMA-iBVE-DTC** (0.4 mmol), **iBVE** or **MOS** (8 mmol), $FcPF_6$ (0.02 mmol), anhydrous DCM (4 mL) were charged into an oven-dried 50 mL Schlenk tube equipped with a stir magneton. After being stirred for 12 h at 25 °C, the reaction tube was quenched by adding sodium diethyldithiocarbamate (10 mg). The crude product was purified by silica gel column chromatography using EtOAc/hexane (*v*/*v* = 1/2) as the eluent, affording **PMA-b-PiBVE** as a white solid.

References

[1] C. Lv, C. He, X. Pan, *Angew. Chem. Int. Ed.* **2018**, *57*, 9430-9433.

[2] N. Haridharan, K. Ponnusamy, R. Dhamodharan, *J. Polym. Sci. A: Polym. Chem.* **2010**, *48*, 5329-5338.

[3] S. Kumagai, K. Nagai, K. Satoh, M. Kamigaito, *Macromolecules* **2010**, *43*, 7523-7531.

[4] N. Azizi, F. Aryanasab, L. Tourkian, M. R. Saidi, *Synth. Commun.* **2010**, *41*, 94-99.

[5] V. Kottisch, Q. Michaudel, B. P. Fors, *J. Am. Chem. Soc.* **2016**, *138*, 15535-15538.

[6] Q. Michaudel, T. Chauvire, V. Kottisch, M. J. Supej, K. J. Stawiasz, L. Shen, W. R. Zipfel, H. D. Abruna, J. H. Freed, B. P. Fors, *J. Am. Chem. Soc.* **2017**, *139*, 15530-15538.

[7] K. Ishizu, Y. Ohta, S. Kawauchi, *Macromolecules* **2002**, *35*, 3781-3784.

[8] E. E. Stache, V. Kottisch, B. P. Fors, *J. Am. Chem. Soc.* **2020**, *142*, 4581-4585.

[9] K. Garg, C. Majumder, S. K. Nayak, D. K. Aswal, S. K. Gupta, S. Chattopadhyay, *Phys. Chem. Chem. Phys.* **2015**, *17*, 1891-1899.

[10] T. P. Varner, A. J. Teator, Y. Reddi, P. E. Jacky, C. J. Cramer, F. A. Leibfarth, *J. Am. Chem. Soc.* **2020**, *142*, 17175-17186.

[11] Z. Huang, B. B. Noble, N. Corrigan, Y. Chu, K. Satoh, D. S. Thomas, C. J. Hawker, G. Moad, M. Kamigaito, M. L. Coote, C. Boyer, J. Xu, *J Am Chem Soc* **2018**, *140*, 13392-13406.
